# Development of Cytotoxic GW7604-Zeise’s Salt Conjugates as Multitarget Compounds with Selectivity for Estrogen Receptor- Positive Tumor Cells

**DOI:** 10.1021/acs.jmedchem.3c02454

**Published:** 2024-03-13

**Authors:** Patricia Grabher, Paul Kapitza, Nikolas Hörmann, Amelie Scherfler, Martin Hermann, Michael Zwerger, Hristo P. Varbanov, Brigitte Kircher, Daniel Baecker, Ronald Gust

**Affiliations:** † Department of Pharmaceutical Chemistry, Institute of Pharmacy, Center for Molecular Biosciences Innsbruck, University of Innsbruck, Innrain 80/82, Innsbruck A-6020, Austria; ‡ Department of Anesthesiology & Critical Care Medicine, Medical University Innsbruck, Anichstraße 35, Innsbruck A-6020, Austria; § Department of Pharmacognosy, Institute of Pharmacy, Center for Molecular Biosciences Innsbruck, University of Innsbruck, Innrain 80/82, Innsbruck A-6020, Austria; ∥ Department of Internal Medicine V, Haematology & Oncology, Immunobiology and Stem Cell Laboratory, Medical University Innsbruck, Anichstraße 35, Innsbruck A-6020, Austria; ⊥ Tyrolean Cancer Research Institute, Innrain 66, Innsbruck A-6020, Austria; # Department of Pharmaceutical and Medicinal Chemistry, Institute of Pharmacy, Freie Universität Berlin, Königin-Luise-Straße 2 + 4, Berlin D-14195, Germany

## Abstract

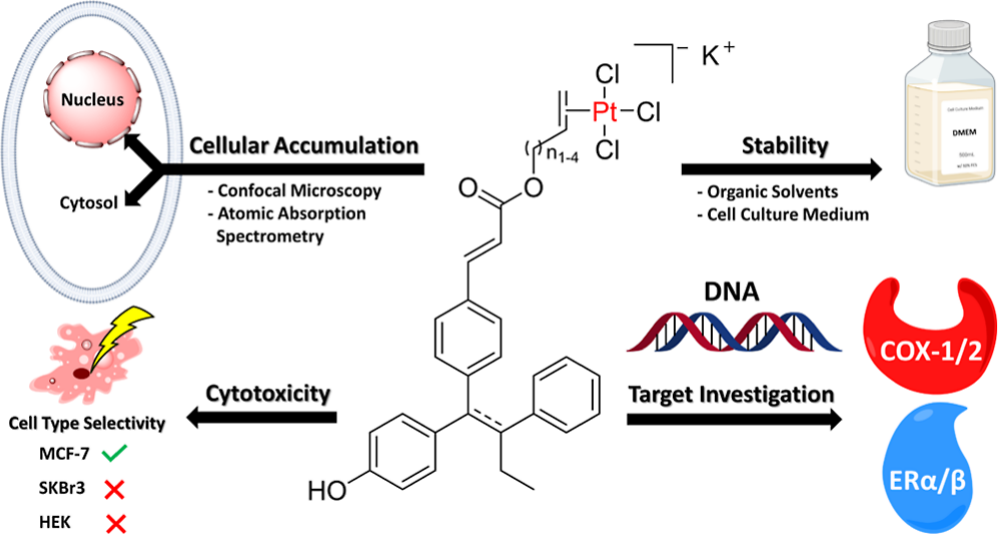

(*E/Z*)-3-(4-((*E*)-1-(4-Hydroxyphenyl)-2-phenylbut-1-enyl)phenyl)acrylic acid (**GW7604**) as a carrier was esterified with alkenols of various lengths and coordinated through the ethylene moiety to PtCl_3_, similar to Zeise’s salt (K[PtCl_3_(C_2_H_4_)]). The resulting **GW7604-Alk-PtCl**_**3**_ complexes (Alk = Prop, But, Pent, Hex) degraded in aqueous solution only by exchange of the chlorido ligands. For example, **GW7604-Pent-PtCl**_**3**_ coordinated the amino acid alanine in the cell culture medium, bound the isolated nucleotide 5′-GMP, and interacted with the DNA (empty plasmid pSport1). It accumulated in estrogen receptor (ER)-positive MCF-7 cells primarily *via* cytosolic vesicles, while it was only marginally taken up in ER-negative SKBr3 cells. Accordingly, **GW7604-Pent-PtCl**_**3**_ and related complexes were inactive in SKBr3 cells. **GW7604-Pent-PtCl**_**3**_ showed high affinity to ERα and ERβ without mediating agonistic or ER downregulating properties. **GW7604-Alk** ligands also increased the cyclooxygenase (COX)-2 inhibitory potency of the complexes. In contrast to Zeise’s salt, the **GW7604-Alk-PtCl**_**3**_ complexes inhibited COX-1 and COX-2 to the same extent.

## Introduction

**Chart 1:**
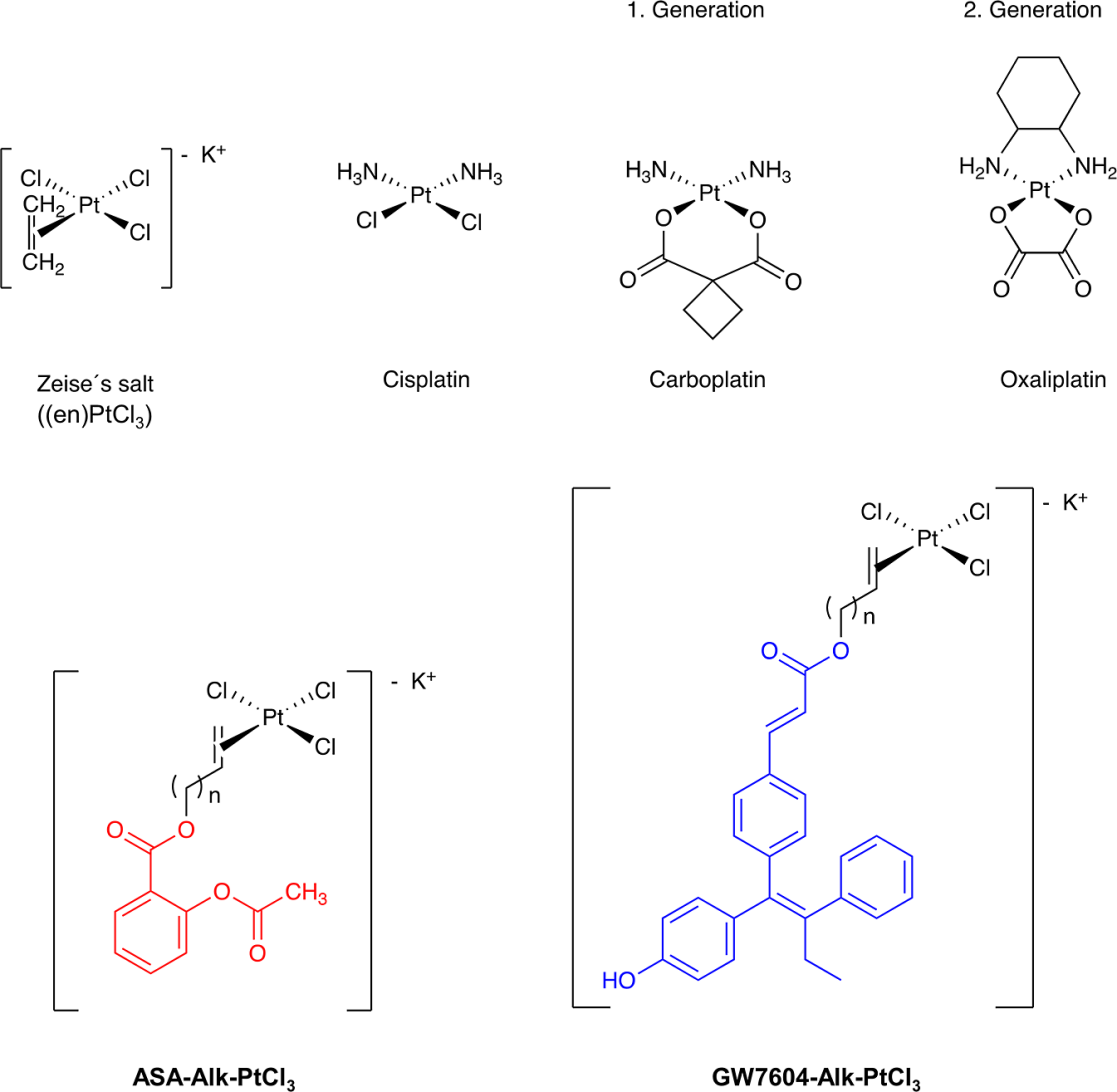
Platinum(II)-Based Drugs (Cisplatin, Carboplatin, Oxaliplatin) and Innovative New Complexes Derived From Zeise’s Salt [**ASA-Alk-PtCl**_**3**_ and **GW7604-Alk-PtCl**_**3**_; Alk = Prop (*n* = 1), But (*n* = 2), Pent (*n* = 3), Hex (*n* = 4)]. Acetylsalicylic Acid (**ASA**) is Indicated in Red, **GW7604** in Blue

In 1827, the Danish chemist William Christopher Zeise made a ground-breaking discovery while studying the reaction between platinum and ethylene. He observed the formation of a yellow precipitate, which he later identified as a complex with a trichloridoplatinate(II) (PtCl_3_) moiety bound to an ethylene molecule.^1−4^ This so-called Zeise’s salt (Chart 1) represents the first known organometallic compound.

Seventeen years later, the Italian chemist Michele Pyrone synthesized another famous platinum derivative, the *cis*-diamminedichloridoplatinum(II)^5,6^ (Cisplatin, Chart 1), whose exact structure was proposed by Alfred Werner in 1893.^7^ Further on, it was used as an excellent example for teaching coordination chemistry.

It took another 70 years before the biological activity of Cisplatin was discovered. In 1965, Barnett Rosenberg investigated the behavior of *Escherichia coli* bacteria in an electric field and observed the influence of Cisplatin on growth and shape of these microorganisms.^8,9^

In 1979, Cisplatin became the first platinum-based drug approved for cancer chemotherapy and remains one of the most widely used antitumor drugs in the treatment of, *e.g.,* testicular, ovarian, and lung cancer.^10−13^ However, its clinical use is restricted by significant side effects and the development of Cisplatin resistance in cancer cells.^14−18^

To overcome these limitations, Cisplatin was structurally modified to reduce toxicity to healthy cells and to improve selectivity toward cancer cells. Exchange of the chlorido leaving groups for a 1,1-cyclobutanedicarboxylate resulted in Carboplatin (Chart 1) with a more favorable toxicity profile. Oxaliplatin (Chart 1), the third platinum-based drug approved for tumor therapy in Europe, is a second-generation platinum(II) drug containing a 1,2-diaminocyclohexane (DACH) carrier ligand, which mediates high activity against colon carcinoma. Furthermore, the DACH ligand affects the ability of the cells to tolerate deoxyribonucleic acid (DNA) platinum adducts (circumvention of resistance) and the oxalate as a leaving group lowers the Cisplatin-like toxicity.^19,20^

The mechanism of action of most optimized platinum drugs still relies primarily on interaction with DNA, resulting in unselective activity and toxicity.^21,22^ Therefore, the search for new lead structures to design complexes with more tumor selectivity or with an alternative mode of action is ongoing.^23,24^

An attractive compound for optimization is Zeise’s salt, whose biological activity has been insufficiently investigated. In contrast to Cisplatin, only a limited number of publications deals with its biomacromolecule interactions.

However, we have already shown that Zeise’s salt is an effective inhibitor of the cyclooxygenase (COX) enzymes,^25^ which are involved in the regulation of inflammation and pain.^26,27^ The COX pathway also plays an important role in the development and progression of cancer as well as in the regulation of the tumor microenvironment.^28−31^ Especially the COX-2 isoenzyme is a very interesting target. When present in tumor cells, it oxidizes arachidonic acid to prostaglandin E2 (PGE2), which promotes, for example, the growth of hormone-dependent tumors.^32^

Unfortunately, Zeise’s salt effectively inhibits COX-1 (100%) in an assay at the isolated enzyme (incubation time 10 min), but it was inactive at COX-2 at a comparable concentration (10 μM).^33^

Moreover, Zeise’s salt showed no cytotoxicity against tumor cells *in vitro*. This could be the result of the formation of less strong DNA interactions compared to Cisplatin or the fast degradation in cell culture medium.^25^ In aqueous solutions, Zeise’s salt underwent aquation within minutes, followed by an internal redox reaction to acetaldehyde and platinum(0).^34,35^

The biological activity of Zeise’s salt can be optimized if it is linked to acetylsalicylic acid (**ASA**) *via* an alkyl spacer. As previously published, the resulting **ASA-Alk-PtCl**_**3**_ complexes (Chart 1) exhibited high COX-1 inhibitory activity. The influence on COX-2, however, was only slightly increased compared to Zeise’s salt.^33,36^

**ASA-Alk-PtCl**_**3**_ complexes were more stable than Zeise’s salt and interestingly degraded by a different route. Redox reactions were not observed in aqueous solutions. The main degradation pathway was the platinum-mediated ester cleavage of the benzoic acid ester. While **ASA-Prop-PtCl**_**3**_ decomposed within minutes (half-live τ_1/2_ = 35.7 min), elongation of the spacer to But, Pent, or Hex distinctly increased the half-life to hours (τ_1/2_ = 55.4–73.5 h).^33^

**ASA-Alk-PtCl**_**3**_ complexes showed higher cytotoxicity than the Zeise’s salt but no selectivity for tumor cells.

Therefore, in this structure–activity relationship study, **ASA** was exchanged for (*E/Z*)-3-(4-((*E*)-1-(4-hydroxyphenyl)-2-phenylbut-1-enyl)phenyl)acrylic acid (**GW7604** → **GW7604-Alk-PtCl**_**3**_), which represents an active metabolite of the selective estrogen receptor downregulator (SERD) Etacstil.^37−40^**GW7604** possesses high affinity to the ligand-binding site (LBS) of the estrogen receptor (ER), acts as a SERD, and can be easily esterified with an alkanol or alkenol. The latter allows coordination to PtCl_3_, forming the pharmacophoric (en)PtCl_3_ moiety (en = ethylene).

**GW7604** was already utilized as a carrier and linked with a pharmacologically active compound that binds to the surface of the ER outside the LBS to prevent coactivator binding.^41^ The relative binding affinity (RBA) of these hybrid compounds to the ER depended on the spacer length and the kind of coactivator binding site inhibitor.^42^

A comparable design was employed in the present study to deliver Zeise’s salt to hormone-dependent tumor cells.

There are already numerous reports in the literature on the linking of metal complexes with macromolecules or other drugs *via* various alkyl chains. In most cases, a spacer was used to increase the hydrophobicity.^43−46^ This study, however, aims to find a suitable spacer that minimizes the influence of the PtCl_3_ unit on the interaction of **GW7604** with its preferred target. It is thought that the selectivity of **GW7604-Alk-PtCl**_**3**_ for hormone-dependent tumor cells and their cellular uptake is mediated through binding of the **GW7604** moiety to the membrane-associated ER (mER).

After crossing the cell membrane and transferring into the nuclei, the (en)PtCl_3_ moiety can interact with nucleobases of the DNA. It is further assumed that **GW7604-Alk-PtCl**_**3**_ also inhibits the COX-1/2 enzymes through the PtCl_3_ moiety. Such multitarget compounds could be very promising antitumor drug candidates.

This paper describes the synthesis and characterization of **GW7604**-Zeise’s salt conjugates. The influence of the spacer length [(CH_2_)*_n_*; *n* = 1–4, Chart 1] on the stability of the **GW7604-Alk-PtCl**_**3**_ complexes and on their cytotoxicity and tumor selectivity was examined *in vitro* using hormone-dependent and independent breast cancer cell lines. The results will shed light on the potential of this new class of compounds for cancer therapy and point the way for future investigations in this promising field of research.

## Results and Discussion

### Synthesis

**Scheme 1:**
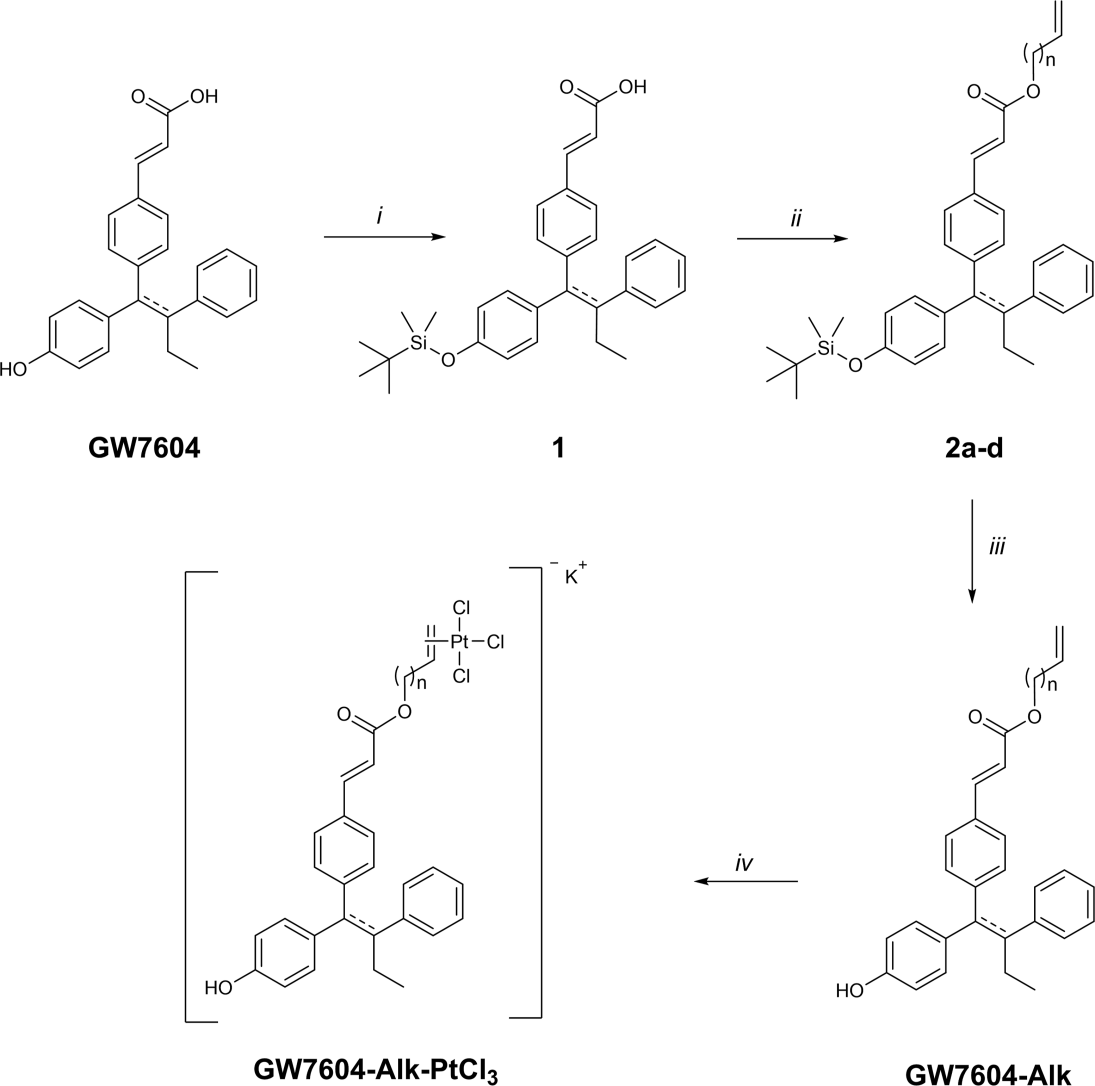
Synthesis of the **GW7604-Alk-PtCl**_**3**_ Complexes (Alk = Prop (*n* = 1), But (*n* = 2), Pent (*n* = 3), Hex (*n* = 4), Reagents and Conditions: (*i*) TBDMSCl, DMF, Imidazole, rt, 24 h; (*ii*) DMAP, Alkenol (*n* = 1–4), EDC, DCM, 0 °C to rt, 17 h, 55–100%; (*iii*) TBAF, THF, 0 °C to rt, 3 h, 55–93%; (*iv*) K[PtCl_3_(C_2_H_4_)], EtOH, 48–50 °C, 3 h, 33–56%

The synthesis of the **GW7604-Alk-PtCl**_**3**_ complexes was performed in a multistep procedure depicted in Scheme 1, starting from **GW7604**, which was prepared according to the literature.^41^

In the first step (*i*), the phenolic OH group of **GW7604** was protected with *tert*-butyldimethylsilyl chloride (TBDMSCl) to avoid an intermolecular reaction with the activated carboxylic acid during ester formation. Dimethylformamide (DMF) served as the catalyst, with imidazole as the auxiliary base. Furthermore, TBDMSCl had to be added in excess because the carboxyl group was silylated, too. Since such silyl acrylates are sensitive to hydrolysis, this unwanted byproduct could be hydrolyzed *in situ* by adding a 0.5 N K_2_CO_3_ solution dropwise to the reaction mixture.

The resulting compound **1** was then esterified in dichloromethane (DCM) with the respective alkenol (*n* = 1–4) by a modified Steglich-esterification using 4-(dimethylamino)pyridine (DMAP) as a base and 1-ethyl-3-(3-(dimethylamino)propyl)carbodiimide (EDC) as a coupling reagent (step *ii*).

Subsequent treatment of the formed compounds **2a**-**d** with tetra-*n*-butylammonium fluoride (TBAF) in tetrahydrofuran (THF) led to selective cleavage of the silyl ether (step *iii*). The alkenyl ester of **GW7604-Alk** was stable under the conditions used.

In the final step (step *iv*), **GW7604-Alk** was coordinated to platinum in an olefin-exchange reaction with Zeise’s salt (K[PtCl_3_(C_2_H_4_)]). In anhydrous, degassed ethanol (EtOH), the incoming olefin rapidly displaced the ethylene ligand, supported by the volatilization of ethylene at the used temperature of 48–50 °C. Upon addition of diethyl ether to the cooled solution, **GW7604-Alk-PtCl**_**3**_ precipitated as yellow solids.

### Spectroscopic Characterization

The **GW7604-Alk-PtCl**_**3**_ complexes were characterized by ^1^H, ^13^C, and ^195^Pt nuclear magnetic resonance (NMR) spectroscopy as well as ^1^H/^1^H homonuclear correlation spectroscopy (COSY) and ^1^H/^13^C heteronuclear single quantum coherence (HSQC) NMR experiments (Figures S1–S16). Figure 1 displays excerpts of the spectra of **GW7604-But** and **GW7604-But-PtCl**_**3**_. These compounds were chosen for NMR studies because their signals are well separated in the spectra and can be evaluated to confirm complexation and the presence of possible isomers.

**Figure 1:**
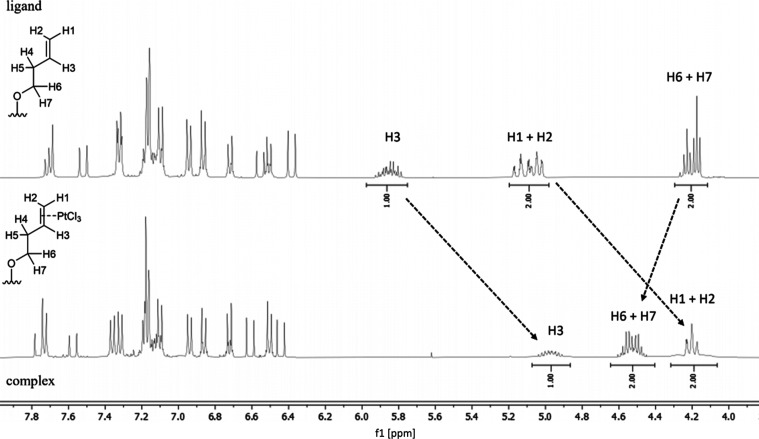
Excerpts of the ^1^H NMR (400 MHz) spectra of **GW7604-But** (top) and **GW7604-But-PtCl**_**3**_ (bottom) recorded in acetone-*d*_*6*_.

The spectrum of **GW7604-But** confirms the presence of two isomers, as indicated by a double set of signals in the region of the aromatic protons. The 1,1,2-triarylethylene unit adopts both an *E* and a *Z* configuration. Since the acrylic ester is exclusively *E*-configured (^3^*J*_H,H_ = 16.0 Hz), only two isomers (*E,E* and *E,Z*) are available.

Further signals at 5.00–5.21 ppm (C=C**H**_2_, H1 and H2), 5.76–5.90 ppm (C–C**H**=C, H3), 4.15–5.26 ppm (OC**H**_2_–C, H6 and H7), and additionally at 2.37–2.56 ppm (OCH_2_–C**H**_2_–C=, H4, H5, not shown in Figure 1) result from the but-3-en-1-yl chain.

Upon coordination, H1 to H3 are high-field shifted (H1 and H2: δ = 4.09–4.36; H3: δ = 4.84–5.14), while H6 and H7 are located at a lower field (δ = 4.44–4.64) (Figure 1).

**Figure 2:**
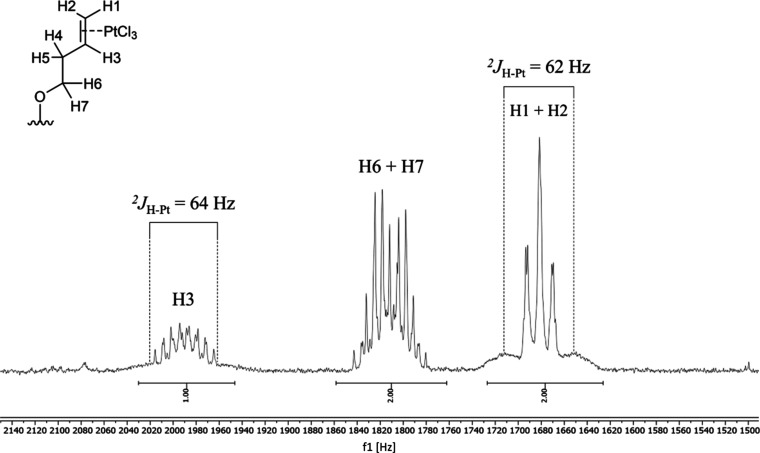
Excerpts of the ^1^H NMR spectrum (400 MHz) of **GW7604-But-PtCl**_**3**_ recorded in acetone-*d*_*6*_.

These data document a reduced binding order of the C=C moiety bound to platinum(II). Moreover, platinum satellites at H1/H2 and H3, resulting from *J*_Pt–H_ couplings of 62–64 Hz (Figure 2), confirm complexation.

It should be noted that the unsymmetrically substituted ethylene is prochiral and forms an asymmetric unit when bound to PtCl_3_. The *E/Z* configuration of the 1,1,2-triarylethylene unit leads to two diastereomeric pairs of enantiomers (isomers I/II and isomers III/IV, Figure S17). Consequently, the ^1^H NMR spectra of **GW7604-But-PtCl**_**3**_ also depict only two sets of resonances (Figure 1). The separation of the diastereomeric isomers was not successful because *E/Z* isomerization occurred again during isolation from organic solvents.

### Stability of GW7604-Alk-PtCl_3_ in Organic Solutions

Furthermore, it needs to be clarified whether organic solvent molecules are capable of ligand exchange reactions at the PtCl_3_ unit and form corresponding reaction/degradation products of the **GW7604-Alk-PtCl**_**3**_ complexes. Therefore, ^1^H NMR experiments were performed with noncoordinating [*e.g*., acetone-*d*_*6*_, methanol-*d*_*4*_] and coordinating [e.g., acetonitrile-*d*_*3*_ , DMF-*d*_*7*_, dimethyl sulfoxide-*d*_*6*_ (DMSO-*d_6_*)] solvents. It should further be assessed whether the solvents are suitable for preparing stock solutions for structural characterization or *in vitro* testing.

The solutions (concentrations of 4.5–5.0 mM) were stored in NMR tubes at room temperature (rt) after being purged with argon.

**GW7604-Alk-PtCl**_**3**_ complexes dissolved in acetone-*d*_6_ (Figure S18) or methanol-*d*_4_ (Figure S19) did not change their signal pattern in the spectra during 1 week of incubation. Therefore, it can be concluded that these solvents do not affect the stability of the complexes and they are suitable for structural characterization.

For the experiments with coordinating solvents, **GW7604-Pent-PtCl**_**3**_ was chosen because it was used for extended *in vitro* experiments.

A solution in acetonitrile-*d*_3_ was monitored over a period of 23 days (Figure S20). After 4 days, about 25% of the complex released the ligand **GW7604-Pent**, as indicated by the occurrence of the free ligand-specific signals for the -OC**H**_2_-, the terminal =C**H**_2_, and the -C**H=** protons at δ = 4.15, 4.95–5.10, and 5.79–6.92, respectively. Their integral increased to about 35% after 9 days. Complete degradation of the complex took place within 23 days. Interestingly, only the release of the ligand was observed. The ester remained intact and no redox reaction occurred. This finding is in contrast to the results obtained with **ASA-Alk-PtCl**_**3**_ complexes, which decomposed upon cleavage of the benzoic acid ester.^33^

**GW7604-Pent-PtCl**_**3**_ degraded in DMF-*d*_*7*_ with the same kinetics as in acetonitrile-*d*_*3*_. The detected amount of free ligand was about 33% after 9 days and about 40% after 14 days of incubation (Figure S21).

Finally, **GW7604-Pent-PtCl**_**3**_ was dissolved in DMSO-*d*_6_. Since it is well-known that the solvent molecules interact rapidly with platinum(II) complexes in ligand exchange reactions,^47,48^ the first measurement was performed after just 5 min.

The spectrum taken in DMSO-*d*_6_ (Figure S22) contained only signals of the free **GW7604-Pent** ligand, indicating a DMSO-induced cleavage of the alkene-platinum(II) bond. The formed platinum(II) adduct was identified as [PtCl_3_(DMSO)]^−^ by its ^195^Pt NMR signal at −2953 ppm (Figure S23).^49^

The stability in DMSO was also investigated for Zeise’s salt. Immediately after dissolution in DMSO-*d*_6_, gas bubbling occurred from the released ethylene. Again, [PtCl_3_(DMSO)]^−^ was formed, identified by its ^195^Pt NMR spectrum (δ = −2953 ppm).

This finding demonstrates another reaction mode of Zeise’s salt derivatives compared to Cisplatin. After DMSO attack at the platinum(II), a trigonal pyramidal transition state is formed, from which the ethylene is liberated. In case of Cisplatin, chloride is the leaving group, and the NH_3_ ligands remain at the metal center (→ [(NH_3_)_2_PtCl(DMSO)]^+^).

These results clearly show that DMF can be used as a solvent for *in vitro* studies. However, the stock solutions must be freshly prepared and cannot be stored at rt. For spectroscopic characterization, DMF-*d*_*7*_, acetone-*d*_*6*_, and methanol-*d*_*4*_ are suitable. DMSO-*d*_6_ must be avoided as it reacts too fast with the platinum(II) complexes.

### HPLC Analysis of GW7604-Alk-PtCl_3_ Complexes

#### Determination of **GW7604-Alk-PtCl**_**3**_ Purity

The purity of the compounds was analyzed by high-performance liquid chromatography (HPLC) using an RP-C18 column and an isocratic elution of acetonitrile (ACN)/water [90/10 (*v*/*v*)].

**Figure 3:**
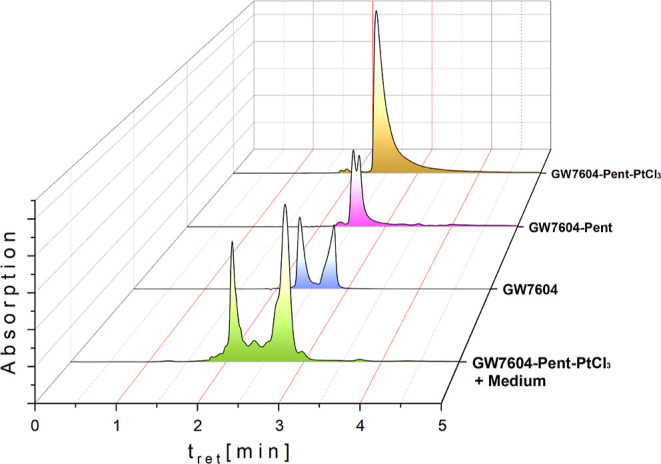
HPLC chromatograms of **GW7604**, **GW7604-Pent**, and **GW7604-Pent-PtCl**_**3**_ (analysis of stock solutions and the methanolic extract from medium (DMEM + 10% FCS); concentration 30 μM; incubation time 4 h at 37 °C). All compounds were analyzed as solutions in methanol.

Figure 3 depicts the chromatograms of **GW7604**, **GW7604-Pent**, and **GW7604-Pent-PtCl**_**3**_. As already determined by ^1^H NMR spectroscopy, **GW7604** existed as a mixture of *E/Z* isomers with retention times (*t*_R_) of 2.33 and 2.81 min. The retention times of the isomers converge upon esterification (**GW7604-Pent**: *t*_R_ = 2.51 and 2.61 min) and are identical after binding of **GW7604-Pent** to platinum(II) (**GW7604-Pent-PtCl**_**3**_: *t*_R_ = 2.41 min). The used HPLC method thus did not allow the separation of the complex isomers but the discrimination from the ligands and **GW7604**.

Analysis of all complexes indicated a purity of >95% (Figures S24–S27).

#### Determination of **GW7604-Alk-PtCl_3_** Stability in Cell Culture Medium

Another question that needs to be clarified is the possible degradation of the **GW7604**-based platinum(II) compounds in cell culture medium.

As an example, **GW7604-Pent-PtCl**_**3**_ (concentration 30 μM) was dissolved in Dulbecco’s modified Eagle’s medium [DMEM supplemented with 10% fetal calf serum (FCS)] and incubated for 4 h at 37 °C in the dark. After protein precipitation with ice-cold methanol (MeOH), the compounds remaining in solution were analyzed by HPLC and electrospray ionization high-resolution mass spectrometry (ESI-HR-MS).

The HPLC chromatogram (Figure 3) documents a complete degradation within 4 h. However, the two main peaks occurring neither correspond to the parent complex **GW7604-Pent-PtCl**_**3**_ nor to the free ligand **GW7604-Pent**, or **GW7604**.

**Figure 4:**
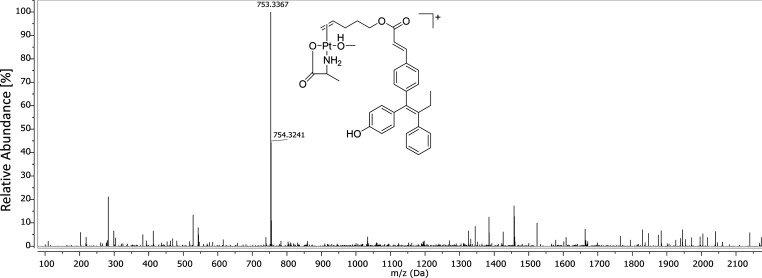
Full ESI-HR-MS (positive mode) spectrum of the methanolic extract from DMEM (+10% FCS) incubated with **GW7604-Pent-PtCl**_**3**_.

Analysis of the methanolic extract with ESI-HR-MS in the positive mode showed the presence of a major species (Figure 4), which bears **GW7604-Pent**, the amino acid alanine (Ala), and MeOH as ligands.

This peak shows a clear platinum isotopic pattern, which documents the presence of a platinum moiety. The isotopic distribution and the related simulation are depicted in Figure S28. Therefore, the main degradation product in cell culture medium can be unequivocally assigned to the **[GW7604-Pent-Pt(Ala)(CH**_**3**_**OH)]**^**+**^ complex. The presence of negatively charged complexes can be excluded because the spectrum obtained in the negative mode included no further signals of platinum species.

This finding is very surprising because the medium does not only contain alanine. It seems that other amino acid derivatives were not formed. However, it is very likely that **GW7604-Pent-PtCl**_**3**_ reacted with proteins in the medium, which were precipitated upon addition of MeOH. It should be noted that MeOH was used for precipitation, as most of the leaving group-modified Zeise’s salt derivatives proved to be soluble in this solvent.

The reaction of Zeise’s salt with alanine was already published by Belluco et al.^50^ They described the mechanism and the kinetics of the binding to [PtCl_3_(C_2_H_4_)]^−^. From this study, it can be deduced that alanine as an ingredient of DMEM attacked with its amino group in a first step the (en)PtCl_3_ moiety of **GW7604-Pent-PtCl**_**3**_ and displaced the chlorido ligand *trans* to the ethylene. In the second step, a five membered chelate ring was formed by the carboxylic group, which liberated a second chloride.

The HPLC and ESI-HR-MS studies demonstrated that the Zeise’s salt moiety retained a reactive position at the platinum(II) after binding of an amino acid and is therefore suitable for coordination to bionucleophiles such as proteins or DNA. This contrasts with Cisplatin, which loses its DNA binding tendency upon coordination to amino acids.^51^

### Intracellular Accumulation Studies

In order to reach the DNA, the complexes must first pass through the cell membrane and penetrate into the nuclei. Therefore, the cellular uptake kinetics of **GW7604-Alk-PtCl**_**3**_ complexes were studied using the example of **GW7604-Pent-PtCl**_**3**_. This complex and Cisplatin, respectively, were incubated at a concentration of 20 μM with MCF-7 or SKBr3 cells. After various time points [i.e., 0 (3 min), 0.5, 1, 2, 4, and 8 h], cell pellets were prepared, and the platinum content was quantified by high-resolution continuum source atomic absorption spectrometry (HR-CS-AAS).^52^

**Figure 5:**
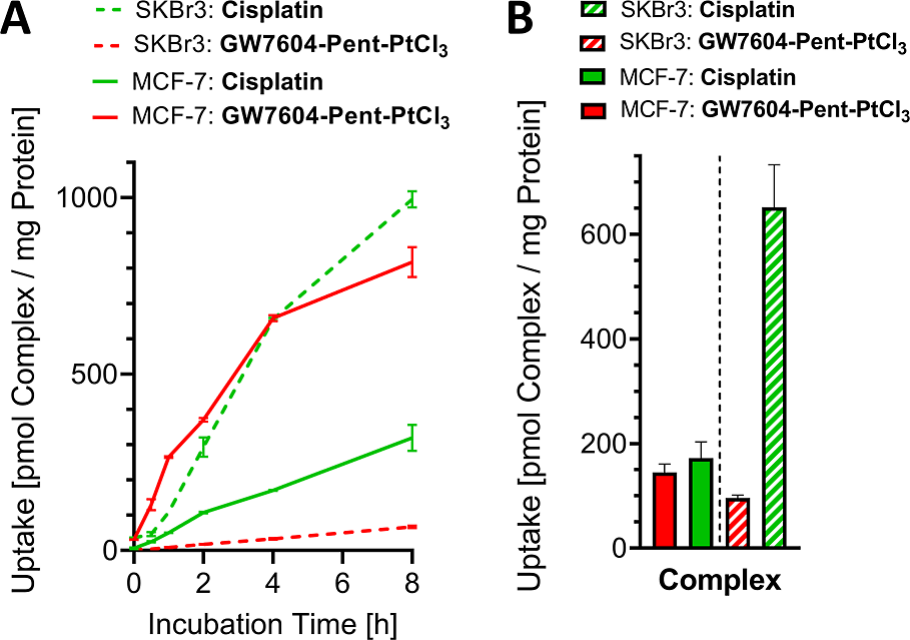
(A) Time-dependent uptake of **GW7604-Pent-PtCl**_**3**_ and Cisplatin into MCF-7 and SKBr3 cells. (B) Uptake into the nuclei of MCF-7 and SKBr3 after 24 h. Values were obtained by measuring platinum with HR-CS-AAS and representing the mean ± SD/+ SD of 2 independent experiments. Complex concentration: 20 μM.

Figure 5A depicts the intracellular platinum concentration expressed as pmol complex per mg cellular protein.

**GW7604-Pent-PtCl**_**3**_ caused in MCF-7 cells platinum amounts of 130 pmol/mg after 0.5 h, 265 pmol/mg after 1 h, 371 pmol/mg after 2 h, 659 pmol/mg after 4 h, and 818 pmol/mg after 8 h. The maximum intracellular platinum concentration was not reached.

Cisplatin showed nearly linear uptake kinetics with 319 pmol/mg after 8 h. These data document a rapid and approximately 3-fold higher accumulation rate of **GW7604-Pent-PtCl**_**3**_ compared to Cisplatin and confirm the suitability of **GW7604-Pent** as a carrier ligand that determines the passage of the complex through the membrane of MCF-7 cells.

The transfer of anticancer compounds into tumor cells is essential for their biological effect and can occur by diffusion (passive transport) or by a membrane-bound receptor/transporter.^53,54^ It is well-known that mainly the nonleaving groups are responsible for the transport of platinum complexes.

Cisplatin bearing two NH_3_ ligands accumulated slowly in the cells without reaching saturation (Figure 5A).^55^ The most important mechanisms of Cisplatin uptake are passive diffusion and active transport by organic cation transporters, such as the copper transporter Ctr1.^56^

Zeise’s salt containing the η^2^-bound ethylene is taken up into the cells with the same kinetics as Cisplatin.^25^ However, the exact mechanism is still unknown.

Exchange of ethylene for the **GW7604-Pent** ligand strongly increased the intracellular platinum level. This may be the consequence of the higher lipophilicity and thus improved passive transport or, more likely, binding to the mER followed by vesicular internalization.

It was already confirmed that hormone binding to membrane receptors activates intracellular signal cascades and induces internalization. Morphological studies in MCF-7 cells revealed that the membrane-bound type of classical ERα is internalized after ligand binding *via* dynamin-dependent, caveolae-mediated endocytosis.^57^

**Figure 6:**
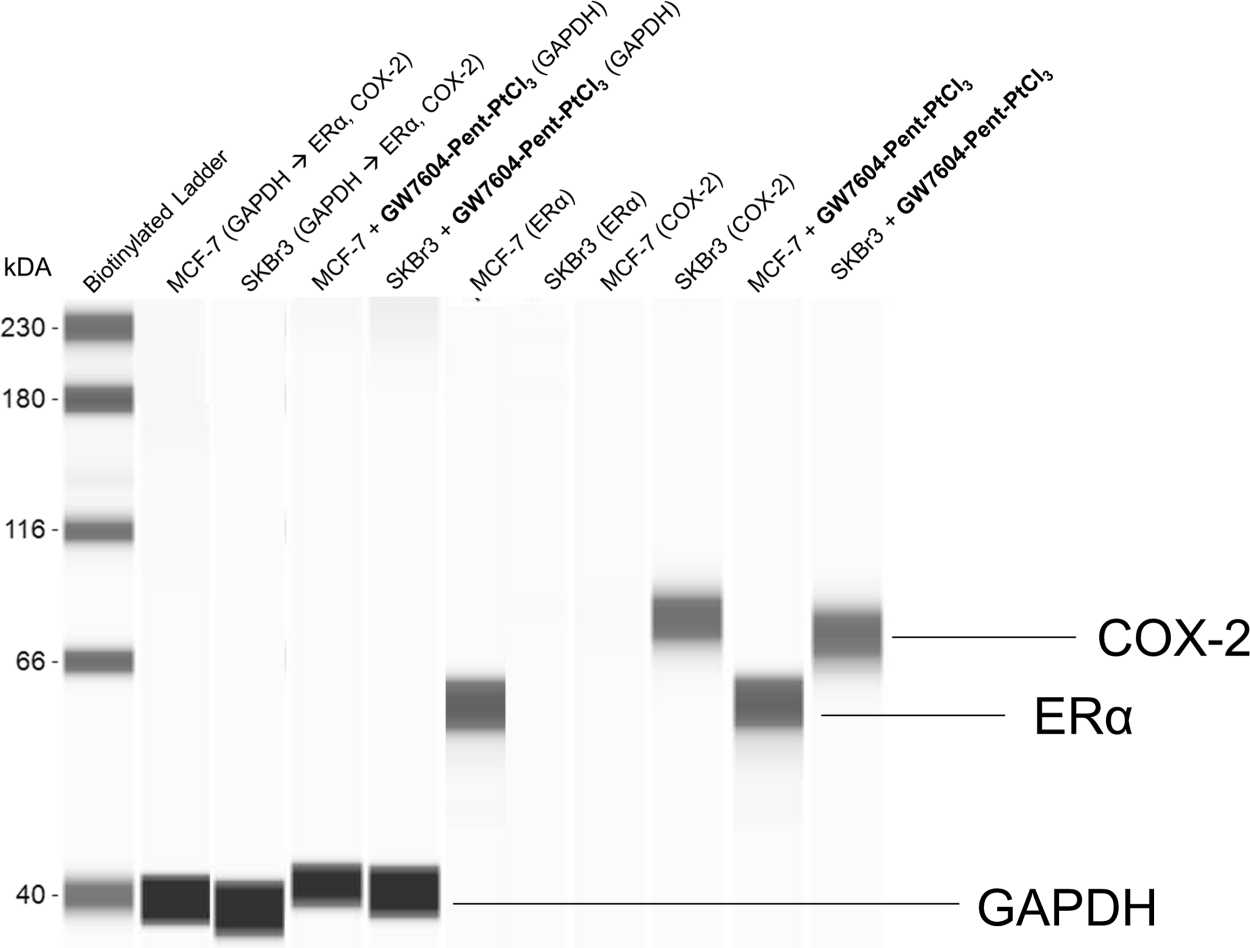
Determination of the baseline estrogen receptor alpha (ERα) and cyclooxygenase 2 (COX-2) enzyme expression in MCF-7 and SKBr3 cells as well as influence of **GW7604-Pent-PtCl**_**3**_ on ERα and COX-2 downregulation. Western blot analysis was performed using a fully automated capillary Western blotting system. Complex concentration: 20 μM.

Participation of the ER in the uptake mechanism of **GW7604-Pent-PtCl**_**3**_ was confirmed by the results obtained with SKBr3 cells. MCF-7 cells expressed ERα, while SKBr3 cells contained neither ERα nor ERβ, as confirmed by Western blot analysis (Figure 6).

**GW7604-Pent-PtCl**_**3**_ showed linear uptake kinetics into SKBr3 cells with a platinum amount of only 67 pmol/mg after 8 h, about one-twelfth of that found in MCF-7 cells. In contrast, Cisplatin is 15-fold higher accumulated (995 pmol/mg) in SKBr3 than in MCF-7 cells after 8 h.

**Figure 7:**
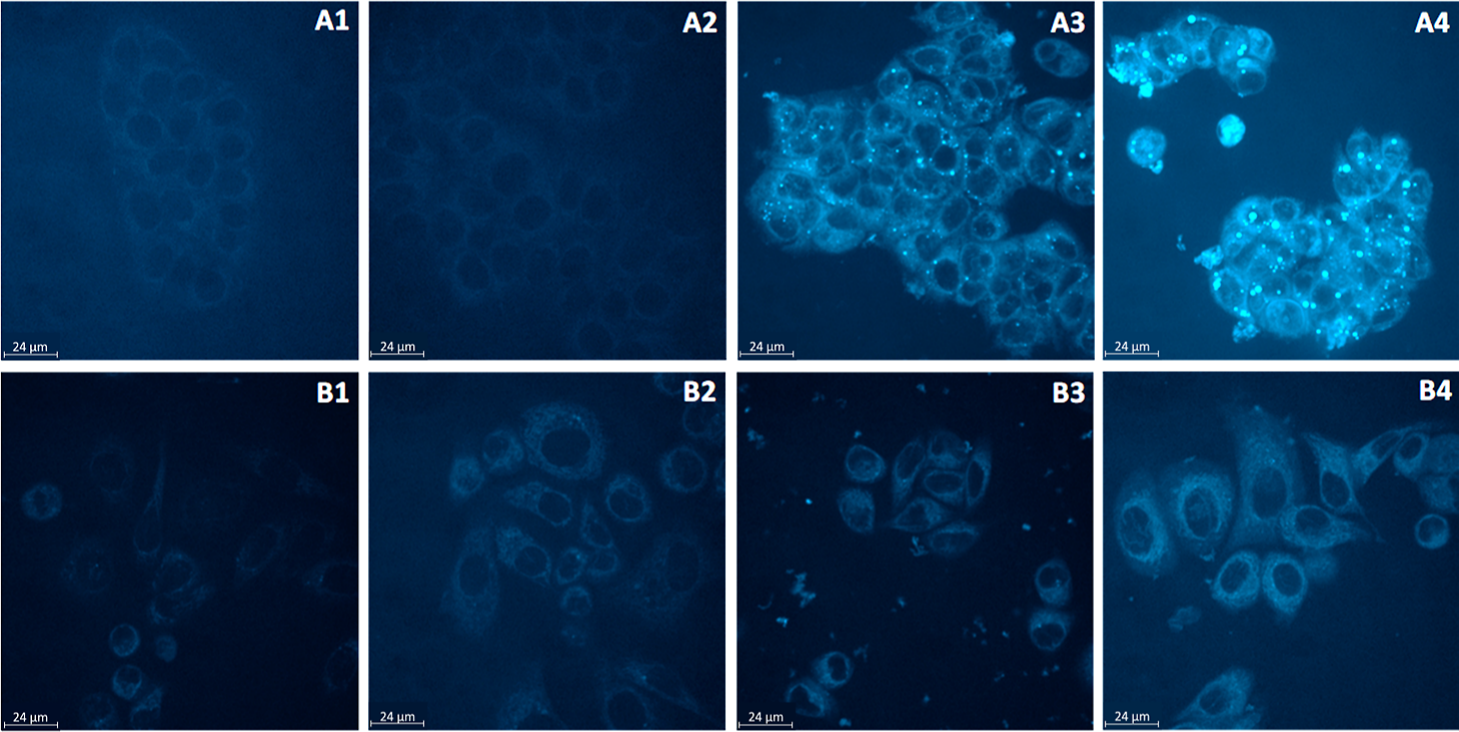
Evaluation of the cellular accumulation *via* real-time live confocal microscopy. Top row: Hormone-dependent MCF-7 cells incubated with (A1) DMF; (A2) **GW7604-Pent**; (A3) **GW7604-Pent-PtCl**_**3**_ for 24 h; (A4) **GW7604-Pent-PtCl**_**3**_ for 48 h. Bottom row: Hormone-independent SKBr3 cells incubated with (B1) DMF; (B2) **GW7604-Pent**; (B3) **GW7604-Pent-PtCl**_**3**_ for 24 h; (B4) **GW7604-Pent-PtCl**_**3**_ for 48 h. Compound concentration: 20 μM.

To obtain further information about membrane transport, MCF-7 and SKBr3 cells were incubated with **GW7604-Pent-PtCl**_**3**_ or the unbound ligand **GW7604-Pent** and examined by real-time live confocal microscopy using an inverted microscope in arrangement with a spinning disc confocal system (Figure 7).

As depicted in Figure 7A2 (MCF-7 cells) and Figure 7B2 (SKBr3 cells), the ligand **GW7604-Pent** did not cause compound-dependent intracellular fluorescence.

In contrast, fluorescent vesicles were detected in **GW7604-Pent-PtCl**_**3**_-treated MCF-7 cells after 4 h (image not shown), the number of which significantly increased during the incubation periods of 24 h (Figure 7A3) and 48 h (Figure 7A4). Incubation for up to 72 h led to a partial collapse of the vesicles (picture not shown) and release of the complex.

The fluorescence of the vesicles resulted from the fluorescence behavior of the platinum complexes. The accumulation in the vesicles reached the critical concentration that enables detection by fluorescence microscopy. In agreement with the uptake studies, only extremely low fluorescence was observed with **GW7604-Pent-PtCl**_**3**_ in SKBr3 cells, without formation of vesicular structures (Figure 7B3/B4).

A closer analysis of Figure 7A4 indicates in MCF-7 cells the presence of vesicles with diameters greater than 1 μm, most of which are 2.80 ± 0.89 μm. These values (>1 μm) allow their classification as giant vesicles (GVs), which are part of the ERα-dependent trafficking system in MCF-7 cells, regulated by estradiol (E2) or other agonists.^58^ The lack of vesicle formation and the low intracellular platinum concentration in SKBr3 cells could therefore be the consequences of missing ERα expression.

Figure 7A3 further reveals that the GVs are located only in the cytosol and on the surface of the nuclei in MCF-7 cells. Fluorescence in the nuclei is not detectable. Therefore, the nuclei were analyzed for their platinum content using HR-CS-AAS.^25^

MCF-7 cells incubated with **GW7604-Pent-PtCl**_**3**_ at a concentration of 20 μM for 24 h contained 145 pmol complex/mg protein in the nuclei, very similar to that of Cisplatin (172 pmol/mg, Figure 5B).

In contrast, the nuclei of SKBr3 cells treated with **GW7604-Pent-PtCl**_**3**_ showed significantly lower platinum levels (96 pmol/mg), while Cisplatin caused a high platinum content of 652 pmol/mg.

### Determination of ERα and ERß Binding

To confirm ER binding, **GW7604-Pent** and **GW7604-Pent-PtCl**_**3**_ were studied in a time-resolved fluorescence energy transfer (TR-FRET) assay with the isolated ligand-binding domains (LBDs) of ERα and ERβ. It is well-documented that the 1,1,2-triarylbut-3-ene moiety of **GW7604** competes with E2 for the LBS, which can be expressed as relative binding affinity [RBA(E2) = 100%].^37,41,59^

**Figure 8:**
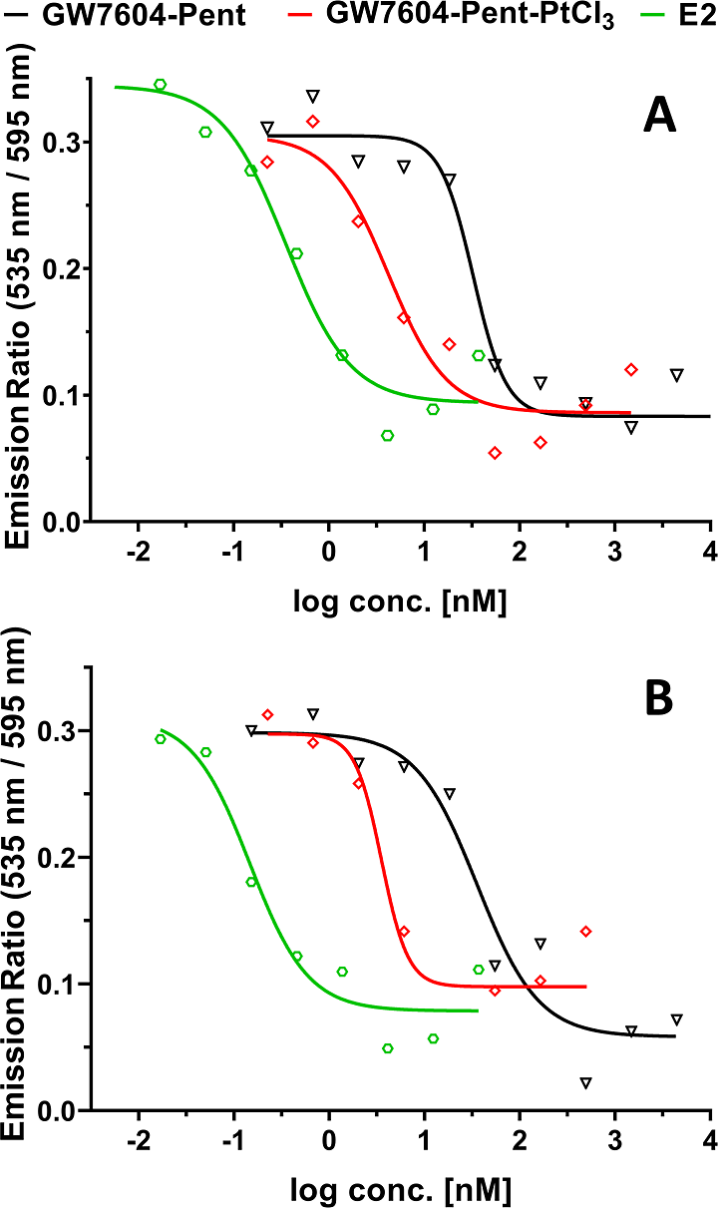
ER binding affinities of E2, **GW7604-Pent**, and **GW7604-Pent-PtCl**_**3**_ to the ligand-binding domain of (A) ERα and (B) ERβ. Data are given as mean of ≥3 independent experiments. Error bars are omitted to ensure better readability.

The graphs displayed in Figure 8 show the displacement of E2 from the LBS of ERα [IC_50_ (E2) = 0.35 nM] and ERβ [IC_50_ (E2) = 0.24 nM] by **GW7604-Pent** with IC_50_ (ERα) = 39.10 nM (RBA = 0.90%) and IC_50_ (ERβ) = 104.80 nM (RBA = 0.23%). This low affinity to ERα might be the reason for the absent vesicular accumulation in MCF-7 cells.

Interestingly, coordination of **GW7604-Pent** to PtCl_3_ strongly increased the receptor binding [**GW7604-Pent-PtCl**_**3**_: IC_50_ (ERα) = 4.81 nM (RBA = 7.29%); IC_50_ (ERβ) = 3.99 nM (RBA = 6.02%)], thus enabling the formation of GVs.

### Determination of DNA Interactions

The platinum amount in the nuclei of MCF-7 cells raised the question of whether **GW7604-Pent-PtCl**_**3**_ can bind to the DNA in the nuclei like Cisplatin.

**Figure 9:**
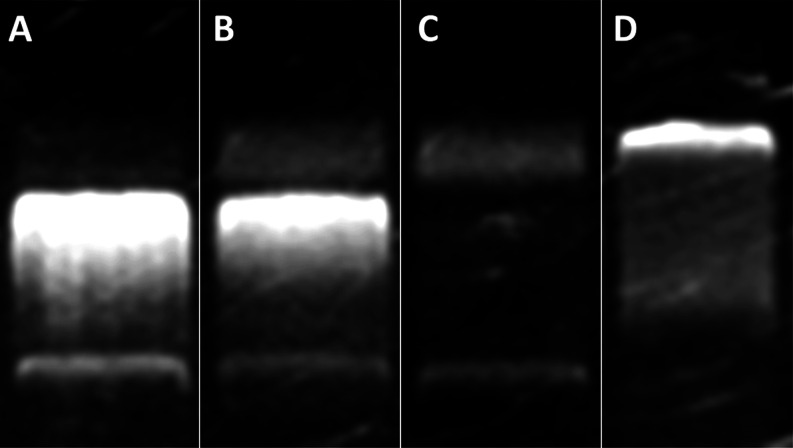
Interference with plasmid DNA. Compounds were incubated for 4 h with double-stranded plasmid DNA (pSport1) at 37 °C, followed by electrophoresis for 90 min. (A) Untreated control; (B) DMF; (C) Cisplatin, 15 μM; (D) **GW7604-Pent-PtCl**_**3**_, 20 μM.

Therefore, the general possibility of DNA interaction was examined by incubating the complex with the empty plasmid pSport1 at a concentration of 20 μM for 4 h at 37 °C. The gel electrophoretic separation^60^ (Figure 9) clearly indicates that **GW7604-Pent-PtCl**_**3**_ (Figure 9D) binds to DNA. The ladder structure differs from both the untreated sample (Figure 9A) and the solvent control (Figure 9B) and is similar to that of Cisplatin (Figure 9C).

**Figure 10:**
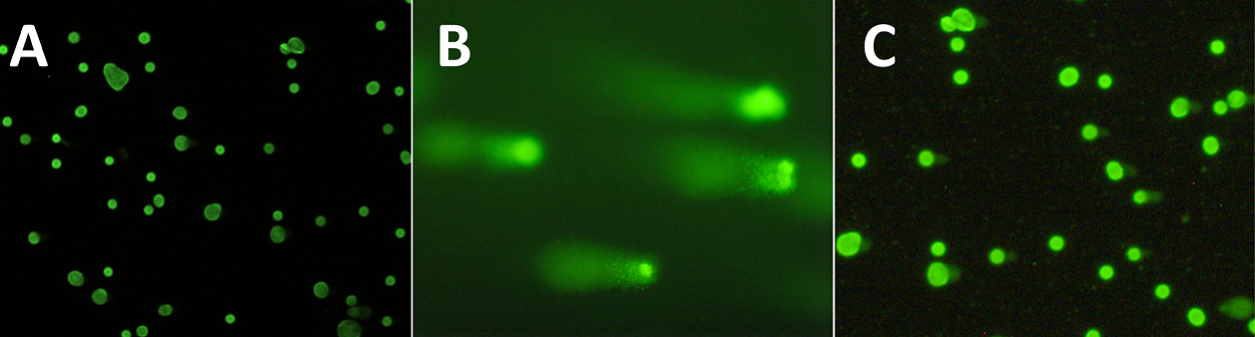
Fluorescence microscopic imaging as part of the comet assay using MCF-7 cells after 48 h of incubation with (A) DMF; (B) Cisplatin, 15 μM; and (C) **GW7604-Pent-PtCl**_**3**_, 20 μM.

The comet assay (Figure 10), however, documents different consequences of DNA binding. Cisplatin-induced intrastrand cross-links in MCF-7 cells (15 μM) after incubation for 48 h are visible as comet tails in the fluorescence micrograph (Figure 10B). In contrast, **GW7604-Pent-PtCl**_**3**_ did not show this effect even at higher concentrations (20 μM). The picture (Figure 10C) is very similar to that of the solvent control (Figure 10A).

A possible explanation could be that **GW7604-Pent-PtCl**_**3**_ formed only monofunctional DNA adducts. The complex contains, besides the **GW7604-Pent** moiety, three chlorido ligands as potential leaving groups. The degradation studies (see above) in DMEM (+10% FCS) already demonstrated that bionucleophiles, *e*.*g.*, amino acids (alanine), replace two chlorides prior to cell accumulation. One chloride remains for exchange by other nucleophiles or monofunctional binding to the DNA. Formations of intra- or interstrand cross-links are not very likely.

To get more information about the coordination of metal complexes to the DNA, the reaction with the nucleotide 5′-guanosine monophosphate (5′-GMP) is a commonly used model.^60−62^

Thereto, **GW7604-Pent-PtCl**_**3**_ was incubated in a 1:1 ratio with 5′-GMP in a MeOH/water mixture [80/20, (*v*/*v*)], and the reaction products were identified by ESI-HR-MS after 5 min and 24 h.

**Figure 11:**
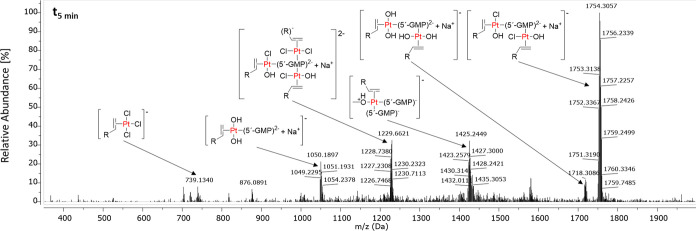
ESI-HR-MS spectrum (negative mode), obtained from a mixture of **GW7604-Pent-PtCl**_**3**_ and 5′-GMP in MeOH/water [80/20, (*v*/*v*)] after 5 min; R–CH=CH_2_: **GW7604-Pent**. Complex concentration: 100 μM.

Already after 5 min, various peaks corresponding to 5′-GMP-platinum adducts were present in the ESI-HR-MS spectrum (negative mode, Figure 11). Besides the initial complex (*m*/*z* 793; **GW7604-Pent-PtCl**_**3**_), the 5′-GMP monoadduct at *m*/*z* 1050 is shown.

Interestingly, the bound 5′-GMP is able to coordinate several **GW7604-Pent-PtCl**_**3**_ molecules in a 1:2 or 1:3 ratio. The most abundant peak (*m*/*z* 1754) could be assigned to a binuclear platinum species featuring one 5′-GMP molecule. Interestingly, all of these species underwent Cl/OH exchange to different extents.

This finding suggests that in the first reaction step, 5′-GMP replaces a chlorido ligand at **GW7604-Pent-PtCl**_**3**_. The remaining leaving groups are then exchanged for water (Cl/H_2_O exchange), followed by deprotonation due to the high acidity of the bound H_2_O molecules.

This hypothesis is confirmed by the spectrum recorded after 24 h (see Figure S29). The intensity of the peak at *m*/*z* 1753 strongly decreased in favor of the peak corresponding to the completely hydroxylated counterpart (*m*/*z* 1718).

The ESI-HR-MS spectrum further exhibits the peak of the species **[GW7604-Pent-PtCl(5′-GMP)**_**2**_**]**^**–**^ at *m*/*z* 1425. This documents that the Zeise’s salt derivative is generally able to form both mono- and bifunctional DNA adducts. The comet assay, however, excludes intra- or interstrand cross-links, which makes the binding of amino acids prior to coordination to the DNA very likely.

### Determination of Hormonal Activity

Besides DNA binding, interference with further intracellular pathways can participate in the mode of action.

As **GW7604-Pent-PtCl**_**3**_ represents a derivative of the SERD **GW7604**, it is of interest to examine whether the complexes exert hormonal activity. After the collapse of the GVs, the released complex can bind to the ER located in the cytosol, inducing hormonal intracellular effects.

However, both agonistic properties and SERD effects can be excluded. The complex neither stimulated the growth of MCF-7 cells at low concentrations (<100 nM, data not shown) nor downregulated the ER content (Figure 6).

### Inhibition of COX Isoenzymes

Another pathway in which the complexes can interfere is the arachidonic-prostaglandin cascade. Especially PGE2 is an important mediator in cell proliferation. PGE2 supports tumor growth by promoting angiogenesis, stimulating tumor-cell proliferation, and protecting tumor cells from apoptosis. In addition, PGE2 overexpression in tumors can lead to poor outcomes for patients.^32^

A possibility to block the growth of tumor cells is thus the inhibition of the COX-1/2 enzymes. While COX-1 is permanently expressed in various tumor cells, the inducible COX-2 isoform is upregulated, *e.g.*, by tumor promoters. Accordingly, a high concentration of COX-2 also leads to an increased conversion of arachidonic acid to PGE2 and thus to the stimulation of cell proliferation.^63^ This isoenzyme is therefore a suitable target for reducing the growth of tumor cells that overexpress the PGE2 produced by COX-2.

COX-1/2 inhibition was evaluated in an established assay on isolated isoenzymes. After incubation with the drugs (concentration: 20 μM) for 10 min, the remaining COX activity was measured by an enzyme-linked immunosorbent assay (ELISA).

**Figure 12:**
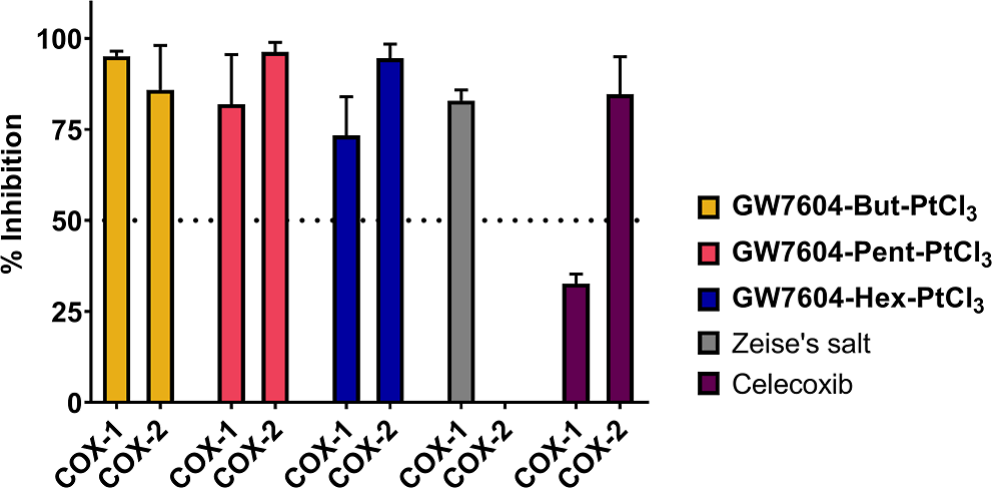
Inhibition of isolated human recombinant COX-1 and COX-2. Values are given as % COX inhibition calculated from the initial activity of vehicle-treated enzymes and represent the mean + SEM of ≥3 independent experiments. Concentrations: complexes 20 μM; Celecoxib 5 μM. Incubation time: 10 min.

**Figure 13:**
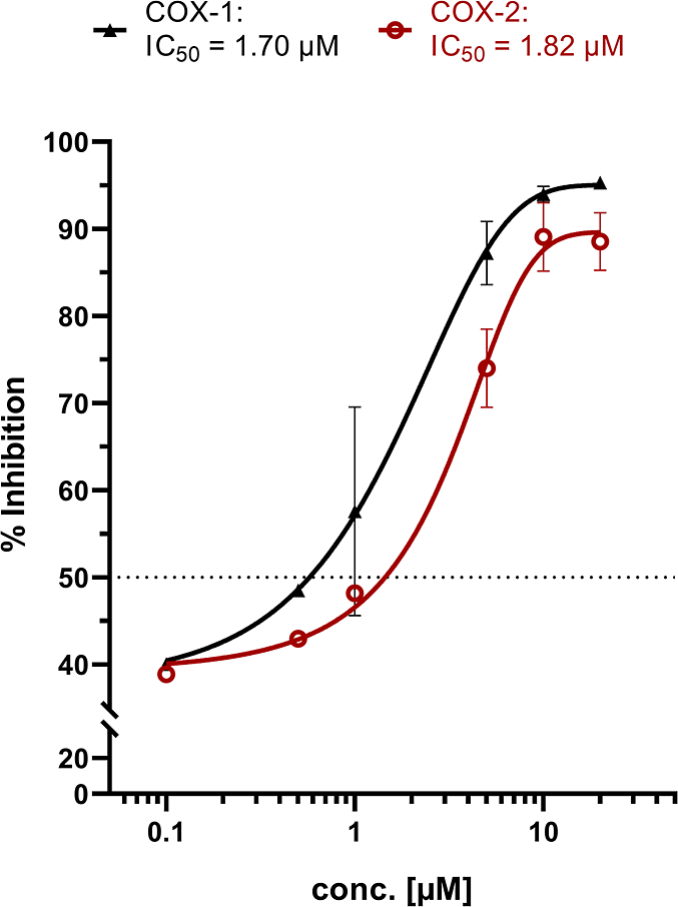
Concentration–response curves for COX-1 (black) and COX-2 (red) inhibition of **GW7604-Pent-PtCl**_**3**_. Values are given as *%* COX inhibition calculated from the initial activity of vehicle-treated enzymes and represent the mean ± SEM of 3 independent experiments. Complex concentration: 20 μM. Incubation time: 10 min.

Celecoxib (5 μM), used as a COX-2-selective reference, almost completely suppressed the COX-2 activity (Figure 12). The inhibition of COX-1 (30%) did not exceed the basal inactivation observed in this assay (40%, see Figure 13). As expected from prior studies, Zeise’s salt showed high COX-1 selectivity (inhibition COX-1: 83%; COX-2: 0%, Figure 12).

Optimizing the interference with the COX cascade by linking Zeise’s salt to **ASA** was already performed. However, the resulting **ASA-Alk-PtCl**_**3**_ complexes (Chart 1) had only slightly higher COX-2 inhibitory activity than Zeise’s salt.^33,36^

It was also shown that the organometallic substructure determines the efficacy of COX inhibitors. For example, binding to a dicobalt hexacarbonyl cluster strongly enhanced the COX inhibitory effect of **ASA**-propargyl ligands.^36,64−68^ Therefore, it was of interest to evaluate whether the **GW7604-Alk-PtCl**_**3**_ complexes influence the COX-1/2 inhibition, too.

**GW7604-Prop-PtCl**_**3**_ was excluded from testing because this complex does not have sufficient water solubility. The other **GW7604-Alk-PtCl**_**3**_ complexes completely inactivated both isoenzymes (Figure 12) at 20 μM, even though the ligands were derived from the SERD **GW7604** and not from the nonsteroidal anti-inflammatory drug **ASA**.

On the example of **GW7604-Pent-PtCl**_**3**_, the exact inhibitory potency was determined as a function of concentration. From Figure 13, it is visible that the concentration–response curves started with a 40% inhibition of COX-1 and COX-2. Therefore, only compounds causing effects above these values can be considered as active.

Furthermore, **GW7604-Pent-PtCl**_**3**_ reached the maximum activity on both isoenzymes at 10 μM. The calculated IC_50_ values are 1.82 μM (COX-2) and 1.70 μM (COX-1), which indicates a significantly increased COX-2 inhibition compared to Zeise’s salt. Since the **GW7604-Alk** ligands were completely inactive (data not shown), it can be stated that the (en)PtCl_3_ moiety is responsible for the interference with the catalytic oxidation of arachidonic acid, reducing the formation of PGE2.

The **GW7604-Alk** ligands are therefore not only carrier ligands for cellular uptake but also mediate the enhanced effect on the isolated COX-2 enzyme.

### Effects against Tumor Cell Lines

The **GW7604-Alk-PtCl**_**3**_ complexes (with exclusion of **GW7604-Prop-PtCl**_**3**_) were investigated for cytotoxic effects in SKBr3 (ER-negative, COX-2-positive, Figure 6), MCF-7 (ERα-positive, COX-negative, Figure 6), and noncancerous TSA-201 (human embryonal kidney) cells at concentrations of 10–30 μM using an established MTT (3-(4,5-dimethylthiazol-2-yl)-2,5-diphenyltetrazolium bromide) assay.^60,69^

After incubation for 72 h, the amounts of surviving, metabolically active cells were quantified based on their ability to transform the water-soluble yellow dye MTT to a purple formazan, which can be analyzed by UV–vis spectroscopy.

**Figure 14:**
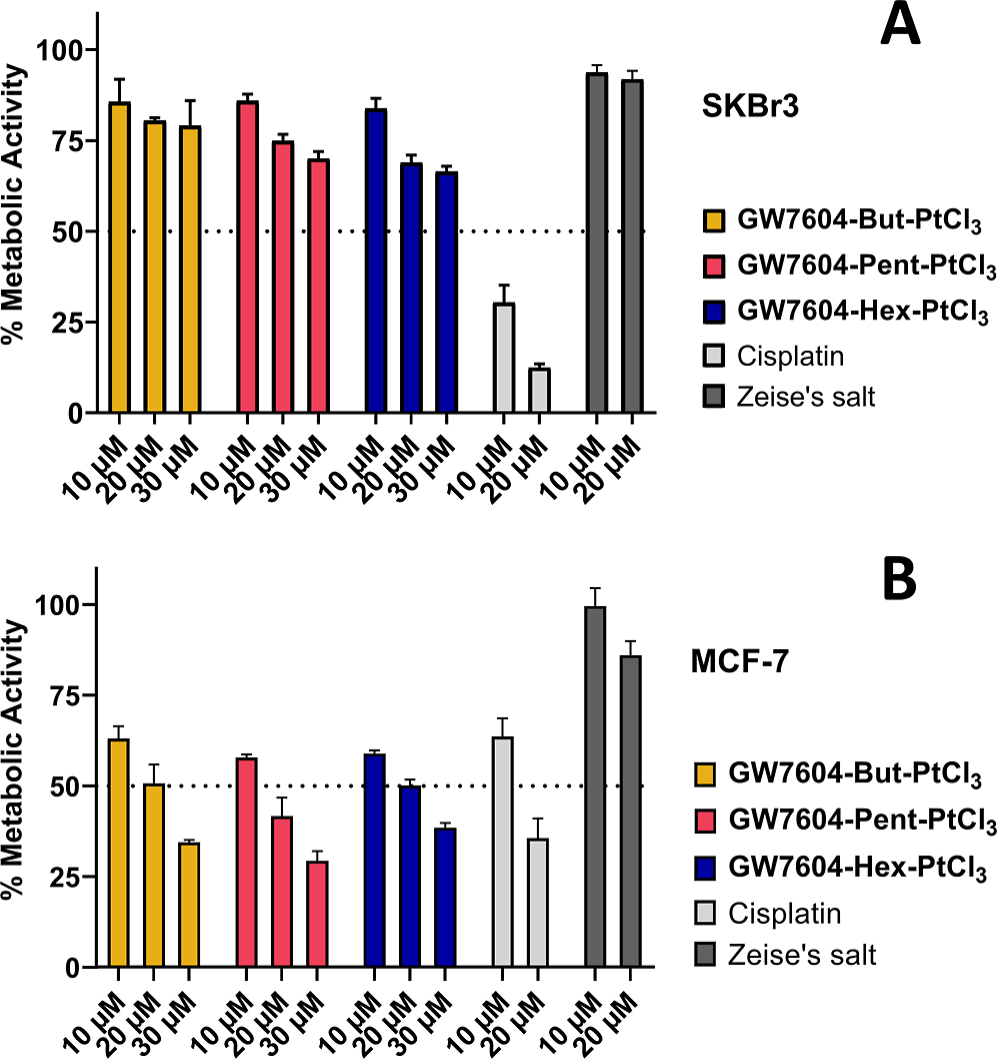
Investigation of the metabolic activity in ER-negative, COX-2-positive SKBr3 cells (A) and ERα-positive, COX-negative MCF-7 cells (B). Cells were incubated for 72 h. The reduction of metabolic activity was measured via an MTT assay. The results are given as the mean + SD of 3 independent experiments.

Zeise’s salt and its derivatives caused little or no cytotoxicity in SKBr3 cells (Figure 14A) due to their low cellular uptake. Even at the highest concentration of 30 μM, only a maximum inhibition of 25–30% was achieved. Cisplatin as a positive control reduced the metabolic activity at 20 μM by 88%.

In contrast, **GW7604-Alk-PtCl**_**3**_ complexes were highly active in MCF-7 cells, comparable to Cisplatin (Figure 14B). They reached a 50% inhibition at about 20 μM, with IC_50_ values of 20.4 μM (**GW7604-But-PtCl**_**3**_), 19.6 μM **(GW7604-Pent-PtCl**_**3**_), 20.5 μM (**GW7604-Hex-PtCl**_**3**_), and 15.0 μM (Cisplatin).

Interestingly, the effect of **GW7604-Pent-PtCl**_**3**_ did not correlate with the increased cellular uptake. The complex caused a 3-fold higher platinum amount in MCF-7 cells than Cisplatin. Nevertheless, the cytotoxic effects of both complexes are nearly the same. However, the IC_50_ value correlates more with the quantity of platinum determined in the nuclei. As depicted in Figure 5B, the values of **GW7604-Pent-PtCl**_**3**_ and Cisplatin are almost identical.

The correlation applies to SKBr3 cells, too. The high platinum content of 652 pmol/mg in the nuclei achieved with Cisplatin after 24 h led to superior cytotoxic effects. In contrast, cell viability was only slightly affected by **GW7604-Pent-PtCl**_**3**_, because of the low amount of only 96 pmol/mg in the nuclei.

The higher effect of **GW7604-Pent-PtCl**_**3**_ in MCF-7 cells compared to SKBr3 cells, despite almost comparable amounts of platinum in the nuclei, suggests that another mechanism of action may also be involved. An assessment as to whether the increased COX-2 inhibition is involved in the cytotoxic effect cannot yet be answered at this point in time.

Interestingly, the complexes possessed tumor cell-specific antimetabolic effects, as indicated by the inactivity in noncancerous TSA-201 cells at 20 μM (Figure S30). At higher concentrations (30 μM), only **GW7604-But-PtCl**_**3**_ and **GW7604-Pent-PtCl**_**3**_ decreased the cell viability by about 20%.

Although derived from **GW7604**, which reduced the growth of MCF-7 cells with an IC_50_ = 0.171 μM, the free alkenyl esters **GW7604-Alk** were inactive (Figure S31). This finding clearly demonstrates that the effects of the **GW7604-Alk-PtCl**_**3**_ complexes are mediated by the (en)PtCl_3_ moiety. This in turn justifies the use of Zeise’s salt as a lead structure for the development of cancer drugs. Conjugation with **GW7604** as a carrier, as performed in this study, introduced a new and promising class of drugs into the field of Medicinal Chemistry.

## Conclusions

In this study, we demonstrated that it is possible to optimize Zeise’s salt for potential therapeutic use. Replacing the ethylene with **GW7604-Alk** increased the stability in aqueous solution. While Zeise’s salt mainly decomposes in a redox reaction to acetaldehyde and platinum(0), **GW7604-Alk-PtCl**_**3**_ complexes showed only exchange of the chlorido leaving groups. It was further demonstrated in the example of **GW7604-Pent-PtCl**_**3**_ that the complexes coordinate not only amino acids (alanine) but also nucleotides (5′-GMP). Accordingly, binding to DNA was confirmed.

**GW7604-Alk** were designed as carrier ligands for the Zeise’s salt moiety. **GW7604**, an effective LBS binder, was expected to confer affinity to the ER and therefore selectivity to ER-positive tumor cells. In fact, **GW7604-Pent-PtCl**_**3**_ had high affinity to ERα and ERβ, even to a higher extent than the free ligand. It was accumulated in ERα-positive MCF-7 cells, while in ER-negative SKBr3 cells, distinctly lower amounts of platinum were determined.

Real-time live confocal microscopy identified in MCF-7 cells cytosol-localized fluorescent vesicles. It is very likely that the mER caused, after binding of **GW7604-Pent-PtCl**_**3**_, the formation of GVs. Collapse of these vesicles led to the release of the complexes, followed by their transfer into the nuclei and DNA binding, leading to the induction of cell death.

Hormonal activity as part of the mode of action can be excluded because neither agonistic effects nor ER downregulation were confirmed in MCF-7 cells.

Additionally, the complexes inhibited COX-1 and COX-2 activity in an assay with the isolated isoenzymes. The bound ligands significantly improved the inhibitory effect of **GW7604-Alk-PtCl**_**3**_ against COX-2 compared to Zeise’s salt.

**GW7604-Alk-PtCl**_**3**_ can therefore be classified as multitarget compounds with high selectivity for ER-positive tumor cells. The growth of noncancerous TSA-201 cells was not affected.

## Experimental Section

Chemical reagents and solvents were purchased from commercial suppliers (Sigma-Aldrich, BLDpharm, Fluka, Alfa Aesar, and Abcr) and were used without further purification. Thin-layer chromatography was carried out on Polygram SIL G/UV254 (Macherey-Nagel, Düren, Germany) precoated polyester sheets; the spots were visualized by UV light (254 nm). For column chromatography, silica gel 60 (0.040–0.063 mm, VWR, Darmstadt, Germany) was used. NMR spectra were recorded on a Bruker (Billerica, MA, USA) Avance 4 Neo 400 MHz spectrometer at 400.13 MHz (^1^H), 100.62 MHz (^13^C), and 85.88 MHz (^195^Pt) in CDCl_3_, acetone-*d*_6_, acetonitrile-*d*_3_, DMF-*d*_7_, methanol-*d*_4_, or DMSO-*d*_6_ (purchased from Eurisotop, Saint-Aubin, France) at an ambient temperature. Chemical shifts (δ) are given in parts per million (ppm) and were referenced relative to the internal standard tetramethylsilane (TMS) for ^1^H and ^13^C NMR spectroscopy or external to Na_2_[PtCl_6_] for ^195^Pt NMR spectroscopy. NMR data were processed using MestreNova v14 (Mestrelab, Krakow, Poland). ESI-HR-MS measurements were performed on an amaZon SL mass spectrometer (Bruker, Billerica, MA, USA) using direct infusion and electrospray ionization. Measurements were conducted in negative and positive ion modes. HR-MS data analysis was carried out with MestreNova v14. HPLC experiments (determination of purity and reactivity studies) were performed on a Shimadzu (Duisburg, Germany) prominence HPLC system (autosampler SIL-20A HT, column oven CTO-10AS VP, degasser DGU-20A, detector SPD-M20A, pumps LC-20AD, column ZORBAX Eclipse Plus; C18; 95 Å; 5 μm; 4.6 × 250 mm). The mobile phase consisted of ACN and water. Separation of the complexes from the reaction product was possible with isocratic elution at 90% ACN, a flow rate of 1 mL/min, and an oven temperature of 35 °C. All solvents were degassed before use. The injection volume was 30 μL, and the UV–vis detection wavelength was set at 254 nm. The software used for data processing was LabSolutions (Shimadzu, Duisburg, Germany). The purity (>95%) of all biotested compounds was confirmed by HPLC and elemental analysis.

### Synthesis and Characterization

#### (*E*)-3-(4-((*E/Z*)-1-(4-((*tert*-butyldimethylsilyl)oxy)phenyl)-2-phenylbut-1-en-1-yl)phenyl)acrylic Acid (**1**)

A mixture of (*E*)-3-(4-((*E/Z*)-1-(4-hydroxyphenyl)-2-phenylbut-1-en-1-yl)phenyl)acrylic acid **GW7604** (1.58 g, 4.26 mmol), imidazole (0.87 g, 12.78 mmol), and TBDMSCl (1.41 g, 9.38 mmol) was dissolved in dry DMF (15 mL) and stirred overnight at rt. Afterward, the reaction mixture was poured into water and extracted with ethyl acetate (3 × 30 mL). The combined organic layers were washed with brine, dried over Na_2_SO_4_, filtered, and concentrated *in vacuo* to obtain a yellow oil. The crude oil was diluted in 6 mL of a mixture of MeOH/THF [1/2, (*v/v*)] and stirred at rt with continuous dropwise addition of K_2_CO_3_ solution (5 mL; 0.5 N aq.) over 3 h. The reaction was monitored by thin-layer chromatography. Then, the solution was evaporated to dryness. The residue was dissolved in DCM, washed with water and brine, dried over Na_2_SO_4_, and evaporated to dryness. After purification by flash chromatography on silica gel with DCM/MeOH [9.5/0.5 (*v/v*)], **1** was obtained as a brown/yellow solid (1.45 g, 2.99 mmol, 70%). ^1^H NMR (400 MHz, CDCl_3_, *E/Z* mixture): δ 0.22 (s, 6H, *tert*-butyl-Si(CH_3_)_2_), 0.92 (2 × t, *J* = 7.4 Hz, 3H, CH_2_CH_3_, *E/Z* isomer), 1.00 (s, 9H, *tert*-butyl-Si(CH_3_)_2_), 2.49 (2 × q, *J* = 7.4 Hz, 12.5 Hz, 2H, CH_2_CH_3_, *E/Z* isomer), 6.28 (d, *J* = 16.0 Hz, 0.7H, CH=CHCOOH, *E* isomer), 6.45 (d, *J* = 15.9 Hz, 0.3H, CH=CHCOOH, *Z* isomer), 6.49 (d, *J* = 8.6 Hz, 0.5H, Ar–H, *Z* isomer), 6.69 (d, *J* = 8.6 Hz, 0.5H, Ar–H, *Z* isomer), 6.82 (d, *J* = 8.5 Hz, 1.4H, Ar–H, *E* isomer), 6.89 (d, *J* = 8.3 Hz, 1.4H, Ar–H, *E* isomer), 7.03–7.23 (m, 8.2H, Ar–H), 7.29 (d, *J* = 8.2 Hz, 0.5H, Ar–H, *Z* isomer), 7.54 (d, *J* = 8.2 Hz, 0.5H, Ar–H, *Z* isomer), 7.62 (d, *J* = 16.0 Hz, 0.7H, CH=CHCOOH, *E* isomer), and 7.80 (d, *J* = 16.0 Hz, 0.3H, CH=CHCOOH, *Z* isomer).

#### Synthesis of GW7604-Alkenyl Esters (**2a**–**d**) General Method

Compound **1**, DMAP, and the respective alkenol were dissolved in 6 mL of dry DCM. The reaction mixture was cooled to 0 °C, and EDC was added. The solution was stirred at 0 °C for 1 h and for further 16 h at rt. The solvent was evaporated, and the crude product was purified by flash chromatography on silica gel with DCM/MeOH [9.5/0.5 (*v/v*)] to obtain the **GW7604**-alkenyl esters as a yellow sticky oil.

#### Prop-2-en-1-yl (*E*)-3-(4-((*E/Z*)-1-(4-((*tert*-butyldimethylsilyl)oxy)phenyl)-2-phenylbut-1-en-1-yl)phenyl) Acrylate (**2a**)

From compound **1** (360 mg 0.740 mmol), DMAP (10 mg, 0.074 mmol), prop-2-en-1-ol (0.10 mL, 1.490 mmol), and EDC (140 μL, 0.890 mmol). Yield: 240 mg, 0.460 mmol, 61%. ^1^H NMR (400 MHz, CDCl_3_, *E/Z* mixture): δ 0.10 (s, 1.8H, *tert*-butyl-Si(CH_3_)_2_, *Z* isomer), 0.22 (s, 4.2H, *tert*-butyl-Si(CH_3_)_2_, *E* isomer), 0.91 (s, 3.3H, *tert*-butyl-Si(CH_3_)_2_, *Z* isomer), 0.91–0.97 (m, 3H, CH_2_CH_3_), 1.00 (s, 5.7H, *tert*-butyl-Si(CH_3_)_2_, *E* isomer), 2.49 (2 × q, *J* = 11.7 Hz, 7.4 Hz, 2H, CH_2_CH_3_), 4.70 (ddt, *J* = 22.0 Hz, 5.6 Hz, 1.4 Hz, 2H, OCH_2_CH=CH_2_), 5.18–5.48 (m, 2H, OCH_2_CH=CH_2_), 5.88–6.08 (m, 1H, OCH_2_CH=CH_2_), 6.30 (d, *J* = 16.0 Hz, 0.7H, CH=CHCO, *E* isomer), 6.43–6.49 (m, 0.9H, CH=CHCO, *Z* isomer + Ar–H, *Z* isomer), 6.69 (d, *J* = 8.6 Hz, 0.6H, Ar–H, *Z* isomer), 6.82 (d, *J* = 8.5 Hz, 1.5H, Ar–H, *E* isomer), 6.88 (d, *J* = 8.3 Hz, 1.5H, Ar–H, *E* isomer), 7.02–7.20 (m, 8H, Ar–H), 7.27 (0.5H, *J* = 8.2 Hz, Ar–H, *Z* isomer) 7.51 (d, *J* = 8.2 Hz, 0.2H, Ar–H), 7.55 (d, *J* = 16.0 Hz, 0.7H, CH=CHCO, *E* isomer), and 7.73 (d, *J* = 16.0 Hz, 0.3H, CH=CHCO, *Z* isomer).

#### But-3-en-1-yl (*E*)-3-(4-((*E/Z*)-1-(4-((*tert*-butyldimethylsilyl)oxy)phenyl)-2-phenylbut-1-en-1-yl)phenyl) Acrylate (**2b**)

From compound **1** (230 mg, 0.460 mmol), DMAP (28 mg, 0.230 mmol), but-3-en-1-ol (80 μL, 0.930 mmol), and EDC (97 μL, 0.550 mmol). Yield: 190 mg, 0.350 mmol, 76%. ^1^H NMR (400 MHz, CDCl_3_, *E/Z* mixture): δ 0.10 (s, 1.8H, *tert*-butyl-Si(CH_3_)_2_, *Z* isomer), 0.22 (s, 4.2H, *tert*-butyl-Si(CH_3_)_2_, *E* isomer), 0.91 (s, 3.3H, *tert*-butyl-Si(CH_3_)_2_, *Z* isomer), 0.91–0.97 (m, 3H, CH_2_CH_3_), 1.00 (s, 5.7H, *tert*-butyl-Si(CH_3_)_2_, *E* isomer), 2.35–2.60 (m, 4H, CH_2_CH_3_ + OCH_2_CH_2_CH=CH_2_), 4.24 (dt, *J* = 6.8 Hz, 22.1 Hz, 2H, OCH_2_CH_2_CH=CH_2_), 4.96–5.20 (m, 2H, OCH_2_CH_2_CH=CH_2_), 5.72–5.95 (m, 1H, OCH_2_CH_2_CH=CH_2_), 6.28 (d, *J* = 16.0 Hz, 0.7H, CH=CHCO, *E* isomer), 6.44 (d, *J* = 16.0 Hz, 0.3H, CH=CHCO, *Z* isomer), 6.48 (d, *J* = 8.6 Hz, 0.3H, Ar–H, *Z* isomer), 6.69 (d, *J* = 8.6 Hz, 0.3H, Ar–H, *Z* isomer), 6.82 (d, *J* = 8.5 Hz, 1.5H, Ar–H, *E* isomer), 6.87 (d, *J* = 8.4 Hz, 1.5H, Ar–H, *E* isomer), 7.02–7.22 (m, 8H, Ar–H), 7.27 (d, *J* = 6.7 Hz, 0.5H, Ar–H), 7.43–7.58 (m, 1.20H, Ar–H, CH=CHCO, *E* isomer), and 7.70 (d, *J* = 16.0 Hz, 0.3H, CH=CHCO, *Z* isomer).

#### Pent-4-en-1-yl (*E*)-3-(4-((*E/Z*)-1-(4-((*tert*-butyldimethylsilyl)oxy)phenyl)-2-phenylbut-1-en-1-yl)phenyl) Acrylate (**2c**)

From compound **1** (650 mg, 1.340 mmol), DMAP (16 mg, 0.134 mmol), pent-4-en-1-ol (280 μL, 2.680 mmol), and EDC (283 μL, 1.600 mmol). Yield: 400 mg, 0.730 mmol, 55%. ^1^H NMR (400 MHz, CDCl_3_, *E/Z* mixture): δ 0.10 (s, 3.8H, *tert*-butyl-Si(CH_3_)_2_, *Z* isomer), 0.23 (s, 2.2H, *tert*-butyl-Si(CH_3_)_2_, *E* isomer), 0.91 (s, 5.8H, *tert*-butyl-Si(CH_3_)_2_, *Z* isomer), 0.91–0.97 (m, 3H, CH_2_CH_3_), 1.00 (s, 3.2H, *tert*-butyl-Si(CH_3_)_2_, *E* isomer), 1.80 (ddq, *J* = 6.7 Hz, 8.7 Hz, 19.4 Hz, 2H, OCH_2_CH_2_CH_2_CH=CH_2_), 2.11–2.23 (m, 2H, OCH_2_CH_2_CH_2_CH=CH_2_), 2.35–2.60 (m, 2H, CH_2_CH_3_), 4.20 (dt, *J* = 6.6 Hz, 22.1 Hz, 2H, OCH_2_CH_2_CH_2_CH=CH_2_), 4.92–5.17 (m, 2H, OCH_2_CH_2_CH_2_CH=CH_2_), 5.72–5.95 (m, 1H, OCH_2_CH_2_CH_2_CH=CH_2_), 6.28 (d, *J* = 15.9 Hz, 0.4H, CH=CHCO, *E* isomer), 6.45 (d, *J* = 16.1 Hz, 0.6H, CH=CHCO, *Z* isomer), 6.49 (d, *J* = 8.6 Hz, 1.3H, Ar–H, *Z* isomer), 6.69 (d, *J* = 8.6 Hz, 1.3H, Ar–H, *Z* isomer), 6.82 (d, *J* = 8.5 Hz, 0.7H, Ar–H, *E* isomer), 6.87 (d, *J* = 8.4 Hz, 0.7H, Ar–H, *E* isomer), 7.02–7.23 (m, 6.9H, Ar–H), 7.27 (d, *J* = 6.7 Hz, 1H, Ar–H), 7.46–7.57 (m, 1.5H, Ar–H, CH=CHCO, *E* isomer), and 7.70 (d, *J* = 16.0 Hz, 0.6H, CH=CHCO, *Z* isomer).

#### Hex-5-en-1-yl (*E*)-3-(4-((*E/Z*)-1-(4-((*tert*-butyldimethylsilyl)oxy)phenyl)-2-phenylbut-1-en-1-yl)phenyl) Acrylate (**2d**)

From compound **1** (400 mg, 0.830 mmol), DMAP (10 mg, 0.082 mmol), hex-5-en-1-ol (170 μL, 1.650 mmol), and EDC (176 μL, 1.000 mmol). Yield: 280 mg, 0.500 mmol, 61%. ^1^H NMR (400 MHz, CDCl_3_, *E/Z* mixture): δ 0.10 (s, 3.8H, *tert*-butyl-Si(CH_3_)_2_, *Z* isomer), 0.23 (s, 2.2H, *tert*-butyl-Si(CH_3_)_2_, *E* isomer), 0.91 (s, 5.8H, *tert*-butyl-Si(CH_3_)_2_, *Z* isomer), 0.91–0.97 (m, 3H, CH_2_CH_3_), 1.00 (s, 3.2H, *tert*-butyl-Si(CH_3_)_2_, *E* isomer), 1.41–1.80 (m, 4H, OCH_2_CH_2_CH_2_CH_2_CH=CH_2_), 2.11 (dq, *J* = 7.1, 14.0 Hz, 2H, OCH_2_CH_2_CH_2_CH_2_CH=CH_2_), 2.49 (dq, *J* = 7.4, 11.9 Hz, 2H, CH_2_CH_3_), 4.19 (dt, *J* = 6.6, 22.0 Hz, 2H, OCH_2_CH_2_CH_2_CH_2_CH=CH_2_), 4.87–5.12 (m, 2H, OCH_2_CH_2_CH_2_CH_2_CH=CH_2_), 5.72–5.95 (m, 1H, OCH_2_CH_2_CH_2_CH_2_CH=CH_2_), 6.28 (d, *J* = 15.9 Hz, 0.4H, CH=CHCO, *E* isomer), 6.45 (d, *J* = 16.1 Hz, 0.6H, CH=CHCO, *Z* isomer), 6.49 (d, *J* = 8.6 Hz, 1.3H, Ar–H, *Z* isomer), 6.69 (d, *J* = 8.6 Hz, 1.3H, Ar–H, *Z* isomer), 6.82 (d, *J* = 8.5 Hz, 0.7H, Ar–H, *E* isomer), 6.87 (d, *J* = 8.4 Hz, 0.7H, Ar–H, *E* isomer), 7.02–7.23 (m, 6.9H, Ar–H), 7.27 (d, *J* = 6.7 Hz, 1H, Ar–H), 7.46–7.57 (m, 1.5H, Ar–H, CH=CHCO, *E* isomer), and 7.70 (d, *J* = 16.0 Hz, 0.6H, CH=CHCO, *Z* isomer).

#### Synthesis of **GW7604-Alk**. General Method

To an ice-cold (0 °C) solution of **2a**-**d** in dry THF (3 mL), TBAF was added. The reaction mixture was stirred under these conditions for 1 h and then for further 4 h at rt. After evaporation of the solvent, the residue was purified by column chromatography on silica gel with ethyl acetate/petroleum ether [3/7 (*v/v*)] to give the final product as a yellow solid.

#### Prop-2-en-1-yl (*E*)-3-(4-((*E/Z*)-1-(4-hydroxyphenyl)-2-phenylbut-1-en-1-yl)phenyl) Acrylate (**GW7604-Prop**)

From **2a** (230 mg, 0.450 mmol) and TBAF (490 μL of a 1 N solution in THF, 0.490 mmol). Yield: 180 mg, 0.450 mmol, quant. ^1^H NMR (400 MHz, acetone-*d*_6_, *E*/*Z* mixture): δ 0.92 (t, *J* = 7.4 Hz, 3H, CH_2_CH_3_), 2.50 (2 × q, *J* = 7.5 Hz, 18.3 Hz, 2H, CH_2_CH_3_), 4.67 (ddt, *J* = 1.5 Hz, 5.5 Hz, 21.1 Hz, 2H, OCH_2_CH=CH_2_), 5.10–5.45 (m, 2H, OCH_2_CH=CH_2_), 5.88–6.07 (m, 1H, OCH_2_CH=CH_2_), 6.43 (d, *J* = 16.0 Hz, 0.6H, CH=CHCO, *E* isomer), 6.51 (d, *J* = 8.6 Hz, 0.5H, Ar–H, *Z* isomer), 6.60 (d, *J* = 16.0 Hz, 0.4H, CH=CHCO, *E* isomer), 6.73 (d, *J* = 8.7 Hz, 0.5H, Ar–H, *Z* isomer), 6.87 (d, *J* = 8.5 Hz, 1.3H, Ar–H, *E* isomer), 6.95 (d, *J* = 8.4 Hz, 1.3H, Ar–H, *E* isomer), 7.10 (d, *J* = 8.5 Hz, 1.3H, Ar–H, *E* isomer), 7.14–7.20 (m, 5.75H, Ar–H), 7.28–7.40 (m, 2H, Ar–H), 7.55 (d, *J* = 16.0 Hz, 0.6H, CH=CHCO, *E* isomer), 7.68–7.79 (m, 0.75H, Ar–H + CH=CHCO, *Z* isomer), 8.13 (s, 0.4H, OH, *Z* isomer), and 8.37 (s, 0.6H, OH, *E* isomer). ^13^C NMR (101 MHz, acetone-*d*_6_, *E/Z* mixture): δ 13.75, 13.80, 23.31, 29.57, 29.72, 65.30, 65.41, 115.25, 115.92, 115.97, 117.88, 117.99, 118.44, 126.99, 127.17, 128.16, 128.69, 128.77, 128.99, 130.49, 130.81, 131.22, 131.39, 132.08, 132.61, 132.67, 133.69, 133.78, 134.71, 135.02, 139.00, 139.13, 142.42, 143.06, 143.13, 143.52, 145.23, 145.26, 146.97, 147.18, 156.49, 157.33, 166.68, and 166.73.

#### But-3-en-1-yl (*E*)-3-(4-((*E/Z*)-1-(4-hydroxyphenyl)-2-phenylbut-1-en-1-yl)phenyl) Acrylate (**GW7604-But**)

From **2b** (190 mg, 0.350 mmol) and TBAF (380 μL of a 1 N solution in THF, 0.380 mmol). Yield: 140 mg, 0.330 mmol, 93%. ^1^H NMR (400 MHz, acetone-*d*_6_, *E/Z* mixture): δ 0.92 (t, *J* = 7.4 Hz, 3H, CH_2_CH_3_), 2.37–2.56 (m, 4H, CH_2_CH_3_ + OCH_2_CH_2_CH=CH_2_), 4.21 (2 × t, *J* = 6.7 Hz, 2H, OCH_2_CH_2_CH=CH_2_), 5.00–5.21 (m, 2H, OCH_2_CH_2_CH=CH_2_), 5.76–5.90 (m, 1H, OCH_2_CH_2_CH=CH_2_), 6.39 (d, *J* = 16.0 Hz, 0.6H, CH=CHCO, *E* isomer), 6.51 (d, *J* = 8.7 Hz, 0.7H, Ar–H, *Z* isomer), 6.56 (d, *J* = 16.0 Hz, 0.4H, CH=CHCO, *Z* isomer), 6.72 (d, *J* = 8.7 Hz, 0.7H, Ar–H, *Z* isomer), 6.87 (d, *J* = 8.5 Hz, 1.3H, Ar–H, *E* isomer), 6.95 (d, *J* = 8.3 Hz, 1.3H, Ar–H, *E* isomer), 7.06–7.22 (m, 6.4H, Ar–H), 7.27–7.36 (m, 2H, Ar–H), 7.52 (d, *J* = 16.0 Hz, 0.6H, CH=CHCO, *E* isomer), 7.66–7.75 (m, 1H, Ar–H + CH=CHCO, *Z* isomer), 8.15 (s, 0.4H, OH, *Z* isomer), and 8.40 (s, 0.6H, OH, *E* isomer). ^13^C NMR (101 MHz, acetone-*d*_6_, *E/Z* mixture): δ 13.75, 13.80, 26.20, 33.94, 63.91, 64.01, 115.27, 115.98, 117.34, 117.39, 118.23, 118.67, 127.01, 127.18, 128.14, 128.71, 128.79, 128.97, 130.49, 130.51, 130.82, 131.40, 132.09, 132.68, 133.74, 134.73, 135.04, 135.39, 135.41, 139.04, 139.17, 142.43, 143.09, 143.16, 143.52, 144.99, 145.01, 146.93, 147.14, 156.53, 157.37, 167.01, and 167.06.

#### Pent-4-en-1-yl (*E*)-3-(4-((*E/Z*)-1-(4-hydroxyphenyl)-2-phenylbut-1-en-1-yl)phenyl) Acrylate (**GW7604-Pent**)

From **2c** (310 mg, 0.570 mmol) and TBAF (630 μL of a 1 N solution in THF, 0.630 mmol). Yield: 140 mg, 0.310 mmol, 55%. ^1^H NMR (400 MHz, acetone-*d*_6_, *E/Z* mixture): δ 0.92 (t, *J* = 7.4 Hz, 3H, CH_2_CH_3_), 1.78 (2 × q, *J* = 6.7 Hz, 17.7 Hz, 2H, OCH_2_CH_2_CH_2_CH=CH_2_), 2.09–2.21 (m, 2H, OCH_2_CH_2_CH_2_CH=CH_2_), 2.50 (2 × q, *J* = 7.4 Hz, 2H, CH_2_CH_3_), 4.17 (dt, *J* = 6.6 Hz, 21.2 Hz, 2H, OCH_2_CH_2_CH_2_CH=CH_2_), 4.92–5.12 (m, 2H, OCH_2_CH_2_CH_2_CH=CH_2_), 6.40 (d, *J* = 16.0 Hz, 1H, OCH_2_CH_2_CH_2_CH=CH_2_), 6.51 (d, *J* = 8.6 Hz, 0.4H, CH=CHCO, *E* isomer), 6.57 (d, *J* = 16.1 Hz, 1.2H, Ar–H, *Z* isomer), 6.72 (d, *J* = 8.7 Hz, 0.6H, CH=CHCO, *Z* isomer), 6.87 (d, *J* = 8.6 Hz, 0.8H, Ar–H, *E* isomer), 6.95 (d, *J* = 8.3 Hz, 0.8H, Ar–H, *E* isomer), 7.07–7.23 (m, 7.2H, Ar–H), 7.29–7.36 (m, 2H, Ar–H), 7.53 (d, *J* = 16.0 Hz, 0.4H, CH=CHCO, *E* isomer), 7.65–7.78 (m, 1.6H, Ar–H + CH=CHCO, *Z* isomer), 8.15 (s, 0.4H, OH, *Z* isomer), and 8.46 (s, 0.6H, OH, *E* isomer). ^13^C NMR (101 MHz, acetone-*d*_6_, *E/Z* mixture): δ 13.76, 13.81, 28.71, 30.76, 64.16, 64.27, 115.24, 115.46, 115.49, 115.95, 118.28, 118.73, 121.60, 122.52, 126.97, 127.15, 128.09, 128.25, 128.68, 128.76, 128.83, 128.92, 129.07, 130.45, 130.78, 131.38, 132.06, 132.32, 132.66, 133.73, 134.69, 135.01, 138.65, 138.99, 139.12, 142.37, 143.05, 143.11, 143.47, 144.85, 144.88, 146.85, 147.07, 156.47, 157.30, 167.06, and 167.10.

#### Hex-5-en-1-yl (*E*)-3-(4-((*E/Z*)-1-(4-hydroxyphenyl)-2-phenylbut-1-en-1-yl)phenyl) Acrylate (**GW7604-Hex**)

From **2d** (310 mg, 0.570 mmol) and TBAF (630 μL of a 1 N solution in THF, 0.630 mmol). Yield: 140 mg, 0.310 mmol, 55%. ^1^H NMR (400 MHz, acetone-*d*_6_, *E/Z* mixture) δ 0.90 (t, *J* = 7.4 Hz, 3H, CH_2_CH_3_), 1.38–1.57 (m, 2H, OCH_2_CH_2_CH_2_CH_2_CH=CH_2_), 1.67 (2 × q, *J* = 6.7 Hz, 2H, OCH_2_CH_2_CH_2_CH_2_CH=CH_2_), 2.03–2.12 (m, 2H, OCH_2_CH_2_CH_2_CH_2_CH=CH_2_), 2.50 (2 × q, *J* = 7.4 Hz, 2H, CH_2_CH_3_), 4.15 (dt, *J* = 6.6 Hz, 21.2 Hz, 2H, OCH_2_CH_2_CH_2_CH_2_CH=CH_2_), 4.86–5.06 (m, 2H, OCH_2_CH_2_CH_2_CH_2_CH=CH_2_), 5.81 (dtt, *J* = 6.7 Hz, 10.1 Hz, 16.9 Hz, 1H, OCH_2_CH_2_CH_2_CH_2_CH=CH_2_), 6.38 (d, *J* = 16.0 Hz, 0.4H, CH=CHCO, *E* isomer), 6.49 (d, *J* = 8.6 Hz, 1H, Ar–H, *Z* isomer), 6.55 (d, *J* = 16.1 Hz, 0.6H, CH=CHCO, *Z* isomer), 6.70 (d, *J* = 8.6 Hz, 1H, Ar–H, *Z* isomer), 6.85 (d, *J* = 8.6 Hz, 0.9H, Ar–H, *E* isomer), 6.93 (d, *J* = 8.3 Hz, 0.9H, Ar–H, *E* isomer), 7.05–7.21 (m, 7.45H, Ar–H), 7.27–7.35 (m, 1.85H, Ar–H), 7.50 (d, *J* = 16.0 Hz, 0.4H, CH=CHCO, *E* isomer), 7.65–7.72 (m, 1.5H, Ar–H + CH=CHCO, *Z* isomer), 8.10 (s, 0.4H), and 8.35 (s, 0.6H). ^13^C NMR (101 MHz, acetone-*d*_6_, *E/Z* mixture): δ 3.21, 13.75, 13.81, 25.96, 25.99, 26.19, 33.98, 34.01, 41.11, 64.62, 64.73, 115.11, 115.14, 115.24, 115.95, 118.33, 118.78, 126.97, 127.15, 127.30, 128.09, 128.43, 128.68, 128.76, 128.92, 129.70, 130.46, 130.47, 130.78, 131.38, 132.06, 132.66, 133.74, 134.70, 135.01, 139.00, 139.13, 139.35, 142.38, 143.06, 143.13, 143.47, 144.82, 144.85, 146.85, 147.06, 156.47, 157.31, 167.08, and 167.12.

### Synthesis of GW7604-Alk-PtCl_3_ Complexes

#### General Procedure

Under an argon atmosphere, 1.0 equivalent (eq) of Zeise’s salt was dissolved in degassed (three cycles of freeze–pump–thaw) dry EtOH. The suspension was stirred at rt under protection from light. Then, a slight excess of 1.2 eq of the respective ester (**GW7604-Alk**) dissolved in approximately 1 mL of degassed and dry EtOH was added dropwise. After addition, the mixture was stirred at 48–50 °C for 4 h. The mixture was then allowed to cool to rt, and the solvent was evaporated. Recrystallization from acetone/diethyl ether afforded the pure solid products.

Potassium trichlorido[η^2^-(prop-2-en-1-yl (*E*)-3-(4-((*E/Z*)-1-(4-hydroxyphenyl)-2-phenylbut-1-en-1-yl)phenyl)acrylate)]platinate(II) (**GW7604-Prop-PtCl**_**3**_). From Zeise’s salt (39 mg, 0.100 mmol) and **GW7604-Prop** (48 mg, 0.120 mmol), dissolved in 4 mL of degassed and dry EtOH. Orange solid. Yield: 25 mg, 0.033 mmol, 33%. Purity calculated by HPLC (peak area): > 95%. ^1^H NMR (400 MHz, acetone-*d*_*6*_, *E/Z* mixture): δ 0.92 (2 × t, *J* = 7.4 Hz, 3H, CH_2_CH_3_), 2.50 (2 × q, *J* = 7.4 Hz, 17.8 Hz, 2H, CH_2_CH_3_), 4.15–4.31 (m, 2H, C=CH_2_), 4.31–4.45 (m, *J*_*H–P*t_ = 32 Hz, 1H, -OCH_α_H_β_-), 4.73–4.86 (m, *J_H–Pt_* = 32 Hz, 1H, -OCH_α_H_β_-), 4.88–5.06 (m, *J_H–Pt_* = 60 Hz, 1H, –CH=C), 6.44 (d, *J* = 16.0 Hz, 0.6H, CH=CHCO, E isomer), 6.51 (d, *J* = 8.6 Hz, 0.8H, Ar–H, *Z* isomer), 6.61 (d, *J* = 16.0 Hz, 0.4H, CH=CHCO, *Z* isomer), 6.72 (d, *J* = 8.6 Hz, 0.8H, Ar–H, *Z* isomer), 6.87 (d, *J* = 8.6 Hz, 1.2H, Ar–H, *E* isomer), 6.94 (d, *J* = 8.3 Hz, 1.2H, Ar–H, *E* isomer), 7.06–7.22 (m, 6.2H, Ar–H), 7.27–7.41 (m, 2H, Ar–H, *E + Z* isomer), 7.58 (d, *J* = 16.0 Hz, 0.6H, CH=CHCO, *E* isomer), 7.72 (d, *J* = 8.2 Hz, 0.8H, Ar–H, *Z* isomer), 7.77 (d, *J* = 16.0 Hz, 0.4H, CH=CHCO, *Z* isomer), 8.13 (s, 0.3H, OH, *Z* isomer), and 8.38 (s, 0.7H, OH, *E* isomer). ^13^C NMR (101 MHz, acetone-*d*_*6*_, *E/Z* mixture): δ 13.72, 13.77, 15.58, 30.48, 64.54, 64.63, 65.07, 66.07, 78.36, 115.14, 115.23, 115.86, 115.95, 118.06, 118.52, 126.96, 127.15, 128.18, 128.56, 128.67, 128.76, 129.01, 130.47, 130.76, 131.37, 132.04, 132.65, 132.70, 133.77, 134.71, 135.02, 139.02, 139.15, 142.38, 143.04, 143.15, 143.46, 145.26, 146.89, 147.09, 156.46, 157.30, 166.93, and 166.97. ^195^Pt NMR (86 MHz, acetone-*d*_*6*_): δ –2683. Elemental Anal. Calc. for C_28_H_26_Cl_3_KO_3_Pt: C, 44.78; H, 3.49; N, 0.00. Found: C, 44.38; H, 3.74; N, 0.00. ESI-HR-MS (*m*/*z*): calculated for C_28_H_26_Cl_3_O_3_Pt [M – K]^−^: 711.0595, found 711.0593.

Potassium trichlorido[η^2^-(but-3-en-1-yl (*E*)-3-(4-((*E/Z)*-1-(4-hydroxyphenyl)-2-phenylbut-1-en-1-yl)phenyl)acrylate)]platinate(II) (**GW7604-But-PtCl**_**3**_). From Zeise’s salt (95 mg, 0.260 mmol) and **GW7604-But** (120 mg, 0.280 mmol), dissolved in 5 mL of degassed and dry EtOH. Yellow solid. Yield: 84 mg, 0.110 mmol, 39%. Purity calculated by HPLC (peak area): > 95%. ^1^H NMR (400 MHz, acetone-*d*_*6*_, *E/Z* mixture): δ 0.92 (2 × t, *J* = 7.4 Hz, 3H, CH_2_CH_3_), 1.87–2.12 (m, –CH_2_–, 2H), 2.50 (2 × q, *J* = 7.4 Hz, 17.8 Hz, 2H, CH_2_CH_3_), 4.09–4.36 (m, *J_H–Pt_* = 56 Hz, 2H, C=CH_2_), 4.44–4.64 (m, 2H, –OCH_2_−), 4.84–5.14 (m, *J_H–Pt_* = 60 Hz, 1H, –CH=C), 6.44 (d, *J* = 16.0 Hz, 0.45H, CH=CHCO, *E* isomer), 6.51 (d, *J* = 8.6 Hz, 1H, Ar–H, *Z* isomer), 6.61 (d, *J* = 16.0 Hz, 0.5H, CH=CHCO, *Z* isomer), 6.72 (d, *J* = 8.6 Hz, 1H, Ar–H, *Z* isomer), 6.87 (d, *J* = 8.6 Hz, 1H, Ar–H, *E* isomer), 6.94 (d, *J* = 8.3 Hz, 1H, Ar–H, *E* isomer), 7.06–7.22 (m, 6.55H, Ar–H), 7.27–7.41 (m, 2H, Ar–H, *E + Z* isomer), 7.58 (d, *J* = 16.0 Hz, 0.5H, CH=CHCO, *E* isomer), 7.72 (d, *J* = 8.2 Hz, 1H, Ar–H, *Z* isomer), 7.77 (d, *J* = 16.0 Hz, 0.5H, CH=CHCO, *Z* isomer), 8.12 (s, 0.6H, OH, *Z* isomer), and 8.37 (s, 0.4H, OH, *E* isomer). ^13^C NMR (101 MHz, acetone-*d*_*6*_, *E/Z* mixture): δ 13.72, 13.77, 33.46, 33.48, 63.89, 63.99, 65.87, 65.89, 84.71, 115.14, 115.23, 115.86, 115.95, 118.32, 118.78, 126.96, 127.16, 128.16, 128.67, 128.72, 128.76, 128.99, 130.47, 130.75, 131.17, 131.37, 132.03, 132.65, 132.75, 133.83, 134.72, 135.03, 139.03, 139.16, 142.36, 143.05, 143.16, 143.44, 145.03, 146.83, 147.03, 156.46, 157.30, 167.17, and 167.20. ^195^Pt NMR (86 MHz, acetone-*d_6_*): δ –2705. Elemental Anal. Calc. for C_29_H_28_Cl_3_KO_3_Pt: C, 45.53; H, 3.69; N, 0.00. Found: C, 45.85; H, 3.99; N, 0.00. ESI-HR-MS (*m*/*z*): calculated for C_29_H_28_Cl_3_O_3_Pt [M – K]^−^: 725.0752, found 725.0741.

Potassium trichlorido[η^2^-(pent-4-en-1-yl (*E*)-3-(4-((*E/Z*)-1-(4-hydroxyphenyl)-2-phenylbut-1-en-1-yl)phenyl)acrylate)]platinate(II) (**GW7604-Pent-PtCl**_**3**_). From Zeise’s salt (95 mg, 0.260 mmol) and **GW7604-Pent** (196 mg, 0.450 mmol), dissolved in 5 mL of degassed and dry EtOH. Yellow solid. Yield: 130 mg, 0.170 mmol, 39%. Purity calculated by HPLC (peak area): > 95%. ^1^H NMR (400 MHz, acetone-*d_6_*, *E/Z* mixture): δ 0.92 (2 × t, *J* = 7.4 Hz, 3H, CH_2_CH_3_), 1.58–1.76 (m, –CH_2_–, 2H), 1.97–2.10 (m, –CH_2_–, 2H), 2.14–2.36 (m, 2H), 2.50 (2 × q, *J* = 7.4 Hz, 18.9 Hz, 2H, CH_2_CH_3_), 4.03–4.39 (m, 4H, –OCH_2_– + C=CH_2_), 4.81–5.15 (*J_H–Pt_* = 66 Hz, 1H, –CH=C), 6.42 (d, *J* = 16.0 Hz, 0.5H, CH=CHCO, E isomer), 6.51 (d, *J* = 8.6 Hz, 1H, Ar–H, *Z* isomer), 6.59 (d, *J* = 16.0 Hz, 0.5H, CH=CHCO, *Z* isomer), 6.72 (d, *J* = 8.6 Hz, 1H, Ar–H, *Z* isomer), 6.87 (d, *J* = 8.6 Hz, 1H, Ar–H, *E* isomer), 6.94 (d, *J* = 8.3 Hz, 1H, Ar–H, *E* isomer), 7.06–7.23 (m, 6H, Ar–H), 7.27–7.41 (m, 2H, Ar–H, *E + Z* isomer), 7.54 (d, *J* = 16.0 Hz, 0.5H, CH=CHCO, *E* isomer), 7.71 (d, *J* = 8.2 Hz, 1H, Ar–H), 7.75 (d, *J* = 16.0 Hz, 0.5H, CH=CHCO, *E* isomer), 8.12 (s, 0.4H, OH, *Z* isomer), and 8.37 (s, 0.6H, OH, *E* isomer). ^13^C NMR (101 MHz, acetone-*d*_*6*_, *E/Z* mixture): δ 13.72, 13.77, 15.58, 29.02, 29.06, 30.65, 30.70, 64.56, 64.66, 65.10, 65.12, 66.07, 89.33, 115.14, 115.23, 115.86, 115.95, 118.39, 118.84, 126.96, 127.15, 128.13, 128.67, 128.76, 128.96, 130.47, 130.75, 131.16, 131.37, 132.02, 132.65, 132.75, 133.83, 134.72, 135.03, 139.03, 139.16, 142.35, 143.05, 143.16, 143.43, 144.90, 144.91, 146.81, 147.01, 156.35, 156.46, 157.30, 167.19, and 167.22. ^195^Pt NMR (86 MHz, acetone-d_6_): δ –2698. Elemental Anal. Calc. for C_30_H_30_Cl_3_KO_3_Pt: C, 46.25; H, 3.88; N, 0.00. Found: C, 46.00; H, 4.14; N, 0.00. ESI-HR-MS (*m*/*z*): calculated for C_30_H_30_Cl_3_O_3_Pt [M – K]^−^: 739.0908, found 739.0897.

Potassium trichlorido[η^2^-(hex-5-en-1-yl (E)-3-(4-((E/Z)-1-(4-hydroxyphenyl)-2-phenylbut-1-en-1-yl)phenyl)acrylate)]platinate(II) (**GW7604-Hex-PtCl**_**3**_). From Zeise’s salt (81 mg, 0.220 mmol) and **GW7604-Hex** (109 mg, 0.240 mmol), dissolved in 5 mL of degassed and dry EtOH. Yellow solid. Yield: 92 mg, 0.120 mmol, 56%. Purity calculated by HPLC (peak area): > 95%. ^1^H NMR (400 MHz, acetone-*d*_*6*_, *E/Z* mixture): δ 0.92 (2 × t, *J* = 7.4 Hz, 3H, CH_2_CH_3_), 1.51–2.02 (m, –CH_2_CH_2_–, 4H), 2.11–2.29 (m, –OCH_2_–, 2H), 2.50 (2 × q, *J* = 7.4 Hz, 18.8 Hz, 2H, CH_2_CH_3_), 3.98–4.33 (m, 4H, –OCH_2_– + C=CH_2_), 4.82–5.13 (*J_H–Pt_* = 66 Hz, 1H, –CH=C), 6.42 (d, *J* = 16.0 Hz, 0.5H, CH=CHCO, *E* isomer), 6.51 (d, *J* = 8.6 Hz, 1H, Ar–H, *Z* isomer), 6.59 (d, *J* = 16.0 Hz, 0.5H, CH=CHCO, *Z* isomer), 6.72 (d, *J* = 8.6 Hz, 1H, Ar–H, *Z* isomer), 6.87 (d, *J* = 8.6 Hz, 1H, Ar–H, *E* isomer), 6.94 (d, *J* = 8.3 Hz, 1H, Ar–H, *E* isomer), 7.06–7.23 (m, 6H, Ar–H), 7.27–7.41 (m, 2H, Ar–H, *E + Z* isomer), 7.54 (d, *J* = 16.0 Hz, 0.5H, CH=CHCO, *E* isomer), 7.69–7.79 (m, 1.5H, Ar–H, *Z* isomer + CH=CHCO, *E* isomer), 8.13 (s, 0.4H, OH, *Z* isomer), and 8.38 (s, 0.6H, OH, *E* isomer). ^13^C NMR (101 MHz, acetone-*d*_*6*_, *E/Z* mixture) δ 13.72, 13.77, 26.32, 26.38, 29.16, 29.19, 33.82, 33.84, 64.80, 64.90, 64.94, 66.08, 89.93, 115.14, 115.23, 115.86, 115.95, 118.43, 118.88, 126.96, 127.15, 128.15, 128.67, 128.76, 128.99, 130.47, 130.74, 131.37, 132.02, 132.65, 132.75, 133.82, 134.72, 135.03, 139.04, 139.17, 142.35, 143.05, 143.16, 143.42, 144.83, 144.85, 146.80, 146.99, 156.46, 157.30, 167.19, and 167.22. ^195^Pt NMR (86 MHz, acetone-*d*_*6*_): δ –2691. Elemental Anal. Calc. for C_31_H_32_Cl_3_KO_3_Pt: C, 46.95; H, 4.07; N, 0.00. Found: C, 47.05; H, 4.31; N, 0.00. ESI-HR-MS (*m*/*z*): calculated for [C_31_H_32_Cl_3_O_3_Pt]^−^ [M – K]^−^: 753.1065, found 753.1051.

### Stability Investigations of **GW7604-Pent-PtCl_3_** in Full Cell Culture Medium by HPLC and ESI-HR-MS

A modified procedure was applied to determine the influence of full cell culture medium on metal complexes.^70^ To 11 mL of complete DMEM medium including 10% FCS, 11 μL of a freshly prepared 3 mM DMF solution of **GW7604-Pent-PtCl**_**3**_ was added. The solution was then incubated for 4 h at 37 °C in the dark. Proteins were precipitated upon addition of 33 mL of ice-cold MeOH, and the mixture was stored for 2 h at −20 °C. The supernatant was then separated by centrifugation (3000 rpm), collected, and dried by lyophilization. The lyophilizate was extracted three times with 5 mL of MeOH and finally dried under reduced pressure. The residue was taken up in 1 mL of HPLC-grade MeOH, and 30 μL thereof was analyzed by HPLC and ESI-HR-MS. Graphs of HPLC chromatograms were prepared using OriginPro 2016 (Northampton, MA, USA). ESI-HR-MS data analysis was carried out with MestreNova v14.

### Determination of Cellular/Nuclear Uptake *via* HR-CS-AAS

After thawing of the cell/nuclei pellets, they were resuspended in ultrapure water (Siemens LaboStar, Günzburg, Germany; 300 μL) and lyzed with a sonotrode (Bandelin Sonoplus, Berlin, Germany; parameters 20 s, 9 cycles, 80% power). Graphite furnace (GF) AAS was used to quantify the lysates concerning their content of platinum (λ = 265.9450 nm). For this purpose, a contrAA 700 High-Resolution-Continous Source AAS (Analytik Jena, Jena, Germany) was employed, while atomization took place by electrothermal heating of graphite tubes with an inserted platform (Analytik Jena, Jena, Germany). A time–temperature program reported in the literature^25^ was followed with minor adjustments. The liquid samples kept in 0.5 mL polystyrene vials (Gesellschaft für Analysentechnik, Salzwedel, Germany) were injected directly into the pyrolytically coated tubes using an MPE 60 autosampler (Analytik Jena, Jena, Germany). Throughout the measurements, argon (Alphagaz 1, 99.999%, Air Liquide, Düsseldorf, Germany) served as the purge and protective gas. The spectrometer was monitored with the ASpect CS software, version 2.3.1.0 (Analytik Jena, Jena, Germany). For all runs, the mean integrated absorbance was determined after three injections of each standard or sample. Calibration was performed by an appropriate dilution of a solution of Cisplatin (Sigma-Aldrich, Taufkirchen, Germany) obtaining five standards (2–10 μg/L, R^2^ ≥ 0.982). Each cell/nuclei lysate was quantified concerning its protein content following the method of Bradford^71^ in order to express the uptake as the amount of compound (pmol) in the lysate relative to its protein mass (mg). Therefore, solutions of Albumin fraction V (Carl Roth, Karlsruhe, Germany) diluted with ultrapure water were adduced for the calibration (six standards, 50–300 μg/mL, *R*^2^ ≥ 0.996). A volume of 20 μL of the Albumin standards or the lysates was added to a 96-well plate (Sarstedt, Nürmbrecht, Germany) and supplemented with 200 μL of Bradford’s reagent (Roti-Nanoquant, Carl Roth, Karlsruhe, Germany; diluted 5-fold with ultrapure water). All standards and lysates were measured in duplicate. After incubating for 5 min at rt, the absorbance (λ = 595 nm) was read on a microplate reader (Tecan Safire 2, Männedorf, Switzerland). The cellular/nuclear uptake (pmol complex/mg protein) is given as the mean ± SD of 2 independent experiments.

### ESI-HR-MS Measurements of GW7604-Pent-PtCl_3_ Incubated with 5′-Guanosine Monophosphate

**GW7604-Pent-PtCl**_**3**_ dissolved in MeOH was combined with an aqueous solution of 5′-GMP (disodium salt) to get an [80/20 (*v*/*v*)] solution with a complex concentration of 1 mM and a complex/5′-GMP proportion of 1/1.1. The mixture was diluted %1/10 (*v*/*v*)] with MeOH/water [80/20 (*v*/*v*)], reaching a final complex concentration of 100 μM, and ESI-HR-MS spectra were recorded after 5 min and 24 h. The obtained spectra were analyzed with MestreNova v14.

### Biological Methods

#### Cell Lines

The human hormone-dependent breast cancer cell line MCF-7 was kindly provided by the Department of Gynecology, Medical University Innsbruck, Austria. The hormone-independent breast cancer cell line SKBr3 and the human embryonic kidney cells TSA-201 were obtained from the cell line service (CLS, Eppelheim, Germany). The cells were maintained as monolayer cultures. DMEM without phenol red, with Glucose (4.5 g/L) (GE Healthcare, Pasching, Austria), supplemented with 10% FCS and 1% Pyruvate (GE Healthcare), Penicillin (100 U mL^–1^), and Streptomycin (100 μg mL^–1^), was used for cultivation. The cells were cultivated in a humidified atmosphere (5% CO_2_/95% air) at 37 °C and passaged twice a week. They were authenticated by the short tandem repeat typing method and routinely monitored for mycoplasma contamination (VenorGeM mycoplasma detection kit, Minerva Biolabs GmbH, Berlin Germany).

#### Sample Preparation for Cellular Uptake Studies

For cellular uptake studies, 1.0 × 10^6^ of MCF-7 and SKBr3 cells in their exponential growing phase were seeded into 25 cm^2^ cell culture flasks and incubated for 24 h under a humidified atmosphere (5% CO_2_/95% air) at 37 °C. Stock solutions of the platinum complexes (**GW7604-Pent-PtCl**_**3**_, Cisplatin) in DMF were freshly prepared and diluted with complete DMEM containing 10% FCS to the desired concentration [final DMF concentration of 0.1% (*v*/*v*), final complex concentration of 20 μM]. The medium in the cell culture flasks was replaced with 3 mL of fresh cell culture medium containing the compounds, and the flasks were incubated under a humidified atmosphere at 37 °C for 3 min, 0.5, 1, 2, 4, and 8 h. Cell pellets were prepared by removing the medium, washing two times with phosphate-buffered saline (PBS), and detaching the cells with Accutase (GE Healthcare, BioSciences, Chicago, IL, USA). Subsequently, cells were pelleted by centrifugation (4 °C, 380*g*, 3 min). The pellets were twice resuspended, washed with PBS, centrifuged, and then stored at −20 °C until further analysis.

#### Sample Preparation for Nuclear Uptake Studies

For nuclear uptake studies, 1.0 × 10^6^ of MCF-7 and SKBr3 cells in their exponential growing phase were seeded into 25 cm^2^ cell culture flasks and incubated for 24 h under a humidified atmosphere (5% CO_2_/95% air) at 37 °C. Stock solutions of the platinum complexes (**GW7604-Pent-PtCl**_**3**_, Cisplatin) in DMF were freshly prepared and diluted with complete DMEM containing 10% FCS to the desired concentration [final DMF concentration of 0.1% (*v*/*v*), final complex concentration of 20 μM]. The medium in the cell culture flasks was replaced with 3 mL of fresh cell culture medium containing the compounds, and the flasks were incubated under a humidified atmosphere (5% CO_2_/95% air) at 37 °C for 24 h. Nuclei pellets were prepared by removing the medium, washing two times with PBS, and detaching the cells with Accutase. Subsequently, cells were pelleted by centrifugation (4 °C, 380*g*, 3 min). The pellets were twice resuspended, washed with PBS, and centrifuged. Subsequently, the pellets were resuspended in 1 mL of a hypertonic TRIS–HCl buffer (TRIS: tris(hydroxymethyl)aminomethane), and the nuclei were isolated using a dounce homogenizer (Kimble Chase Life Sciences, Meiningen, Germany). The outer cell membrane was lysed (30 strokes), and the lysate was centrifuged (4 °C, 25000*g*, 15 min) to separate the nuclei from the other cellular components, washed with PBS, centrifuged again, and then stored at −20 °C until further analysis.

#### Real-Time Live Confocal Microscopy

To investigate the cellular distribution of **GW7604-Pent-PtCl**_**3**_ and its ligand **GW7604-Pent**, the exponentially growing cells were seeded at a density of 1.0 × 10^4^ (MCF-7) and 1.5 × 10^4^ (SKBr3) cells/cavity into a chambered coverslip (μ-slide 8 well, Ibidi, Gräfelfing, Germany) followed by 24 h of incubation under a humidified atmosphere (5% CO_2_/95% air) at 37 °C. Stock solutions were freshly prepared in DMF and diluted in complete DMEM supplemented with 10% FCS to reach a final concentration of 20 μM. After addition of the compounds, the cells were incubated for 5 min, 4, 24, 48, and 72 h. Subsequently, cells were washed with PBS and visualized by real-time live confocal microscopy using an inverted microscope (Zeiss Observer Z1, Zeiss, Oberkochen, Germany) in arrangement with a spinning disc confocal system (UltraVIEW VoX, PerkinElmer, Waltham, MA, USA).

#### Western Blot Analysis

The exponentially growing cells were seeded at a density of 2.5 × 10^6^ (MCF-7) and 3.0 × 10^6^ (SKBr3) cells into 75 cm^2^ flasks. The cells were incubated for 24 h in a humidified atmosphere (5% CO_2_/95% air) at 37 °C. For downregulation experiments, a stock solution of **GW7604-Pent-PtCl**_**3**_ in DMF was freshly prepared and diluted with DMEM containing 10% FCS to the desired concentration [final DMF concentration of 0.1% (*v*/*v*), final complex concentration of 20 μM]. The medium in the flasks was replaced with fresh cell culture medium containing the compound, and the flasks were incubated in a humidified atmosphere at 37 °C for 24 h. Afterward, cells were harvested (cell scratcher), and samples were lysed using a modified radio immunoprecipitation assay buffer [50 mM of TRIS (pH = 8.0), 150 mM NaCl, 0.5% NP-40, 50 mM NaF, 1 mM Na_3_PO_4_, 1 mM phenylmethylsulfonyl fluoride, and protease inhibitors (EDTA-free; Roche, Basel, Switzerland)]. Total protein concentration was determined by employing the Bradford assay as described above, and samples were analyzed on the JESS ProteinSimple (Bio-Techne, Minneapolis, MN, USA) according to the manufacturer’s protocol. The following antibodies were used: GAPDH (no. 2118S, cell signaling), ERα (no. 8644S, cell signaling), and COX-2 (no. 12282, cell signaling). All primary antibodies were diluted with antibody diluents provided by ProteinSimple.

#### Ligand Binding Affinity

A modified LanthaScreen TR-FRET alpha/beta assay was performed, according to the manufacturer’s protocol (Invitrogen, Carlsbad, Germany), to determine the binding affinity to the isolated LBD. The terbium-tagged antiglutathione *S*-transferase (GST) antibody was interchanged with GST antibody conjugated with Alexa 555. Measurements were carried out on a multimode plate reader, Tecan Spark (Tecan, Männedorf, Switzerland). Calculations and visualization were performed with GraphPad Prism 8.0 (GraphPad Software, San Diego, CA, USA).

#### DNA Interference Assay

Two μL (conc. 0.5 μg/μL) of an empty pSport1 (4109 base pairs) plasmid was mixed with 2 μL of each test compound (200 μM in DMF) and diluted with 16 μL of nuclease-free water to reach a final concentration of 20 μM for **GW7604-Pent-PtCl**_**3**_ and 15 μM for Cisplatin. Afterward, this mixture was incubated for 4 h at 37 °C under gentle shaking. Samples were loaded with gel loading buffer on agarose gel [0.5% (*w*/*v*) in 1 × TRIS-acetate-EDTA (TAE) buffer + 0.004% Midori Green Advance]. The following running parameters were used: 95 min at 3 V/cm in 1 × TAE buffer. Images were visualized under UV light (254 nm).

#### Comet Assay

MCF-7 cells were seeded at a density of 1.0 × 10^6^ cells into a 25 cm^2^ flask, followed by 24 h of incubation in a humidified atmosphere (5% CO_2_/95% air) at 37 °C. Freshly prepared stock solutions of **GW7604-Pent-PtCl**_**3**_ and Cisplatin in DMF were used and diluted with the medium to reach a final complex concentration corresponding to their IC_50_ values of 20 μM and 15 μM, respectively. Cells were incubated with the compounds for 48 h. The assay was performed according to the manufacturer’s protocol (Comet assay kit, Abcam, Cambridge, UK). Briefly, after lysis and application to the microscopy slides, electrophoresis was run in an alkaline buffer for 20 min at 2 V/cm. Subsequently, slides were washed with pure deionized water and EtOH/water [7/3 (*v*/*v*)], and stained with Vista Green DNA Dye. Visualization was carried out by real-time live confocal microscopy using an inverted microscope (Zeiss Observer Z1) in arrangement with a spinning disc confocal system (UltraVIEW VoX).

#### COX Inhibition Assay

Inhibition of the isolated COX-1 and COX-2 isoenzymes (each human-recombinant) by the platinum complexes **GW7604-Alk-PtCl_3_** (20 μM; 0.1–20 μM for IC_50_ determination of **GW7604-Pent-PtCl**_**3**_), Zeise’s salt (20 μM), and Celecoxib (5 μM) were evaluated using an enzyme immunoassay (EIA) (COX Inhibitor Screening Assay, Cayman Chemicals) following the manufacturer’s protocol. The incubation time of the compounds with the respective isoenzymes was exactly 10 min. The results are presented as the mean ± SEM of three independent experiments, with two replicates of each experiment. The untreated control was set at 0% inhibition of COX activity. Graphs were drawn using GraphPad Prism 8.0.

#### Analysis of Cell Growth Inhibition

The exponentially growing cells were seeded at a density of 1750 (MCF-7), 2000 (SKBr3), and 1250 (TSA-201) cells/well into clear flat-bottom 96-well plates in triplicate. Following 24 h of incubation at 37 °C in a humidified atmosphere (5% CO_2_/95% air), the compounds [freshly prepared stock solutions in DMF, diluted with DMEM to the desired concentration (final DMF concentration of 0.1% (*v*/*v*))] were added to reach the indicated concentrations. After another 72 h of incubation, the cellular metabolic activity was measured employing an MTT assay. By adding predissolved MTT reagent in PBS at a final concentration of 0.5 mg/mL, the metabolic activity can be determined *via* an enzymatic conversion to a purple formazan salt. After 3 h, the medium was aspirated, and 200 μL of DMSO was added to dissolve the formed formazan crystals. The absorbance was measured at 570 and 690 nm (turbidity assessment) using an EnSpire plate reader (PerkinElmer, Waltham, MA, USA). Solvent-treated cells without a compound served as the positive control. Metabolic activity was calculated by the following equation
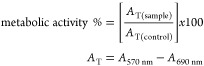


The IC_50_ values were calculated with GraphPad Prism 8.0 using nonlinear regression.

## Supporting Information Available

^1^H, ^13^C, ^1^H/^1^H COSY, and ^1^H/^13^C HSQC NMR spectra of the **GW7604-Alk-PtCl**_**3**_ complexes; conformational description of **GW7604-But-PtCl**_**3**_ isomers; NMR spectra from stability studies in organic solvents; HPLC chromatograms of the **GW7604-Alk-PtCl**_**3**_ complexes; calculated and found isotopic distribution pattern of [GW7604-Pent-Pt(Ala)(CH_3_OH)]^+^; additional ESI-HR-MS data from reactivity studies toward 5′-guanosine monophosphate; and additional biological data for TSA-201 and MCF-7 cells (DOCX)
Molecular formula strings of **GW7604-Alk-PtCl**_**3**_ with IC_50_ values in MCF-7, SKBr3, and TSA-201 cell lines as well as **GW7604-Alk** in MCF-7 cells (CSV)

## Abbreviations

ACN: acetonitrile
ASA: acetylsalicylic acid
bp: base pairs
COX: cyclooxygenase
DACH: 1,2-diaminocyclohexane
DCM: dichloromethane
DMAP: 4-(dimethylamino)pyridine
DMEM: Dulbecco’s modified Eagle’s medium
DMF: dimethylformamide
DMSO: dimethyl sulfoxide
DNA: deoxyribonucleic acid
E2: estradiol
EDC: 1-ethyl-3-(3-(dimethylamino)propyl)carbodiimide
EIA: enzyme immunoassay
ELISA: enzyme-linked immunosorbent assay
EtOH: ethanol
ER: estrogen receptor
ESI-HR-MS: electrospray ionization high-resolution mass spectrometry
eq: equivalents
en: ethylene
FCS: fetal calf serum
GF: graphite furnace
5′-GMP: 5′-guanosin monophosphate
GST: antiglutathione *S*-transferase
GVs: giant vesicles
HPLC: high-performance liquid chromatography
HR-CS-AAS: high-resolution continuous source atomic absorption spectrometry
LBDs: ligand-binding domains
LBS: ligand-binding site
MeOH: methanol
mER: membrane-associated estrogen receptor
MTT: 3-(4,5-dimethylthiazol-2-yl)-2,5-diphenyltetrazolium bromide
NMR: nuclear magnetic resonance
PGE2: prostaglandin E2
PBS: phosphate buffered saline
RBA: relative binding affinity
rpm: revolutions per minute
rt: room temperature
SD: standard deviation
SEM: standard error of mean
SERD: selective estrogen receptor downregulator
TAE: TRIS-acetate-EDTA
TBAF: tetra-*n*-butylammonium fluoride
TBDMSCl: *tert*-butyldimethylsilyl chloride
THF: tetrahydrofuran
*t*_R_: retention time
TR-FRET: time-resolved fluorescence energy transfer
TRIS: tris(hydroxymethyl)aminomethane
(*v*/*v*): volume per volume
(*w*/*v*): percent of weight of solution in the total volume of solution
