# Correction to “Development of Cytotoxic GW7604-Zeise’s Salt Conjugates as Multitarget Compounds with Selectivity for Estrogen Receptor-Positive Tumor Cells”

**DOI:** 10.1021/acs.jmedchem.4c00799

**Published:** 2024-04-23

**Authors:** Patricia Grabher, Paul Kapitza, Nikolas Hörmann, Amelie Scherfler, Martin Hermann, Michael Zwerger, Hristo P. Varbanov, Brigitte Kircher, Daniel Baecker, Ronald Gust

Details of the correction:

In Figure S28 of the Supporting Information, the inserted structural formula of the proposed alanine-containing adduct **[GW7604-Pent-Pt(Ala)(CH**_**3**_**OH)]**^**+**^ erroneously covers a peak with *m*/*z* = 752.25 in the calculated spectrum. However, this peak was not included in the found spectrum (Figure S28). An unequivocal assignment to the adduct **[GW7604-Pent-Pt(Ala)(CH**_**3**_**OH)]**^**+**^ is therefore not possible.

Although the main peak with *m*/*z* = 753.34 in the MS spectrum (Figure 4) could correspond to the suspected adduct with *m*/*z* = 753.25 (solely based on the *m*/*z* ratio), the missing platinum isotope distribution pattern, visible in the simulated spectrum (Figure S28), does not allow this assignment. Consequently, the degradation product identified in the protein-free fraction of the cell culture medium is preferably an organic species, whose structure has not yet been elucidated.

**Figure 4:**
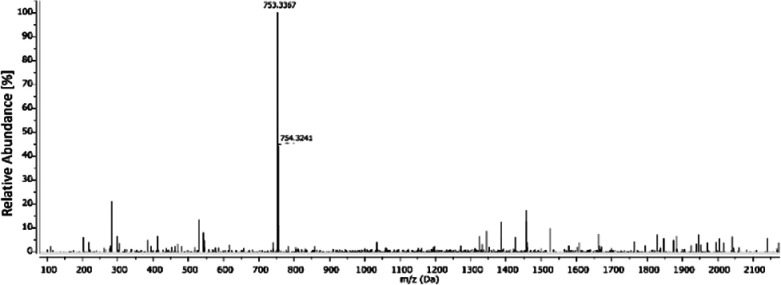
Full ESI-MS (positive mode) spectrum of the methanolic extract from DMEM (+10% FCS) incubated with **GW7604-Pent-PtCl**_**3**_.

Reaction of **GW7604-Pent-PtCl**_**3**_ with amino acids as discussed using alanine as an example is principally possible and causes also coordinative binding to proteins. During precipitation with methanol, the complexes are thus separated from the free fraction, and the methanol probably also causes the release of the organic species found from the protein-bound complexes. Unfortunately, it is not possible to quantify this reaction on the basis of the MS measurements.

Since this behavior is comparable to that of other platinum complexes, the most important statements of the paper, such as the description of the potential of GW7604-Zeise’s salt conjugates as a new class of cytotoxic drugs, their multitarget effect (e.g., inhibition of COX-2, DNA interaction), and the selective effects on estrogen receptor-positive tumor cells, are not affected.

## Supporting Information Available

NMR spectra, conformational description of isomers, HPLC chromatograms, calculated and found isotopic distribution pattern, ESI-MS data, biological data (PDF)

