# Supplementary material for: Development of Cytotoxic GW7604-Zeise’s Salt Conjugates as Multitarget Compounds with Selectivity for Estrogen Receptor- Positive Tumor Cells
Source: J Med Chem. 2024 Mar 13;67(6):4870–88. doi: 10.1021/acs.jmedchem.3c02454 (PMC10983001; doi:10.1021/acs.jmedchem.3c02454)
Supplement: Supplementary file 1 — jm3c02454_si_001.docx [file jm3c02454_si_001.docx]

Supporting Information for:

**Development of Cytotoxic GW7604-Zeise´s Salt Conjugates as Multitarget Compounds with Selectivity for Estrogen Receptor-Positive Tumor Cells**

Patricia Grabher,^†^ Paul Kapitza,^*,†^ Nikolas Hörmann,^†^ Amelie Scherfler,^†^ Martin Hermann,^#^ Michael Zwerger,^§^ Hristo P. Varbanov,^†^ Brigitte Kircher,^‡,¤^ Daniel Baecker,^*,^ⁱ and Ronald Gust^*,†^

^†^ Department of Pharmaceutical Chemistry, Institute of Pharmacy, Center for Molecular Biosciences Innsbruck, University of Innsbruck, Innrain 80/82, A-6020 Innsbruck.

^#^ Department of Anesthesiology & Critical Care Medicine, Medical University Innsbruck, Anichstrasse 35, A-6020 Innsbruck.

^§^ Department of Pharmacognosy, Institute of Pharmacy, Center for Molecular Biosciences Innsbruck, University of Innsbruck, Innrain 80/82, A-6020 Innsbruck.

^‡^ Department of Internal Medicine V, Haematology & Oncology, Immunobiology and Stem Cell Laboratory, Medical University Innsbruck, Anichstrasse 35, A-6020 Innsbruck.

^¤^ Tyrolean Cancer Research Institute, Innrain 66, A-6020 Innsbruck.

ⁱ Department of Pharmaceutical and Medicinal Chemistry, Institute of Pharmacy, Freie Universität Berlin, Königin-Luise-Straße 2+4, D-14195 Berlin.

Corresponding Authors:

Paul Kapitza ([paul.kapitza@uibk.ac.at](mailto:paul.kapitza@uibk.ac.at))

Daniel Baecker ([d.baecker@fu-berlin.de](mailto:d.baecker@fu-berlin.de))

Ronald Gust ([gust.ronald@gmail.com](mailto:gust.ronald@gmail.com))

Table of Contents

[1. ^1^H, ^13^C, ^1^H/^1^H COSY, and ^1^H/^13^C HSQC NMR Spectra of the **GW7604-Alk-PtCl_3_** Complexes S2](#_Toc157759302)

[2. Conformational Description of **GW7604-But-PtCl_3_** Isomers S10](#_Toc157759303)

[3. NMR Spectra from Stability Studies in Organic Solvents S11](#_Toc157759304)

[4. HPLC Chromatograms of the **GW7604-Alk-PtCl_3_** Complexes S14](#_Toc157759305)

[5. Calculated and Found Isotopic Distribution Pattern for **[GW7604-Pent-Pt(Ala)(CH_3_OH)]^+^** S15](#_Toc157759306)

[6. Additional ESI-HR-MS Data from Reactivity Studies Towards 5´-Guanosin Monophosphate S16](#_Toc157759307)

[7. Additional Biological Data for TSA-201 and MCF-7 Cells S16](#_Toc157759308)

## ^1^H, ^13^C, ^1^H/^1^H COSY, and ^1^H/^13^C HSQC NMR Spectra of the GW7604-Alk-PtCl_3_ Complexes

**Figure S1.** ^1^H NMR (400 MHz) of **GW7604-Prop-PtCl_3_** in acetone*-d_6_*

**Figure S2.** ^13^C NMR (101 MHz) of **GW7604-Prop-PtCl_3_** in acetone*-d_6_.*

**Figure S3.** ^1^H/^13^C HSQC of **GW7604-Prop-PtCl_3_** in acetone-*d_6_*

*
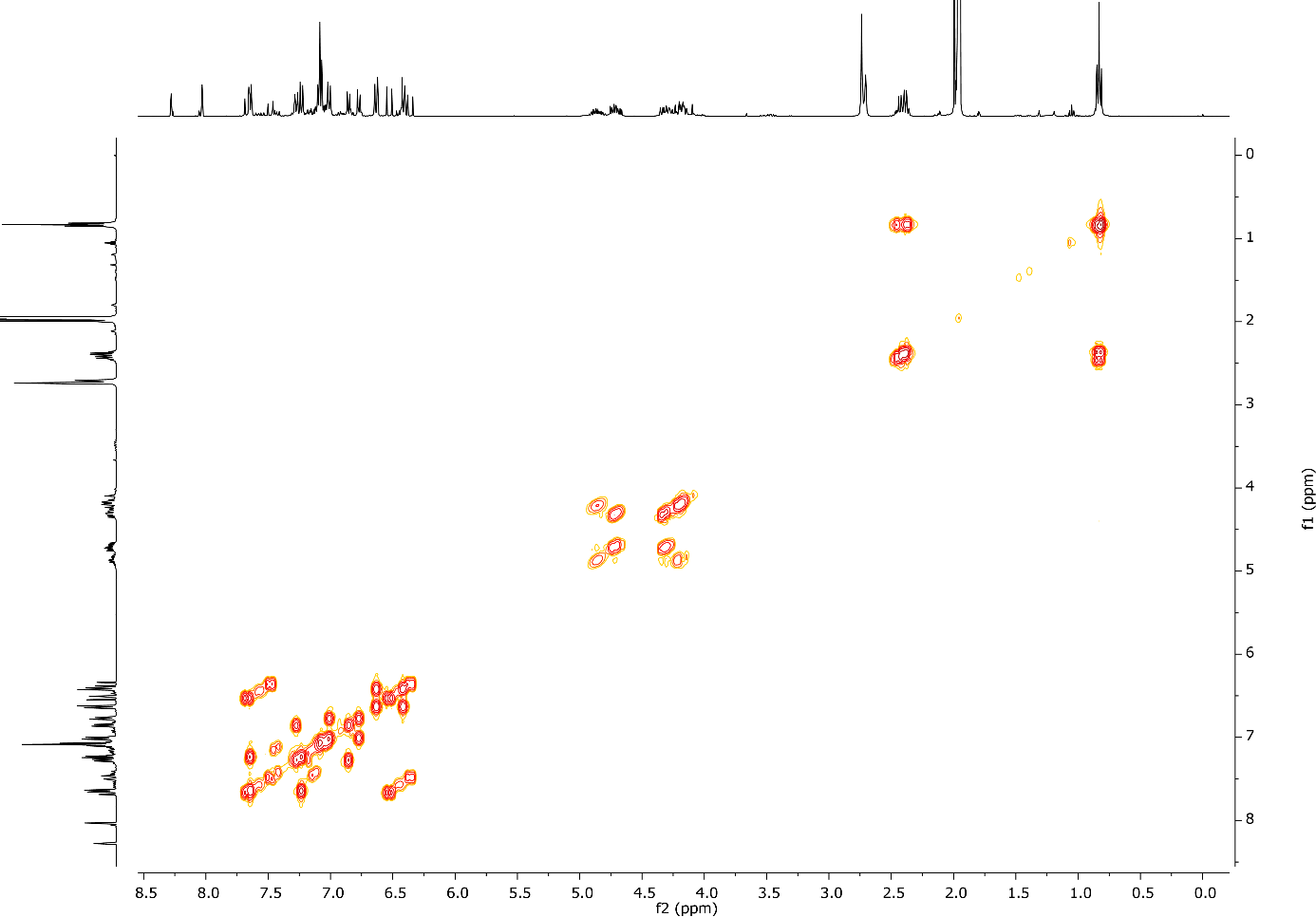
*

**Figure S4.** ^1^H COSY of **GW7604-Prop-PtCl_3_** in acetone-*d_6._*

**Figure S5.** ^1^H NMR (400 MHz) of the **GW7604-But-PtCl_3_** in acetone-*d_6_.*

**Figure S6.** ^13^C NMR (101 MHz) of **GW7604-But-PtCl_3_** in acetone*-d_6_.*

*
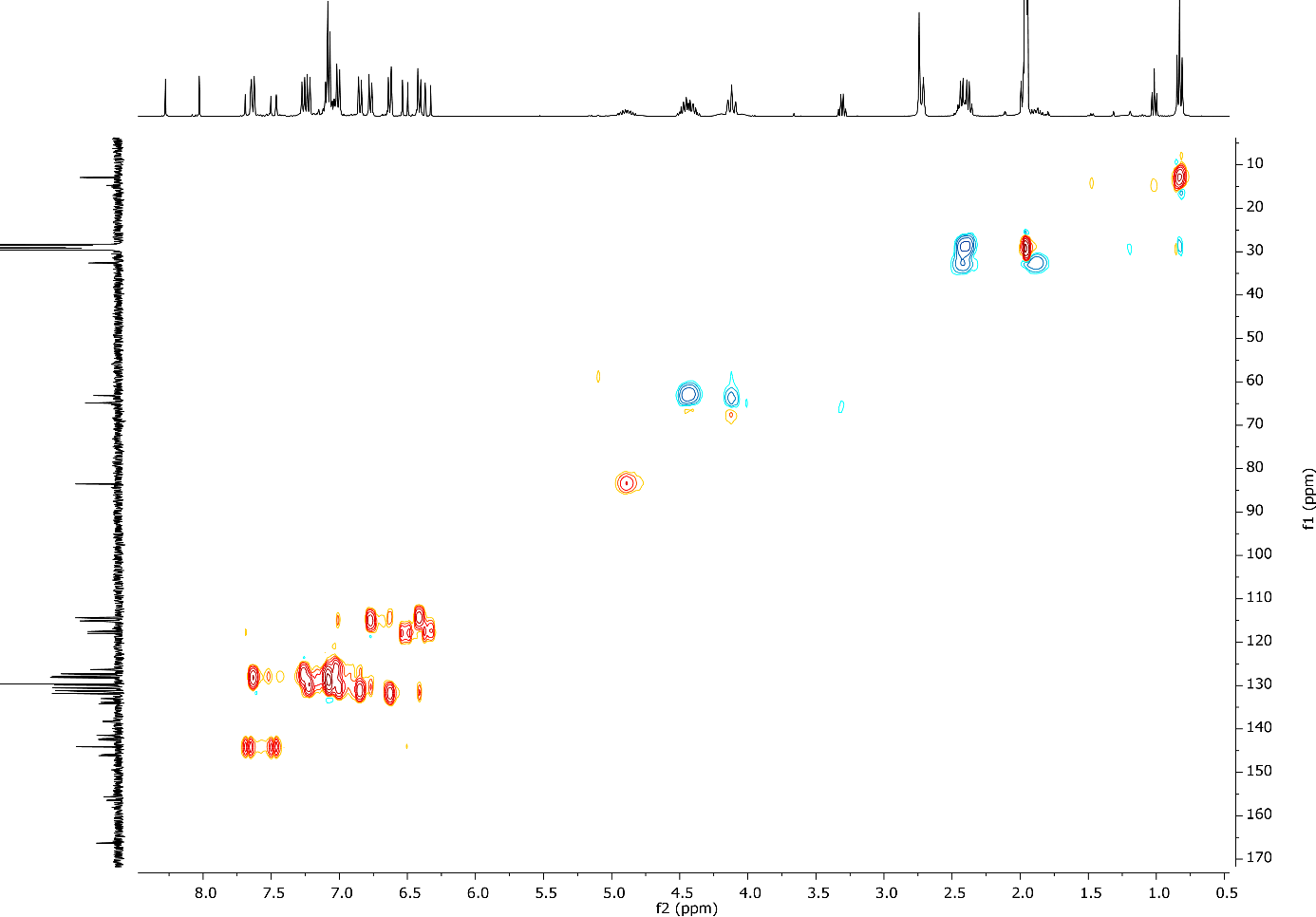
*

**Figure S7.** ^1^H/^13^C HSQC of **GW7604-But-PtCl_3_** in acetone*-d_6_.*

*
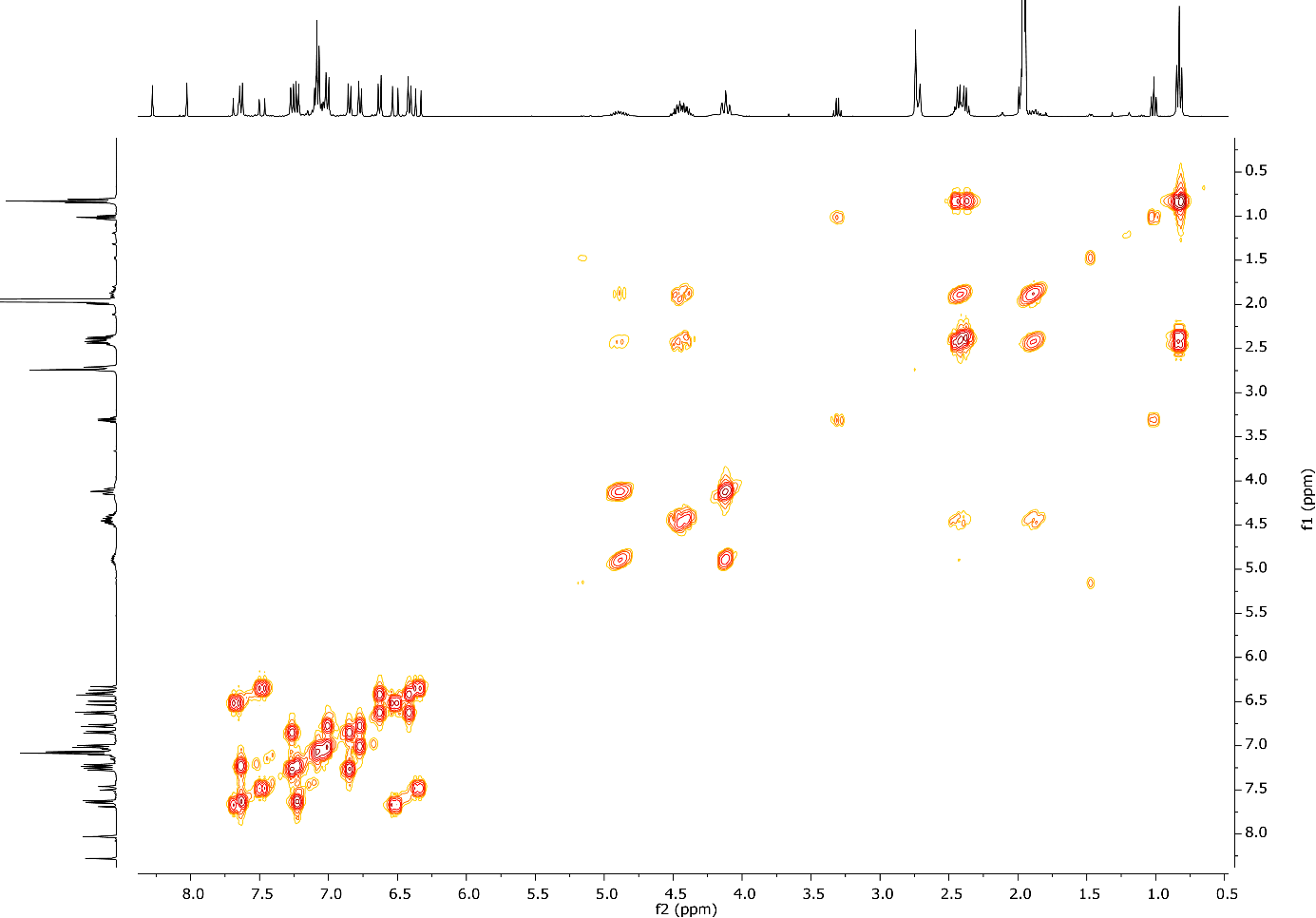
*

**Figure S8.** ^1^H COSY of **GW7604-But-PtCl_3_** in acetone*-d_6._*

**Figure S9.** ^1^H NMR (400 MHz) of **GW7604-Pent-PtCl_3_** in acetone*-d_6_.*

**Figure S10.** ^13^C NMR (101 MHz) of **GW7604-Pent-PtCl_3_** in acetone*-d_6_.*

*
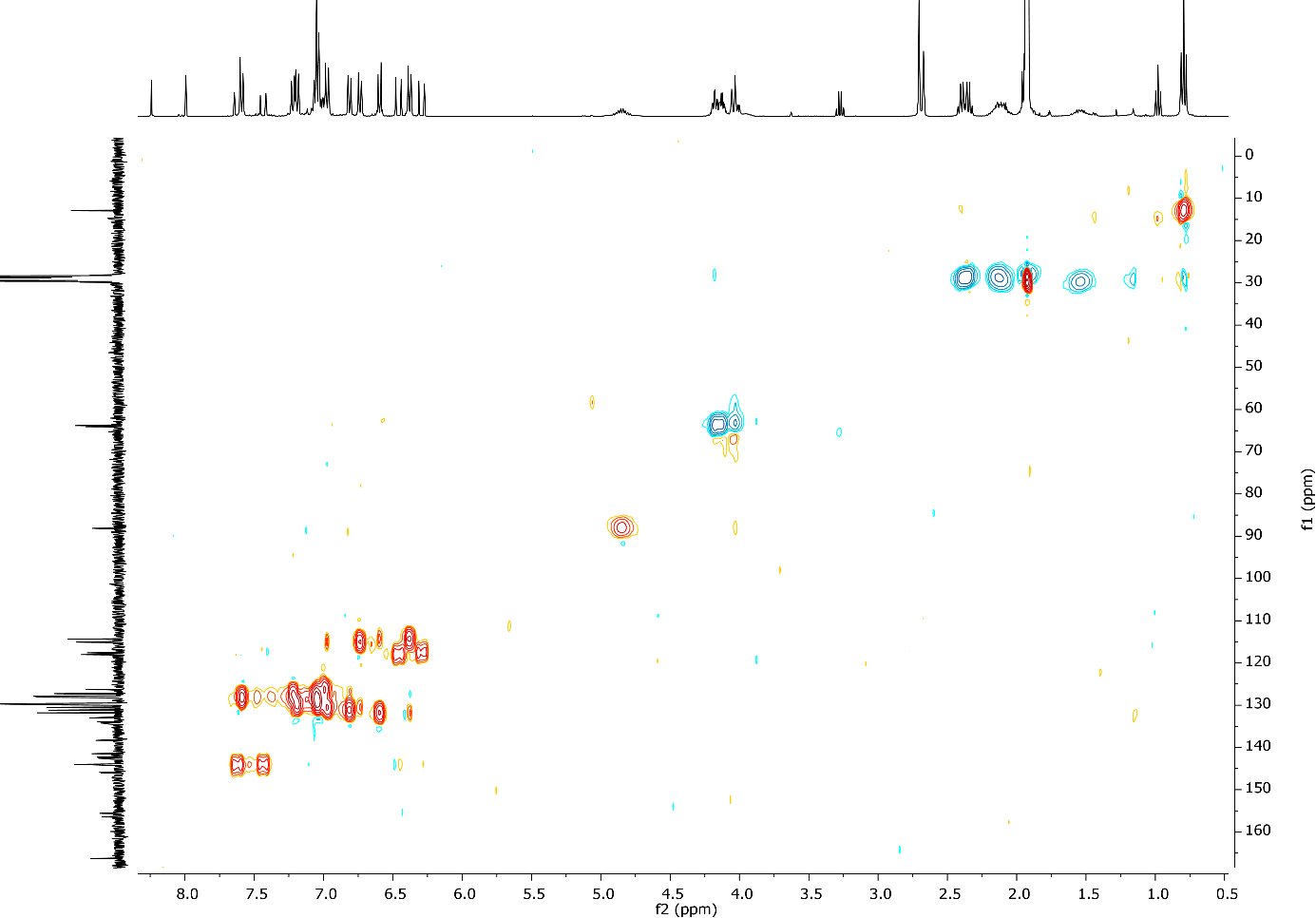
*

**Figure S11**. ^1^H/^13^C HSQC of the **GW7604-Pent-PtCl_3_** in acetone*-d_6._*

*
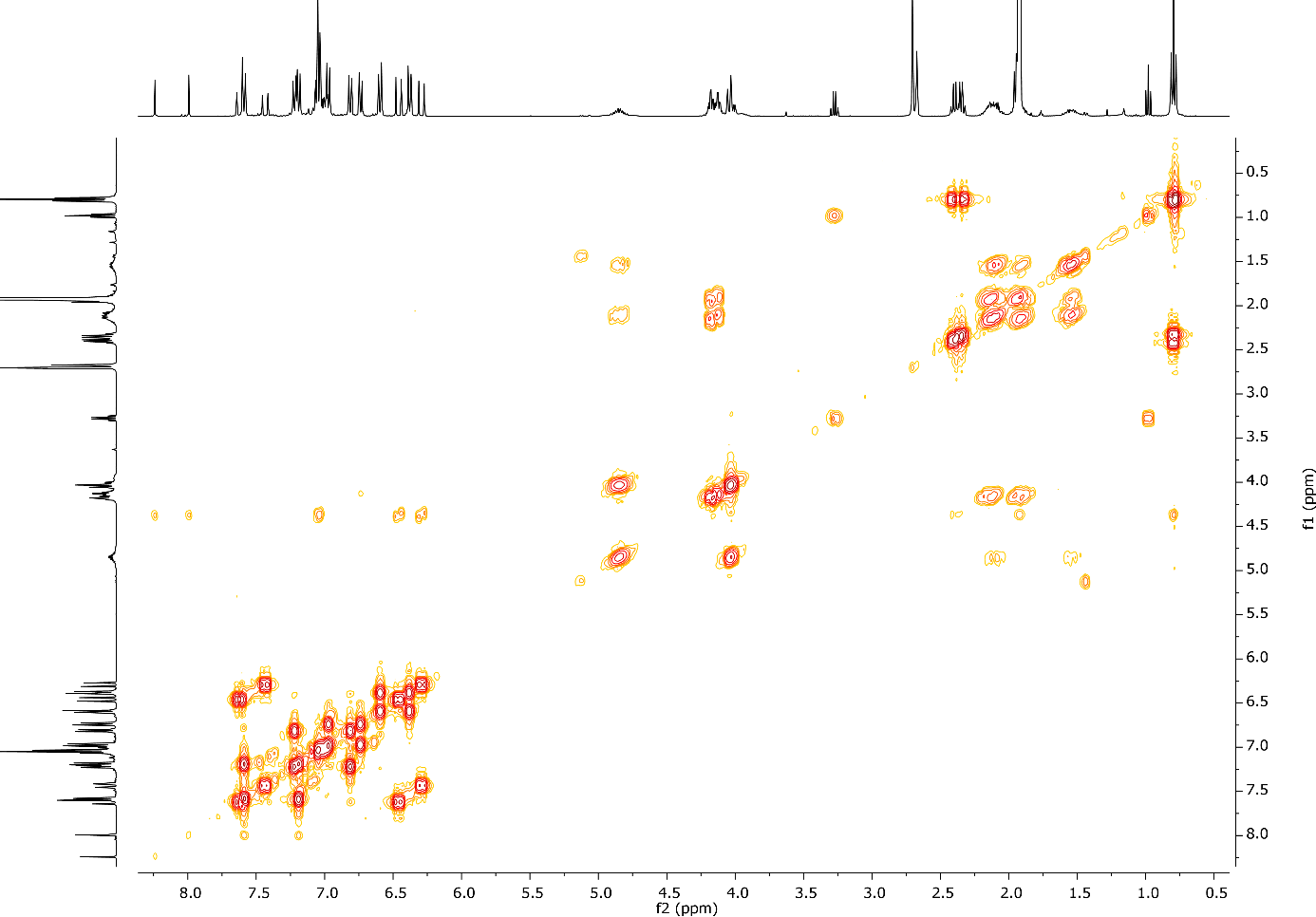
*

**Figure S12.** ^1^H COSY of the **GW7604-Pent-PtCl_3_** in acetone*-d_6._*

**Figure S13.** ^1^H NMR (400 MHz) of **GW7604-Hex-PtCl_3_** in acetone-*d_6_.*

**Figure S14.** ^13^C NMR (101 MHz) of **GW7604-Hex-PtCl_3_** in acetone-*d_6_.*

**Figure S15.** ^1^H/^13^C HSQC of **GW7604-Hex-PtCl_3_** in acetone-*d_6._*

*
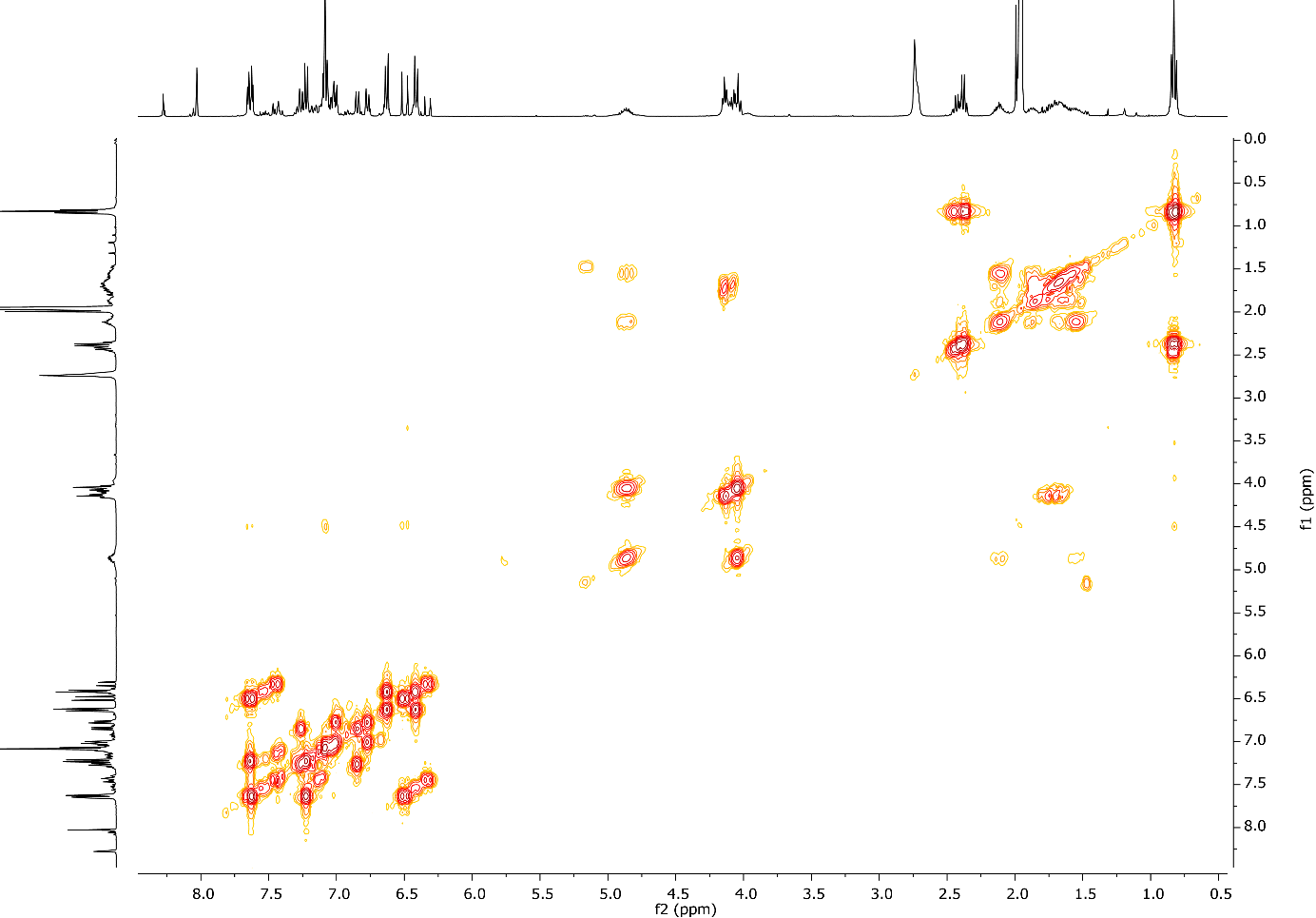
*

**Figure S16.** ^1^H COSY of **GW7604-Hex-PtCl_3_** in acetone-*d_6._*

## Conformational description of GW7604-But-PtCl_3_ Isomers


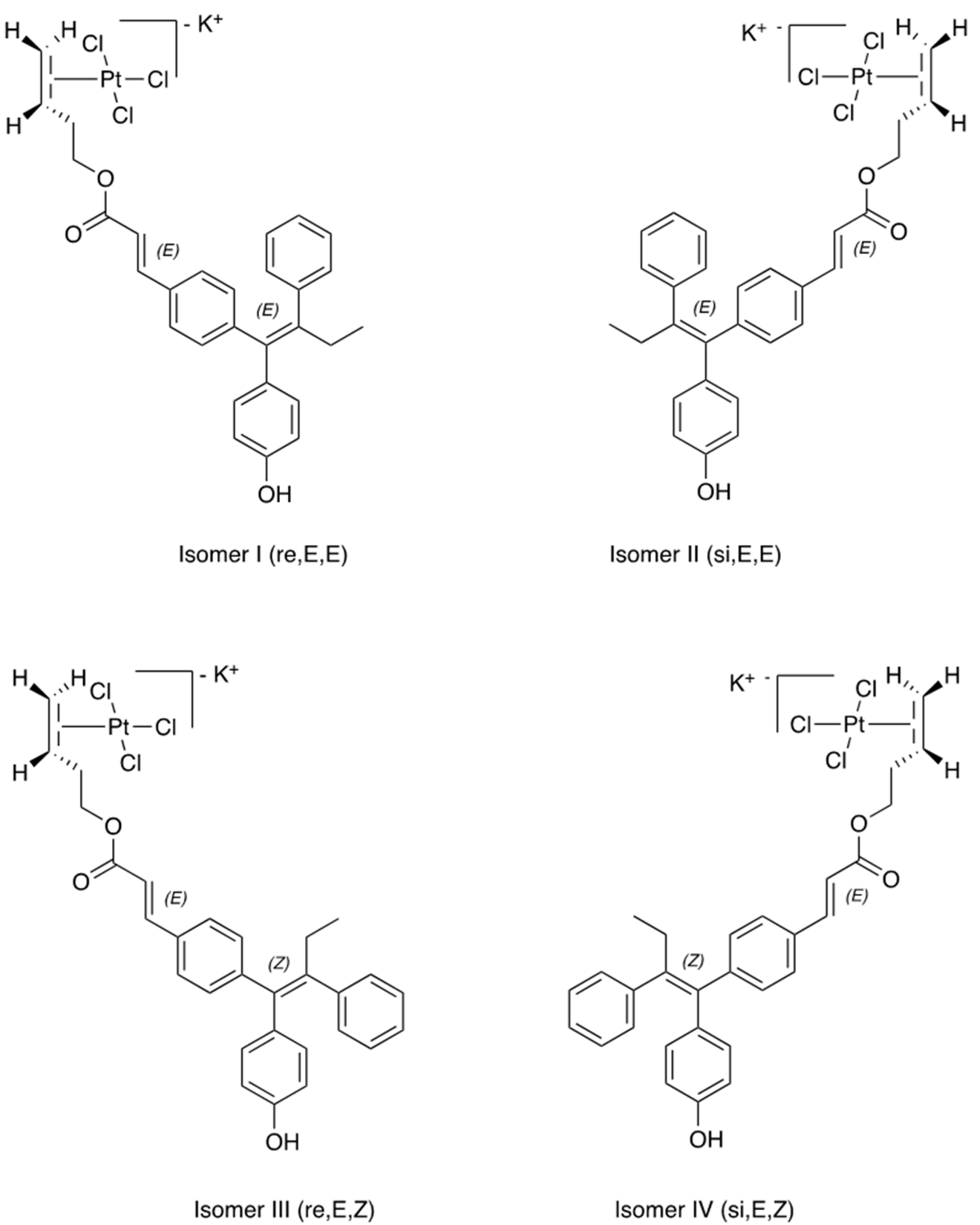


**Figure S17.** Conformational description of **GW7604-But-PtCl_3_** isomers I and II as well as isomers III and IV are enantiomers. The enantiomeric pairs I/II and III/IV are diastereomeric to each other. They cause for instance two sets of Signals in the ^1^H NMR spectrum (Figure 1).

## NMR Spectra from Stability Studies in Organic Solvents


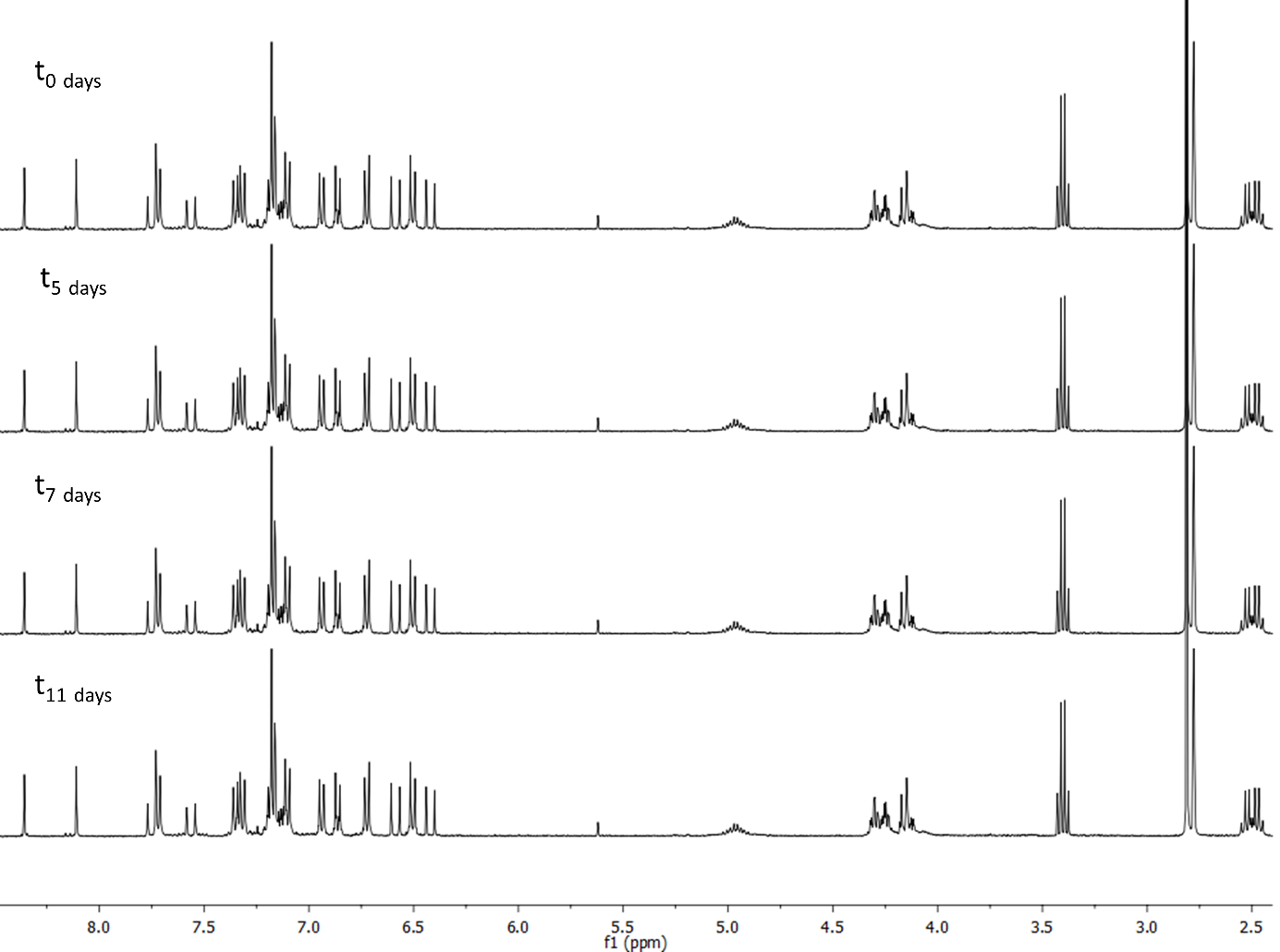


**Figure S18.** ^1^H NMR (400 MHz) of **GW7604-Pent-PtCl_3_** in acetone-d_6_.


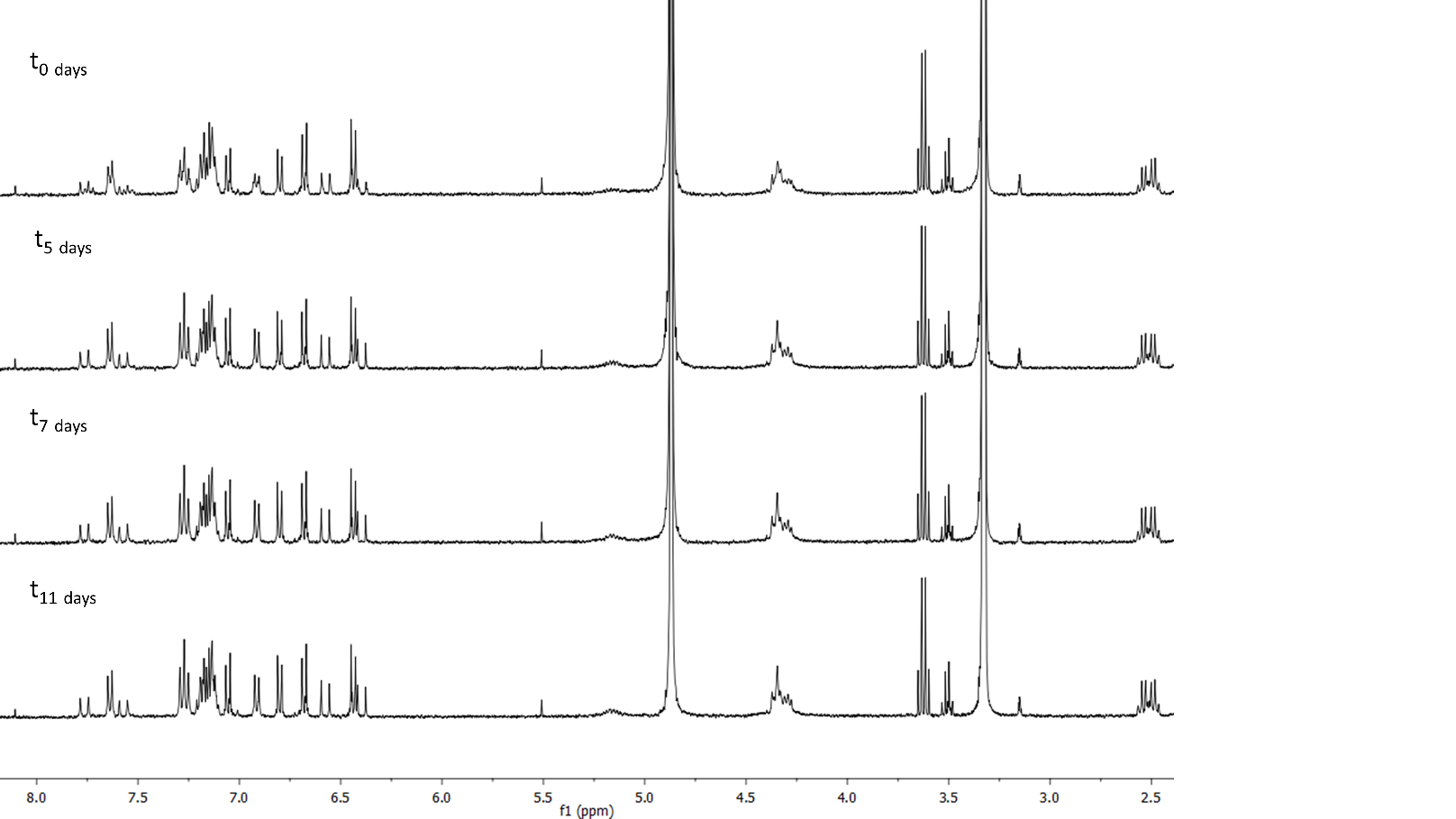


**Figure S19.** ^1^H NMR (400 MHz) of **GW7604-Pent-PtCl_3_** in methanol-*d*_4_*.*


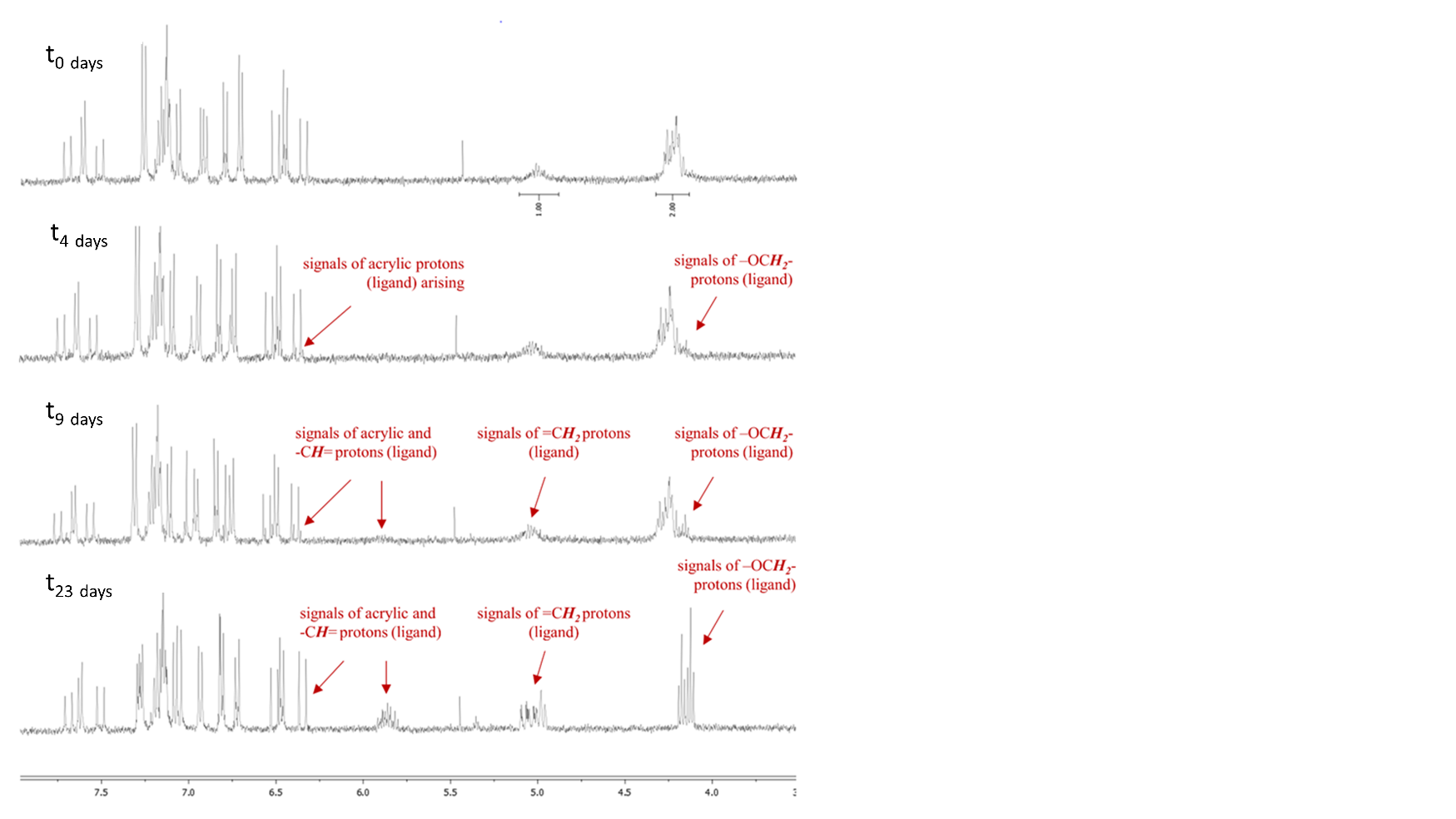


**Figure S20.** ^1^H NMR (400 MHz) of **GW7604-Pent-PtCl_3_** in acetonitrile-*d*_3_*.*


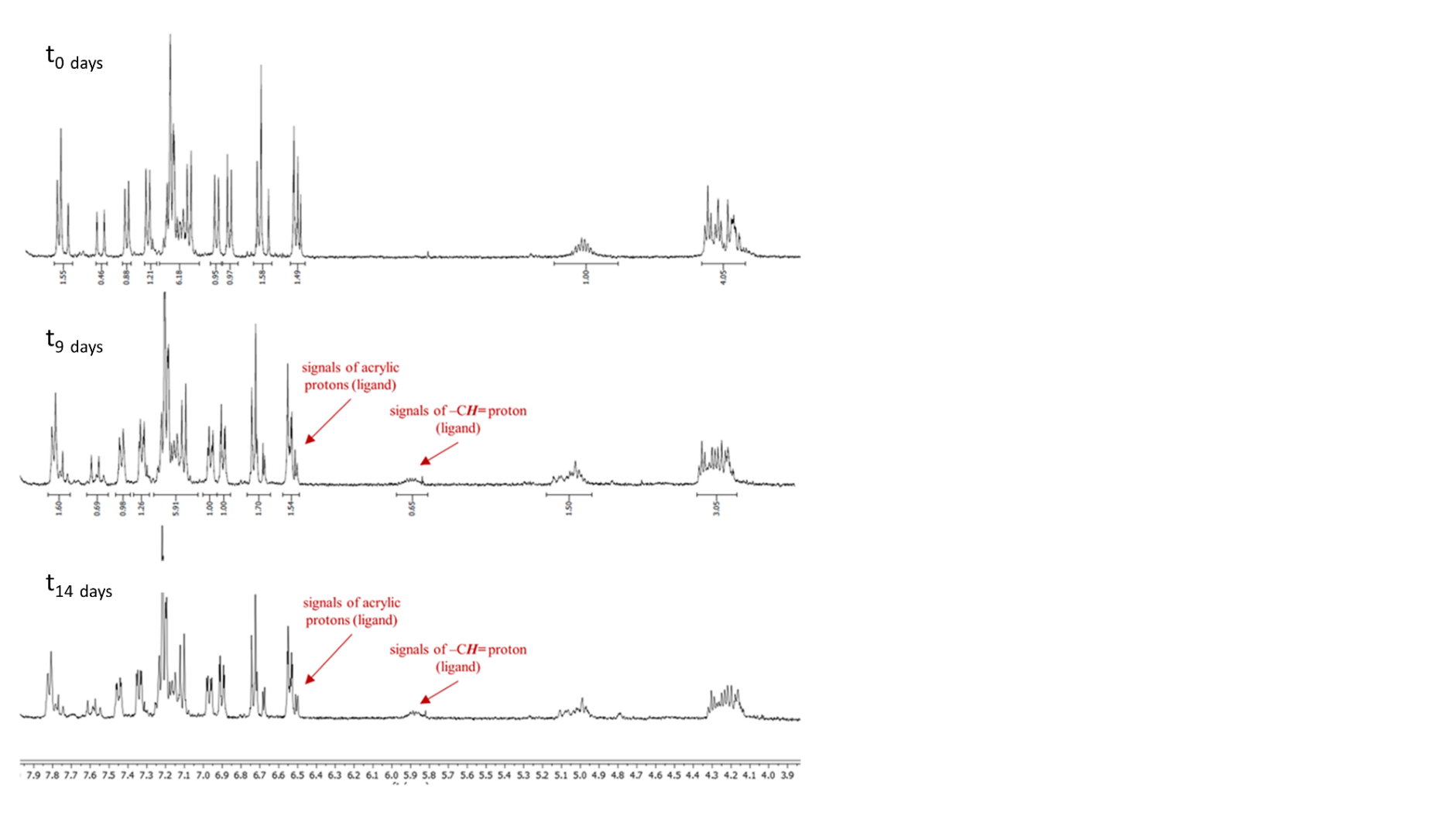


**Figure S21.** ^1^H NMR (400 MHz) of **GW7604-Pent-PtCl_3_** in DMF-d_7._


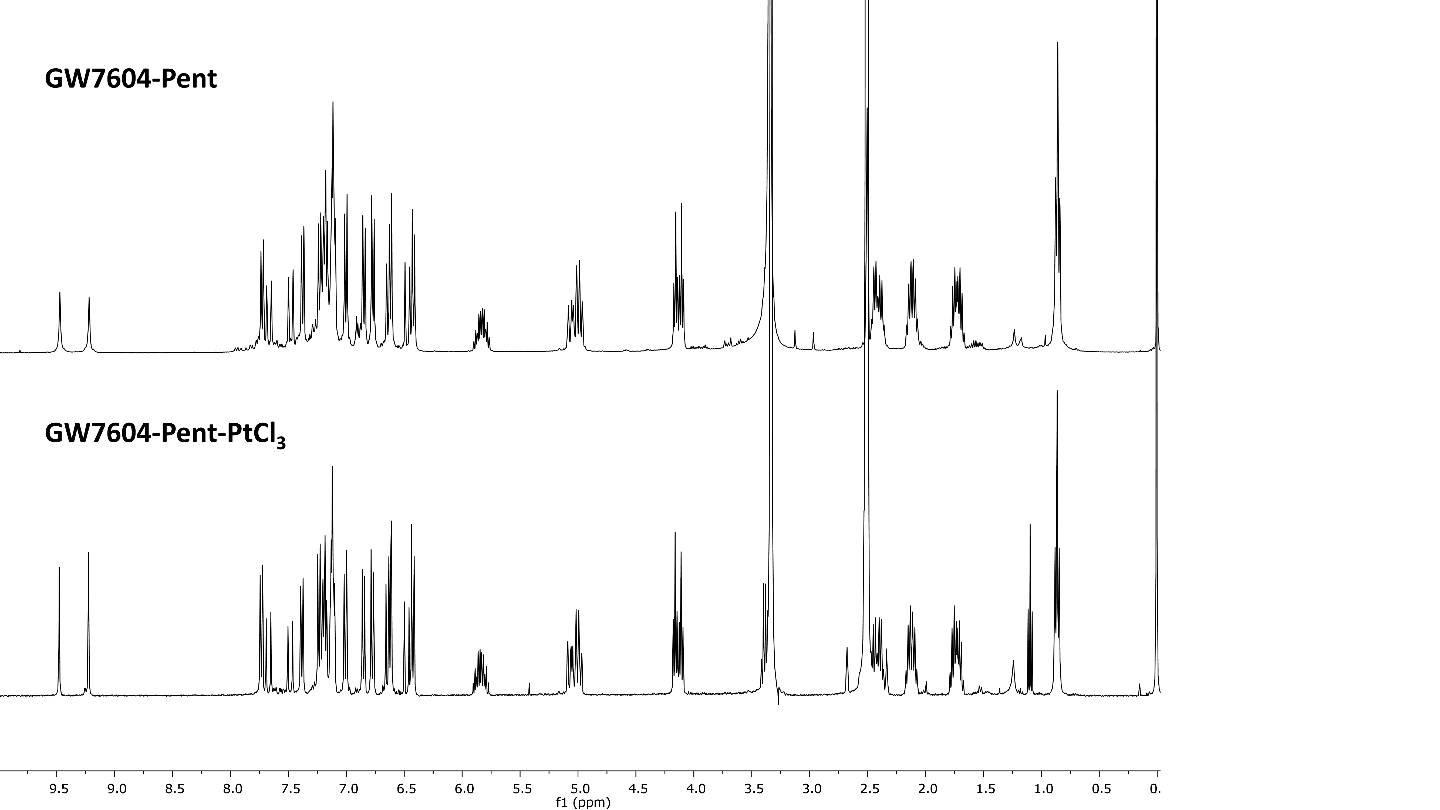


**Figure S22.** ^1^H NMR spectra (400 MHz) of **GW7604-Pent** and **GW7604-Pent-PtCl_3_** recorded in DMSO-*d_6_* after 5 min of incubation at rt. The spectrum of **GW7604-Pent-PtCl_3_** contains traces of diethyl ether.


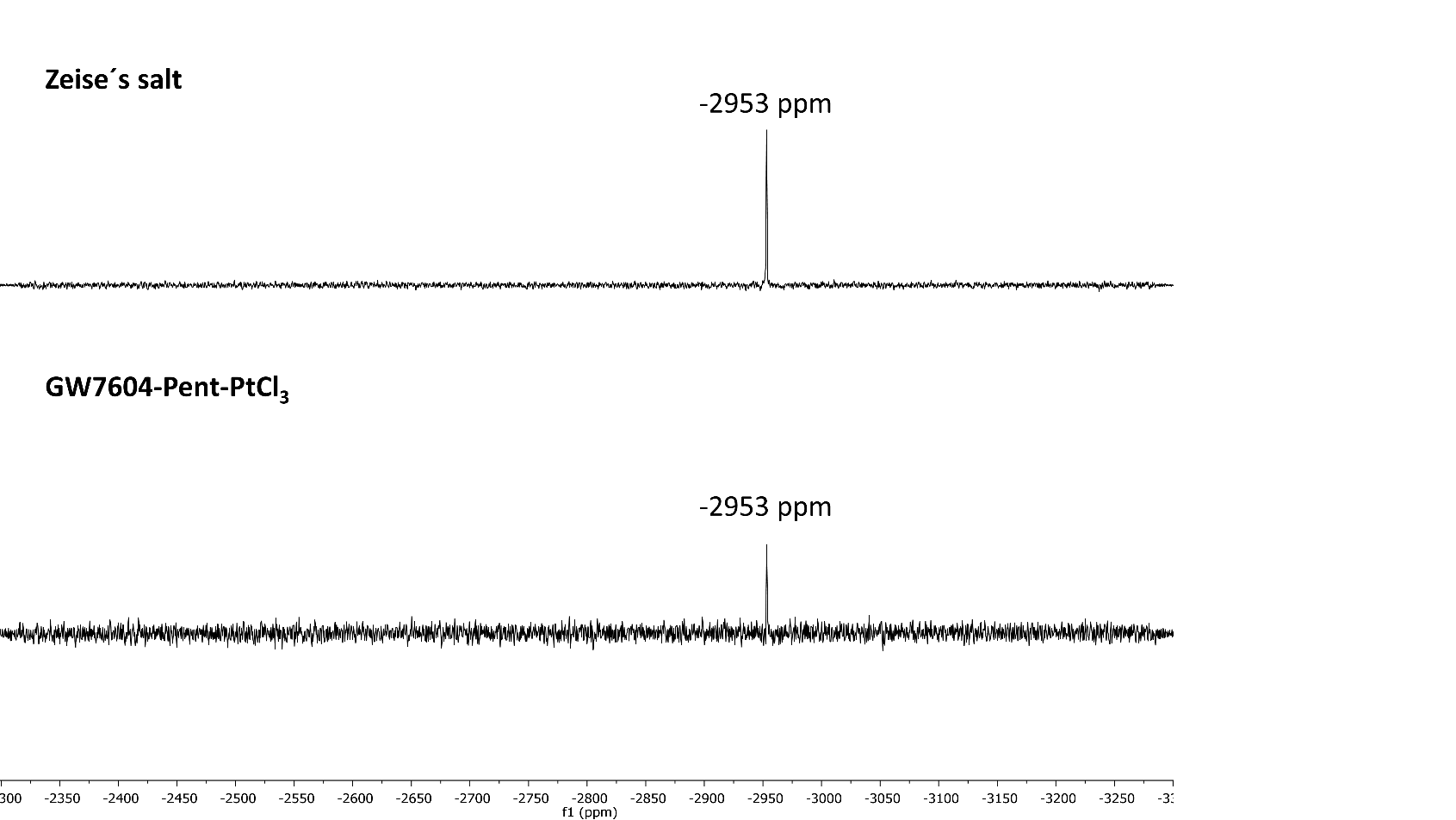


**Figure S23.** ^195^Pt NMR spectra (86 MHz) of **Zeise´s salt** and **GW7604-Pent-PtCl_3_** recorded in DMSO-d_6_ after 24 h of incubation at rt.

## HPLC Chromatograms of the GW7604-Alk-PtCl_3_ Complexes

**
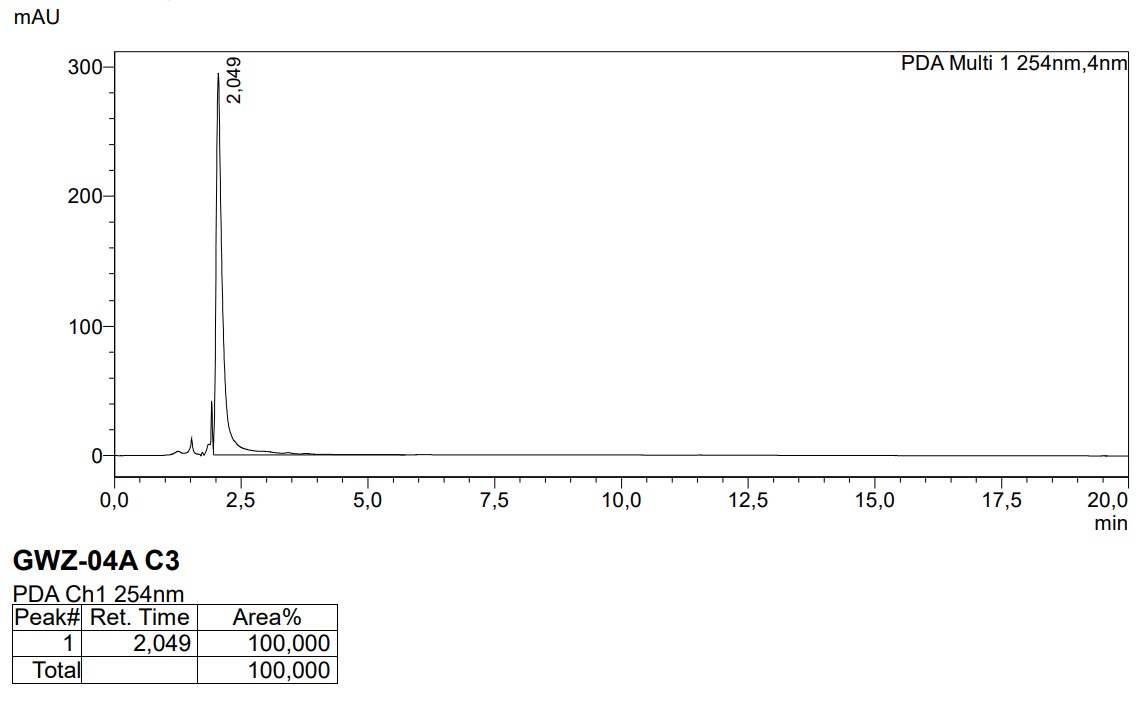
**

**Figure S24.** HPLC chromatogram of **GW7604-Prop-PtCl_3_**.

**
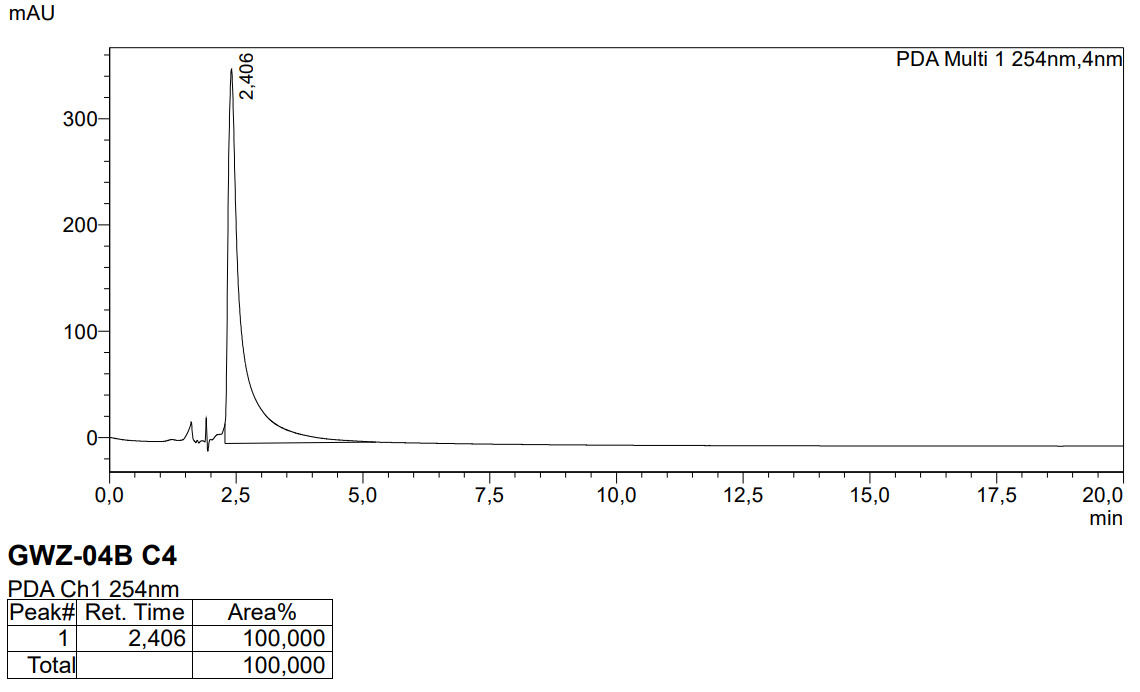
**

**Figure S25.** HPLC chromatogram of **GW7604-But-PtCl_3_**.
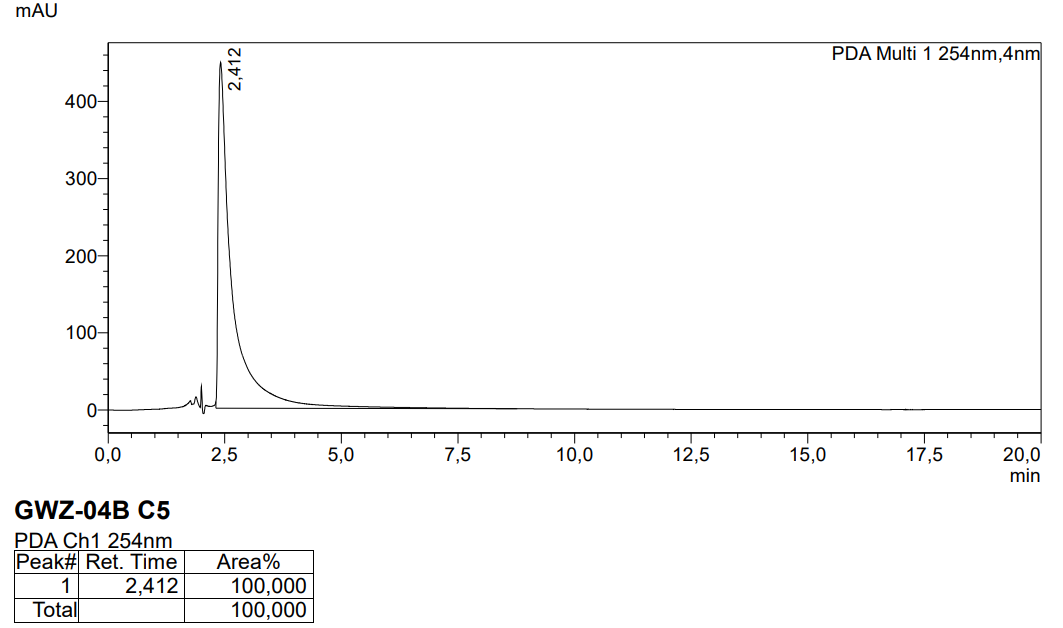


**Figure S26.** HPLC chromatogram of **GW7604-Pent-PtCl_3_**.

**
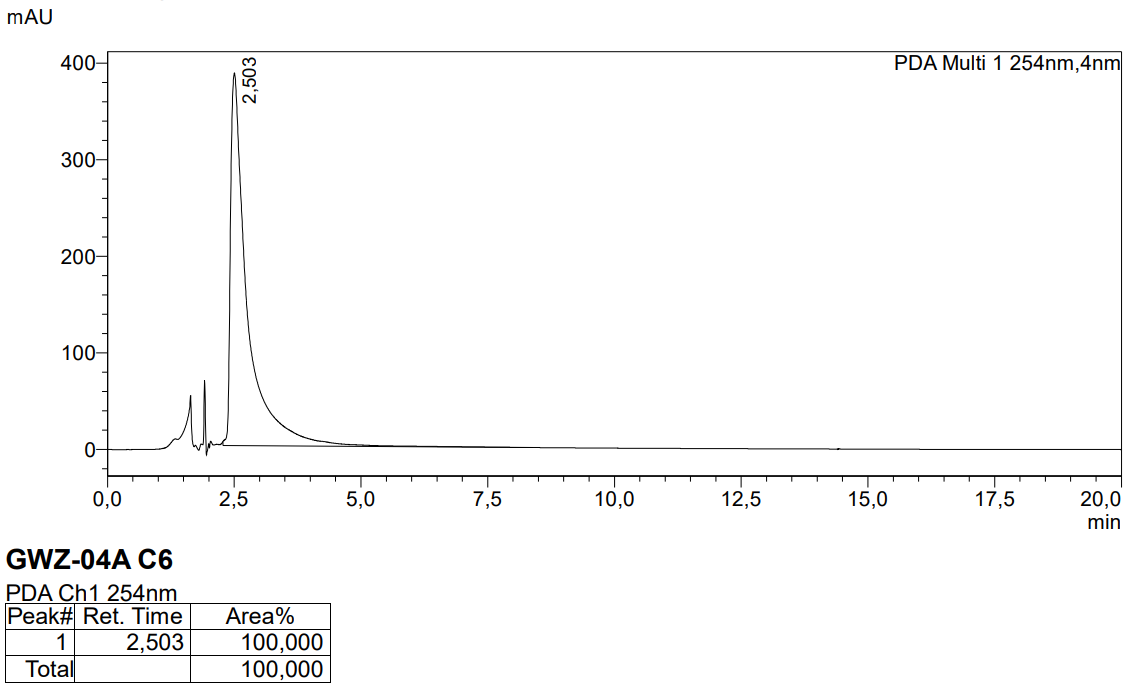
**

**Figure S27.** HPLC chromatogram of **GW7604-Hex-PtCl_3_**.

## ^
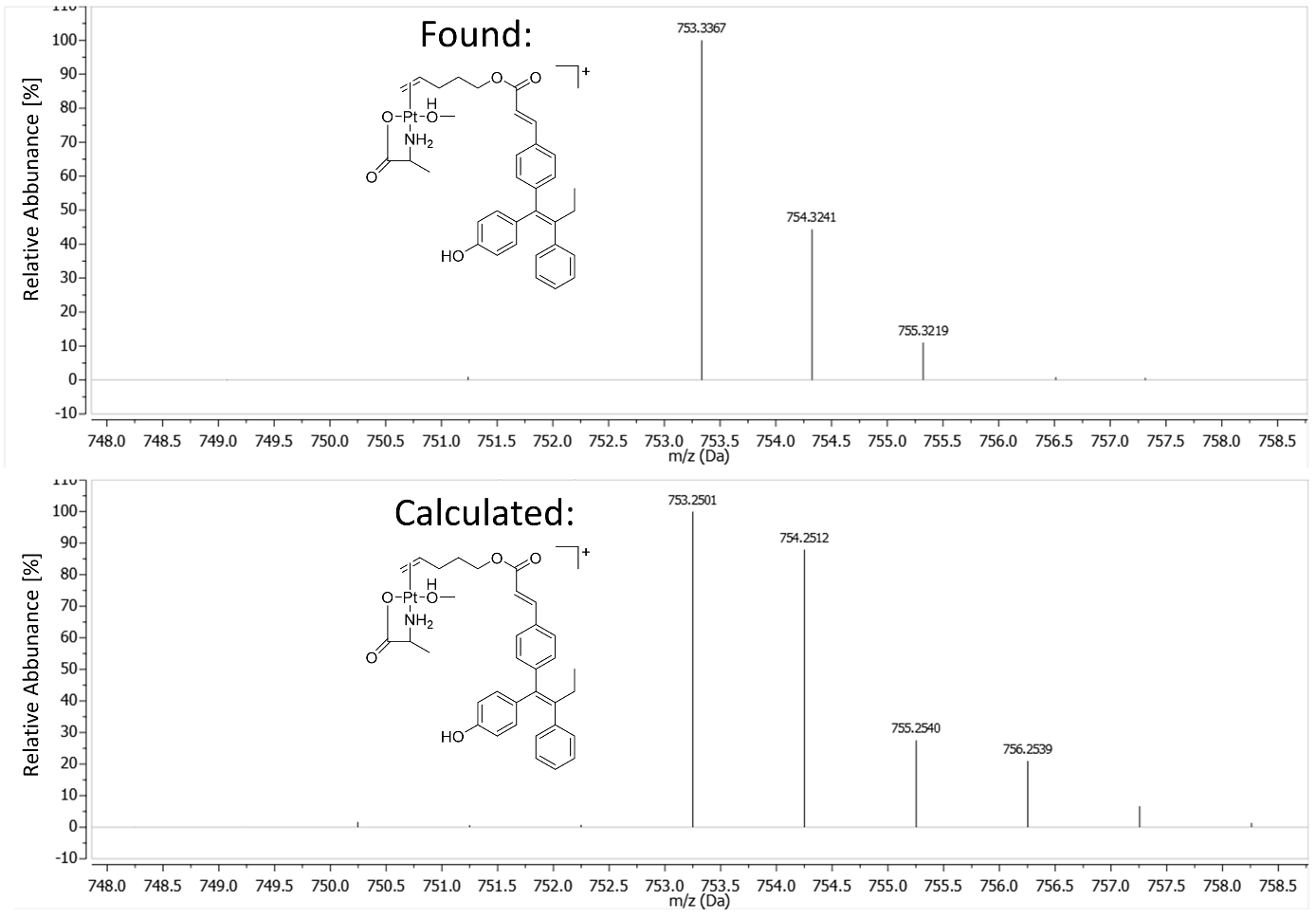
^Calculated and Found Isotopic Distribution Pattern for [GW7604-Pent-Pt(Ala)(CH_3_OH)]^+^

**Figure S28**. Calculated and found isotopic distribution pattern for **[GW7604-Pent-Pt(Ala)(CH_3_OH)]^+^**. Calculations were carried out with MestreNova v14.

## Additional ESI-HR-MS Data from Reactivity Studies Towards 5´-Guanosin Monophosphate


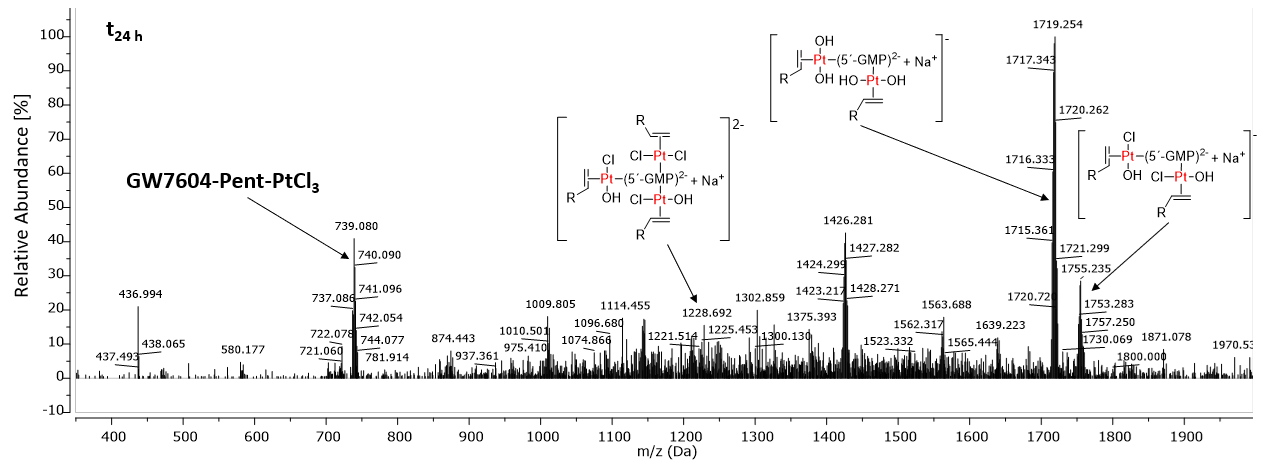


**Figure S29.** ESI-HR-MS spectrum in negative mode obtained from a mixture of **GW7604-Pent-PtCl_3_** in MeOH and 5´-GMP in water (80/20, (v/v)) after 24 h incubation at rt; R-C=C: **GW7604-Pent**.

## Additional Biological Data for TSA-201 and MCF-7 Cells

**Figure S30.** Investigation of metabolic activity in non-cancerous HEK (TSA-201) cells. Cells were incubated for 72 h. The reduction of metabolic activity was measured via an MTT assay as the mean of 3 independent experiments ± SD.

**Figure S31.** Investigation of metabolic activity in MCF-7 cells. Cells were incubated for 72 h. The reduction of metabolic activity was measured via an MTT assay as the mean of 3 independent experiments ± SD.
