# Supplementary material for: Correction to “Development of Cytotoxic GW7604-Zeise’s Salt Conjugates as Multitarget Compounds with Selectivity for Estrogen Receptor-Positive Tumor Cells”
Source: J Med Chem. 2024 Apr 23;67(9):7663. doi: 10.1021/acs.jmedchem.4c00799 (PMC11089497; doi:10.1021/acs.jmedchem.4c00799)
Supplement: Supplementary file 1 — jm4c00799_si_001.pdf [file jm4c00799_si_001.pdf]

Supporting Information for:

Development of Cytotoxic GW7604-Zeise´s Salt Conjugates as Multitarget Compounds with Selectivity for Estrogen Receptor-Positive Tumor Cells

Patricia Grabher,† Paul Kapitza,*,† Nikolas Hörmann,† Amelie Scherfler,† Martin Hermann,# Michael Zwerger,§ Hristo P. Varbanov,† Brigitte Kircher,‡,¤ Daniel Baecker,*,ǀ and Ronald Gust*,†

† Department of Pharmaceutical Chemistry, Institute of Pharmacy, Center for Molecular Biosciences Innsbruck, University of Innsbruck, Innrain 80/82, A-6020 Innsbruck.

# Department of Anesthesiology & Critical Care Medicine, Medical University Innsbruck, Anichstrasse 35, A-6020 Innsbruck.

§ Department of Pharmacognosy, Institute of Pharmacy, Center for Molecular Biosciences Innsbruck, University of Innsbruck, Innrain 80/82, A-6020 Innsbruck.

‡ Department of Internal Medicine V, Haematology & Oncology, Immunobiology and Stem Cell Laboratory, Medical University Innsbruck, Anichstrasse 35, A-6020 Innsbruck.

¤ Tyrolean Cancer Research Institute, Innrain 66, A-6020 Innsbruck.

ǀ Department of Pharmaceutical and Medicinal Chemistry, Institute of Pharmacy, Freie Universität Berlin, Königin-Luise-Straße 2+4, D-14195 Berlin.

Corresponding Authors:

Paul Kapitza (paul.kapitza@uibk.ac.at)

Daniel Baecker (d.baecker@fu-berlin.de)

Ronald Gust (gust.ronald@gmail.com)

TABLE OF CONTENTS

1. ^{1}H, ^{13}C, $^{1}H/^{1}H$ COSY, and $^{1}H/^{13}C$ HSQC NMR Spectra of the GW7604-Alk-$PtCl_3$ Complexes S2
2. Conformational description of GW7604-But-$PtCl_3$ Isomers S10
3. NMR Spectra from Stability Studies in Organic Solvents S11
4. HPLC Chromatograms of the GW7604-Alk-$PtCl_3$ Complexes S14
5. MS spectrum of the methanolic extract from DMEM incubated with GW7604-Pent-$PtCl_3$ and the calculated isotopic distribution pattern for [GW7604-Pent-Pt(Ala)(CH_3OH)]$^+$ S15
6. Additional ESI-HR-MS Data from Reactivity Studies Towards 5´-Guanosin Monophosphate S16
7. Additional Biological Data for TSA-201 and MCF-7 Cells S16

1. ^{1}H, ^{13}C, $^{1}H/^{1}H$ COSY, AND $^{1}H/^{13}C$ HSQC NMR SPECTRA OF THE GW7604-ALK-PTCL$_3$ COMPLEXES

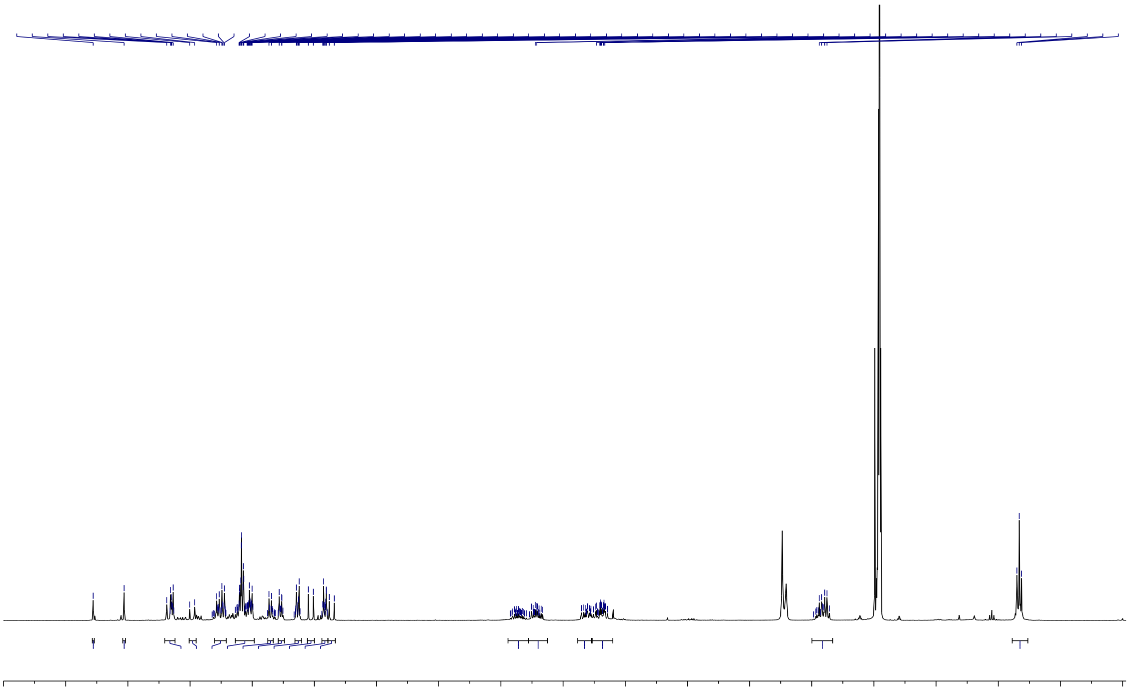

Figure S1. ^{1}H NMR (400 MHz) of **GW7604-Prop-PtCl$_3$** in acetone-*d$_6$*

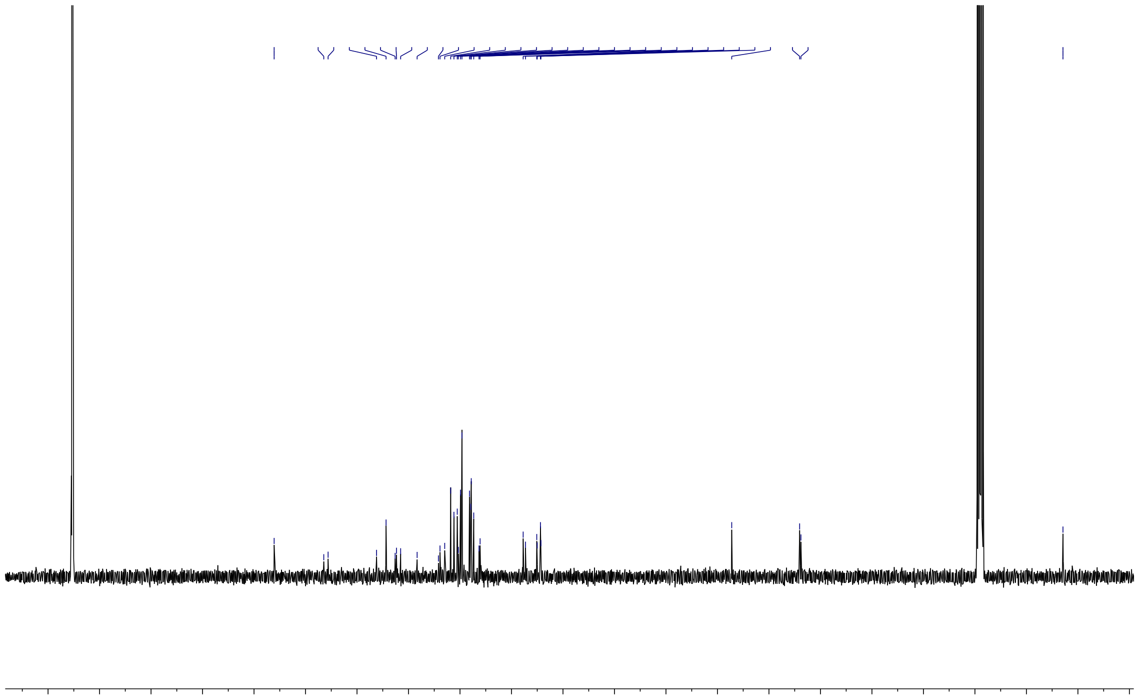

Figure S2. ^{13}C NMR (101 MHz) of **GW7604-Prop-PtCl$_3$** in acetone-*d$_6$*.

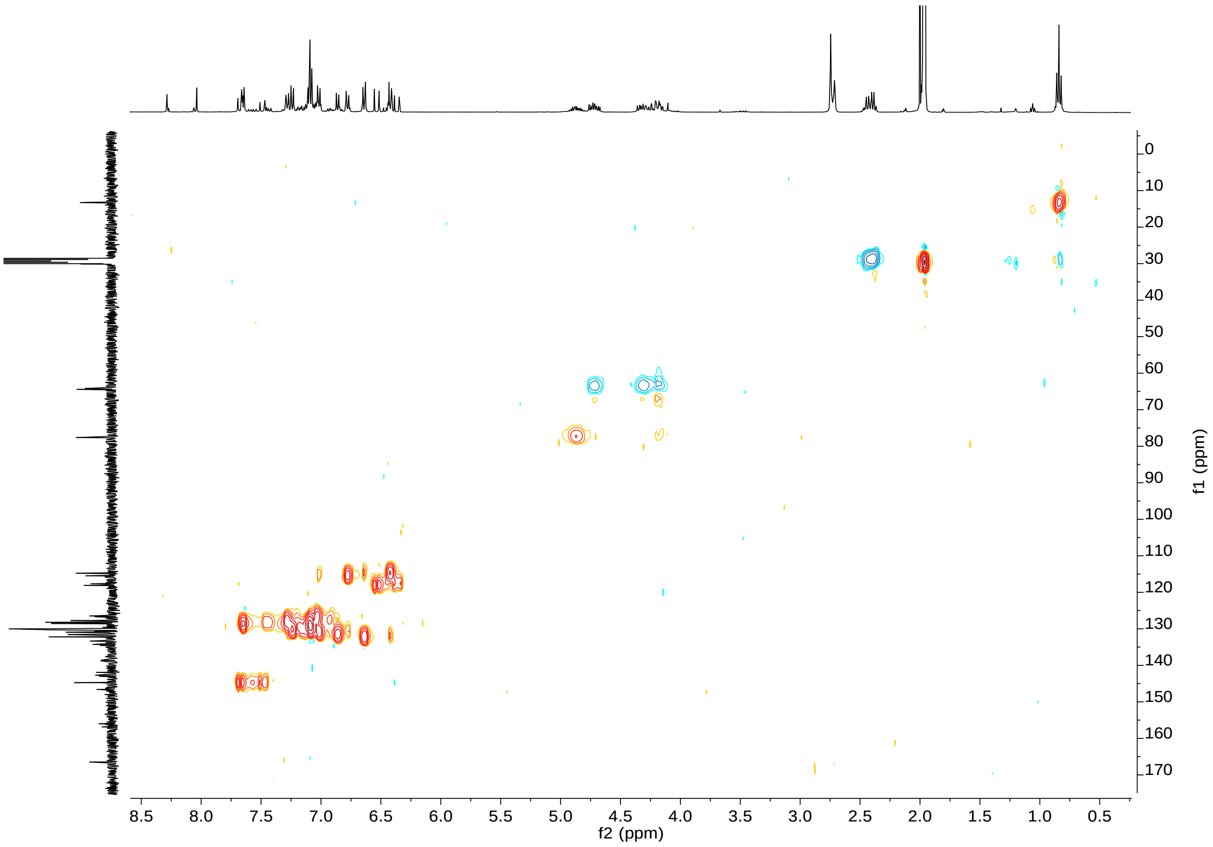

Figure S3. $^{1}H/^{13}C$ HSQC of **GW7604-Prop-PtCl₃** in acetone-d_6

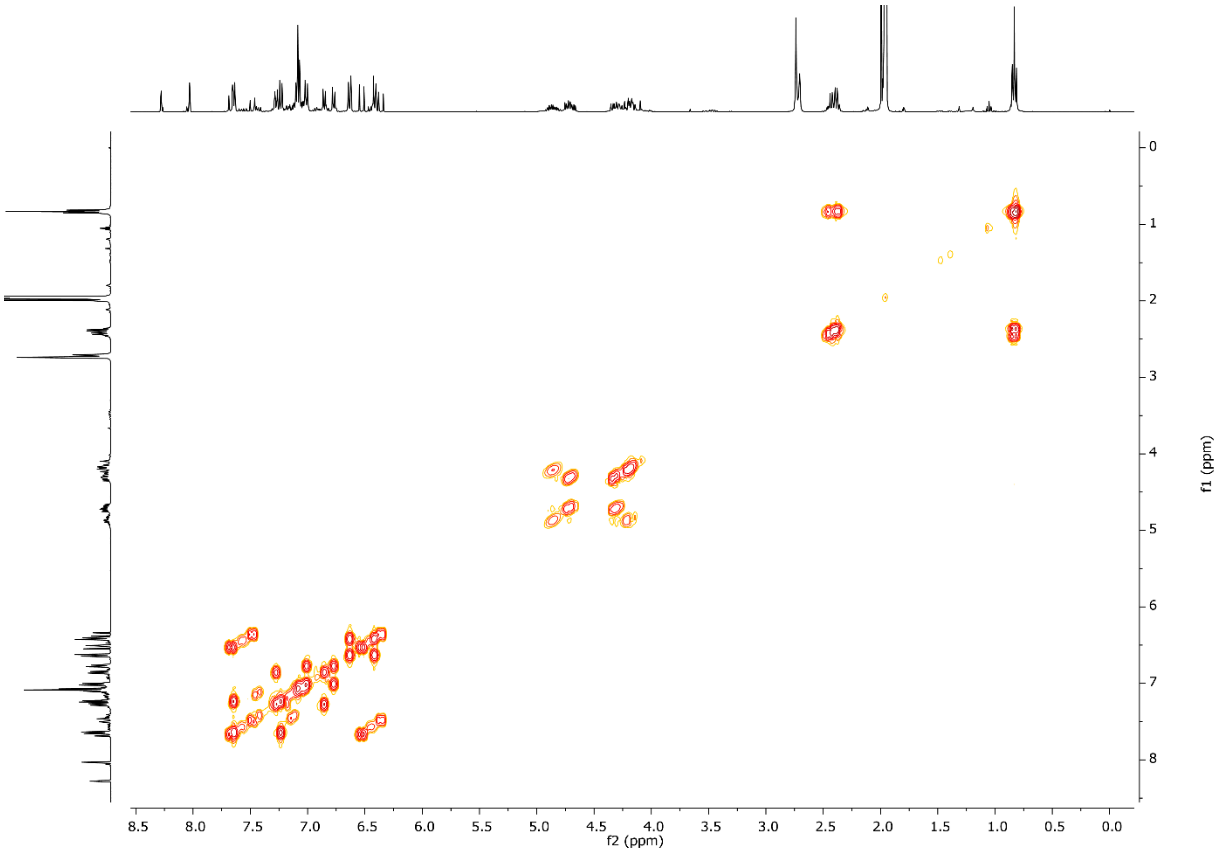

Figure S4. ^{1}H COSY of **GW7604-Prop-PtCl₃** in acetone-d_6.

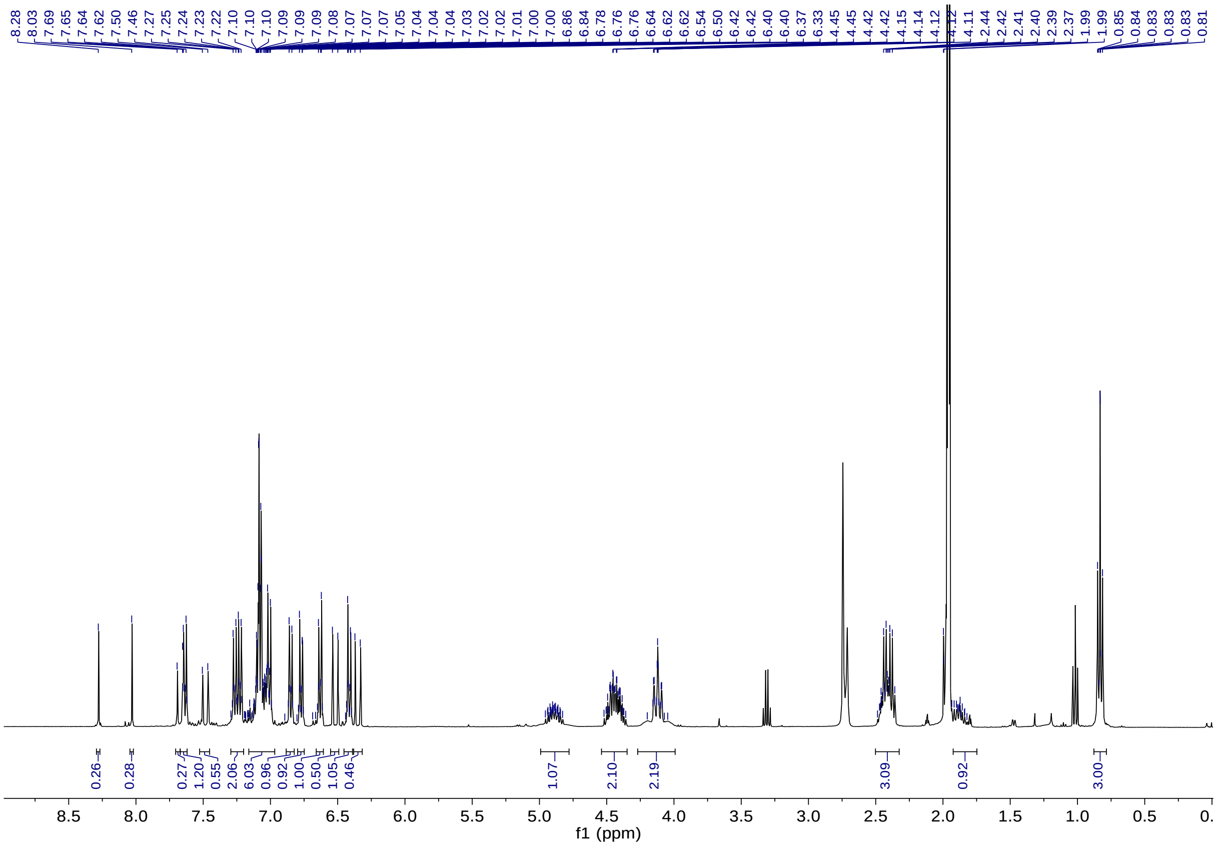

Figure S5. ^{1}H NMR (400 MHz) of the **GW7604-But-PtCl$_3$** in acetone-*d$_6$*.

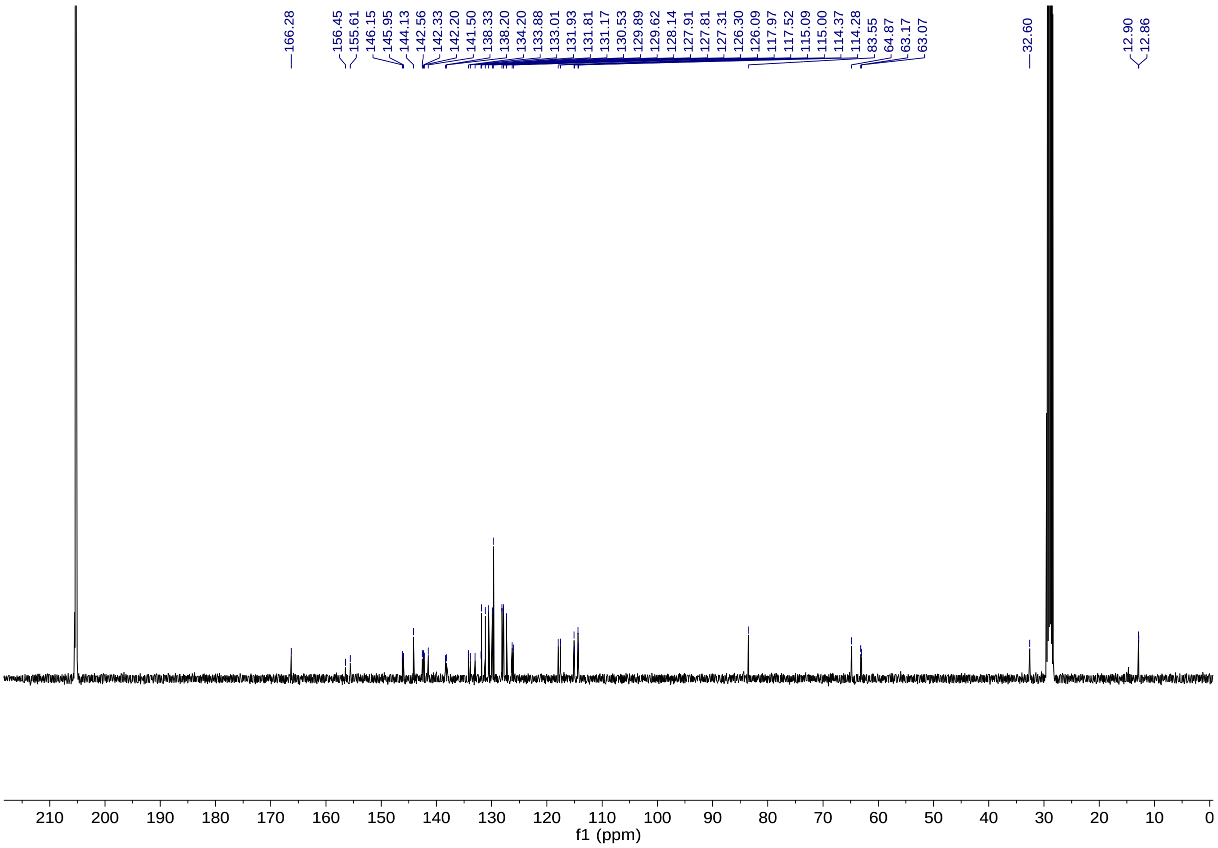

Figure S6. ^{13}C NMR (101 MHz) of **GW7604-But-PtCl$_3$** in acetone-*d$_6$*.

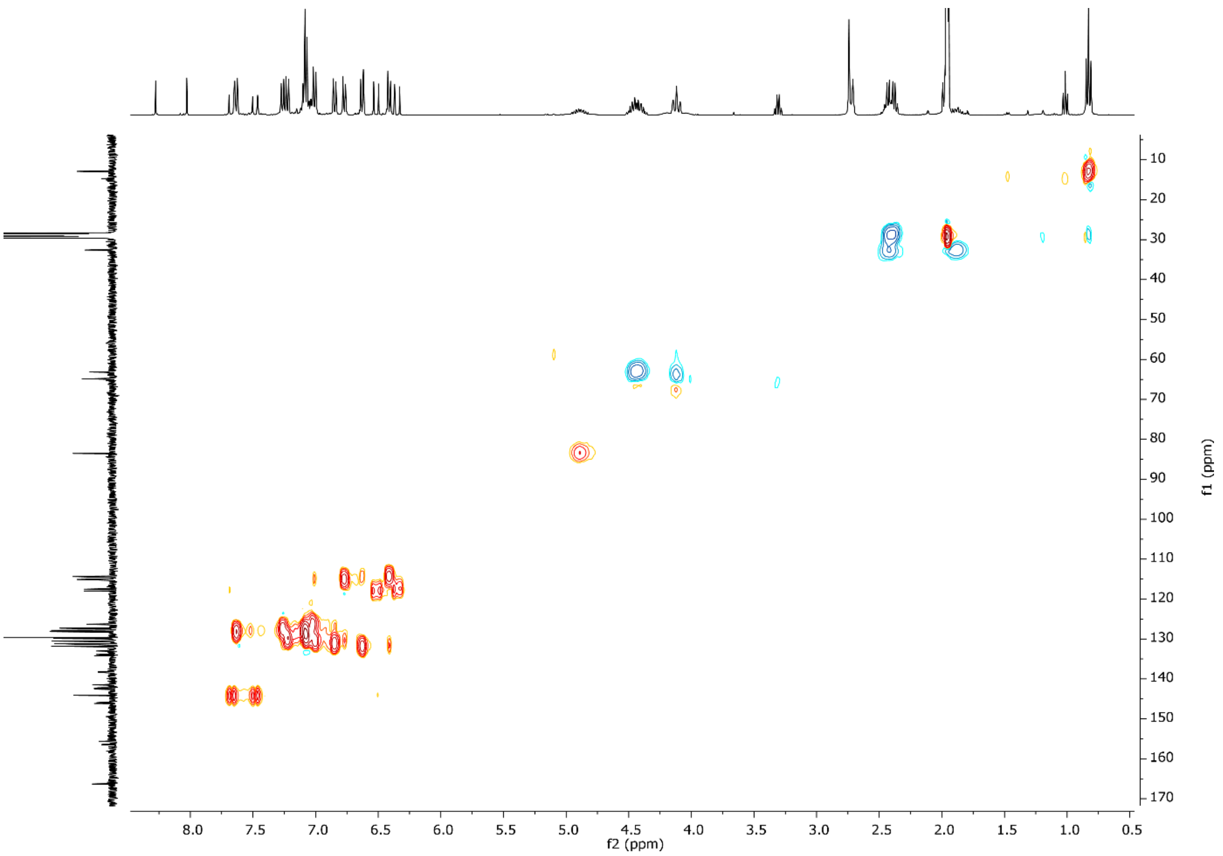

Figure S7. $^{1}H/^{13}C$ HSQC of **GW7604-But-PtCl₃** in acetone-d_6.

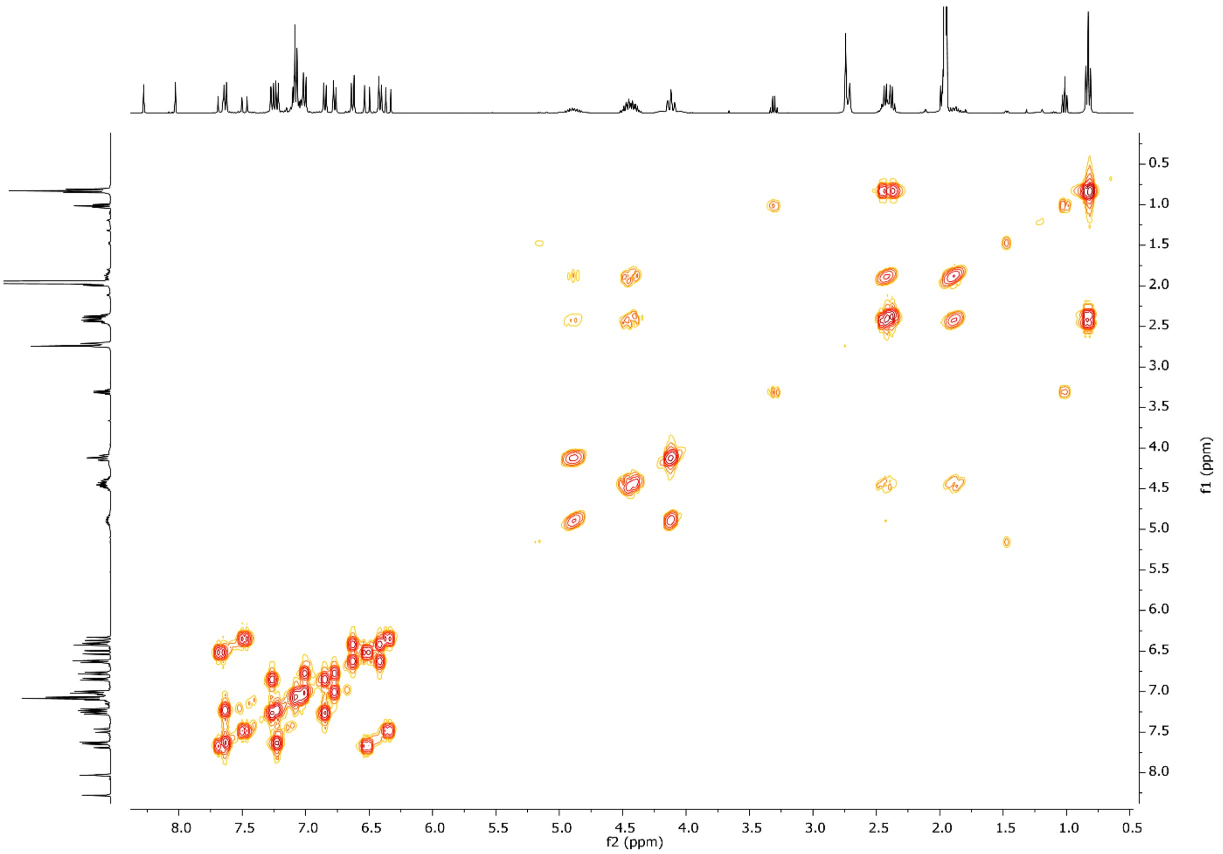

Figure S8. ^{1}H COSY of **GW7604-But-PtCl₃** in acetone-d_6.

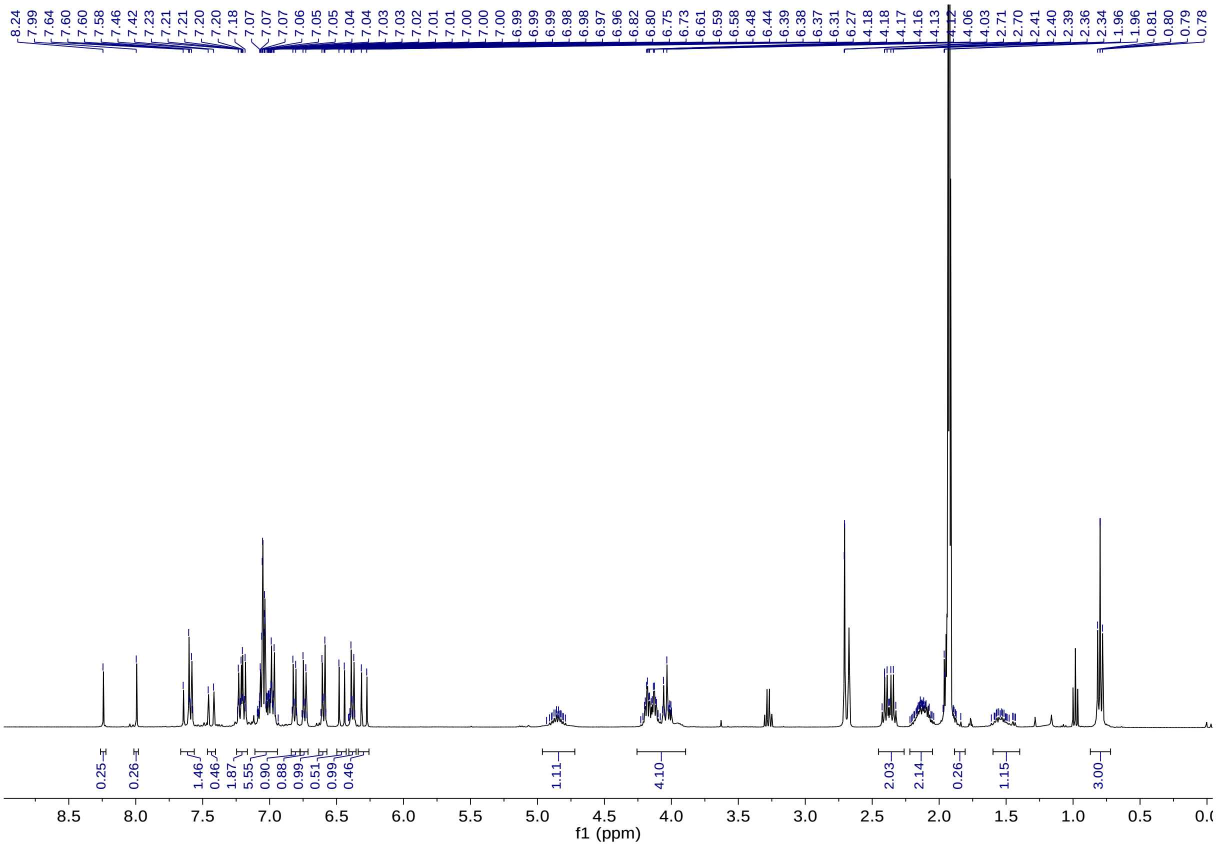

Figure S9. ^{1}H NMR (400 MHz) of **GW7604-Pent-PtCl$_3$** in acetone-*d$_6$*.

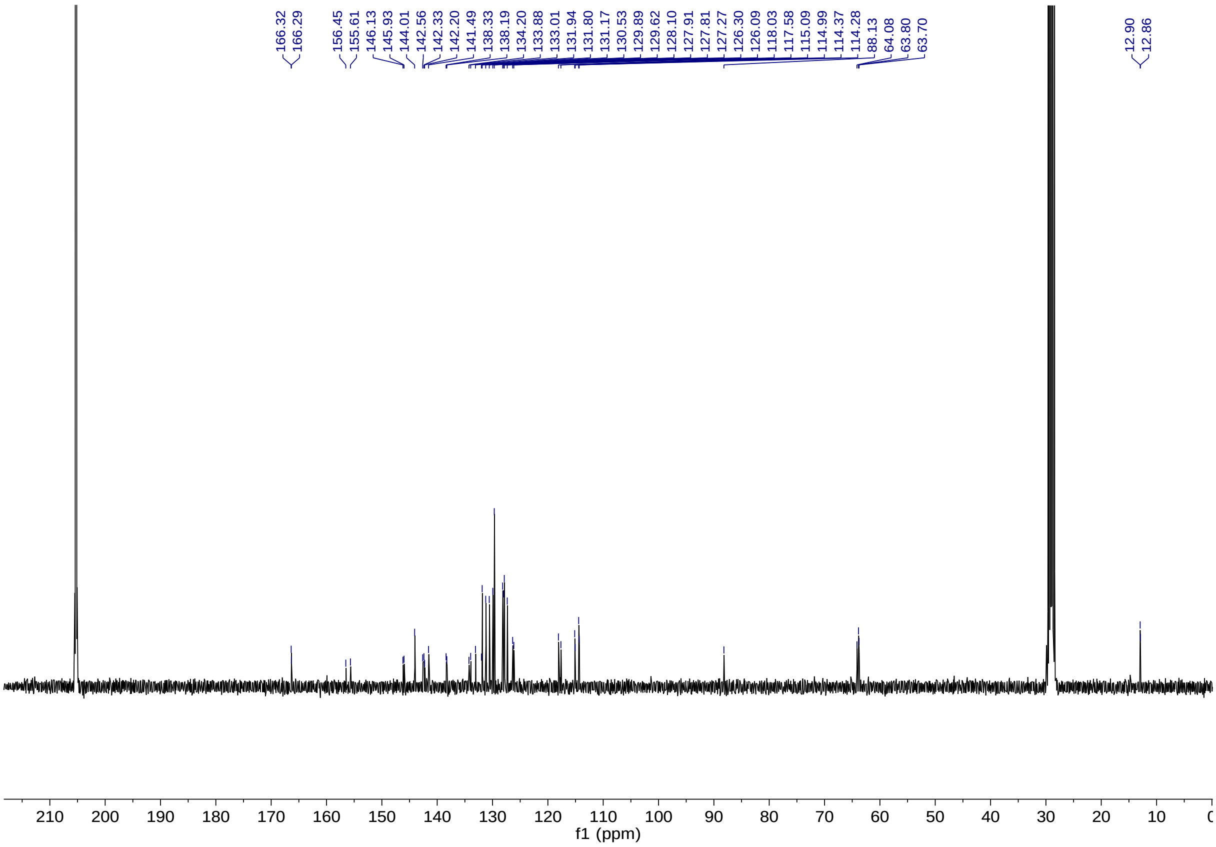

Figure S10. ^{13}C NMR (101 MHz) of **GW7604-Pent-PtCl$_3$** in acetone-*d$_6$*.

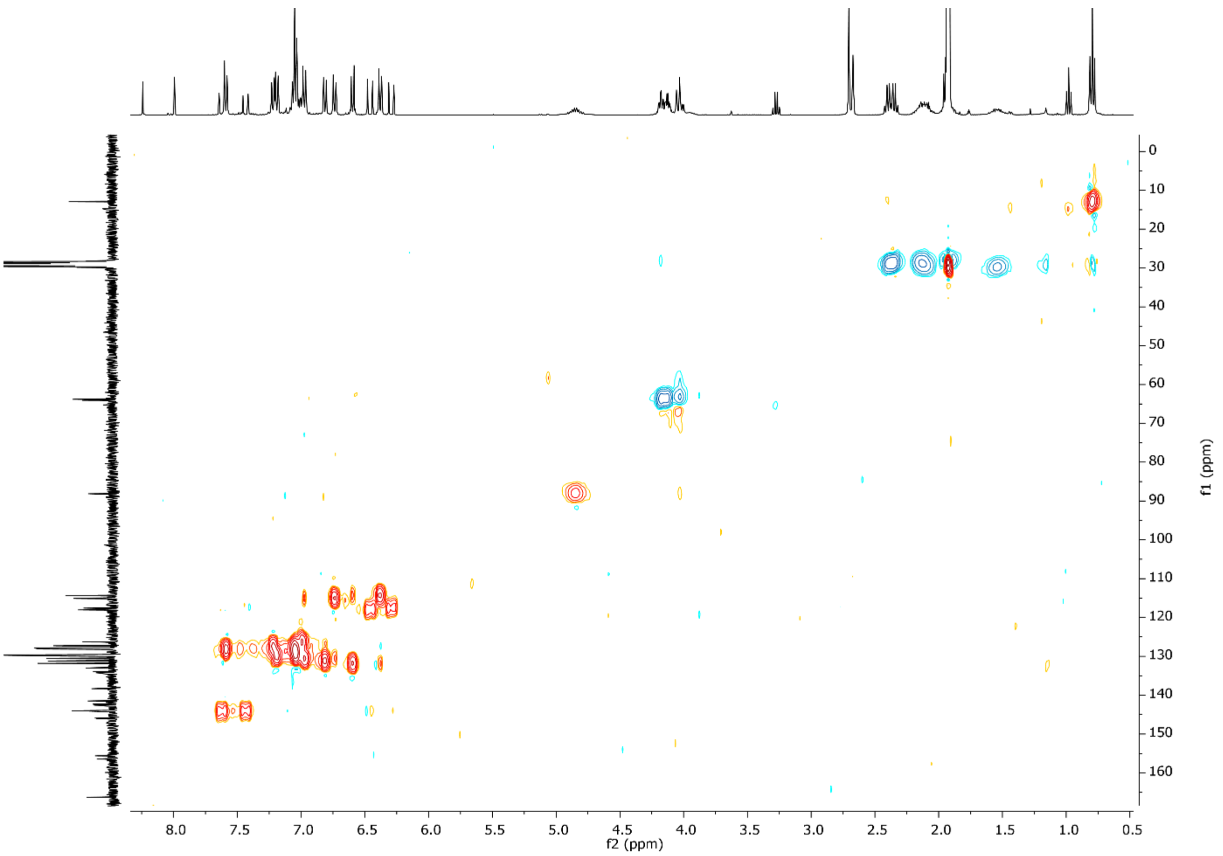

Figure S11. $^{1}H/^{13}C$ HSQC of the **GW7604-Pent-PtCl₃** in acetone-*d₆.*

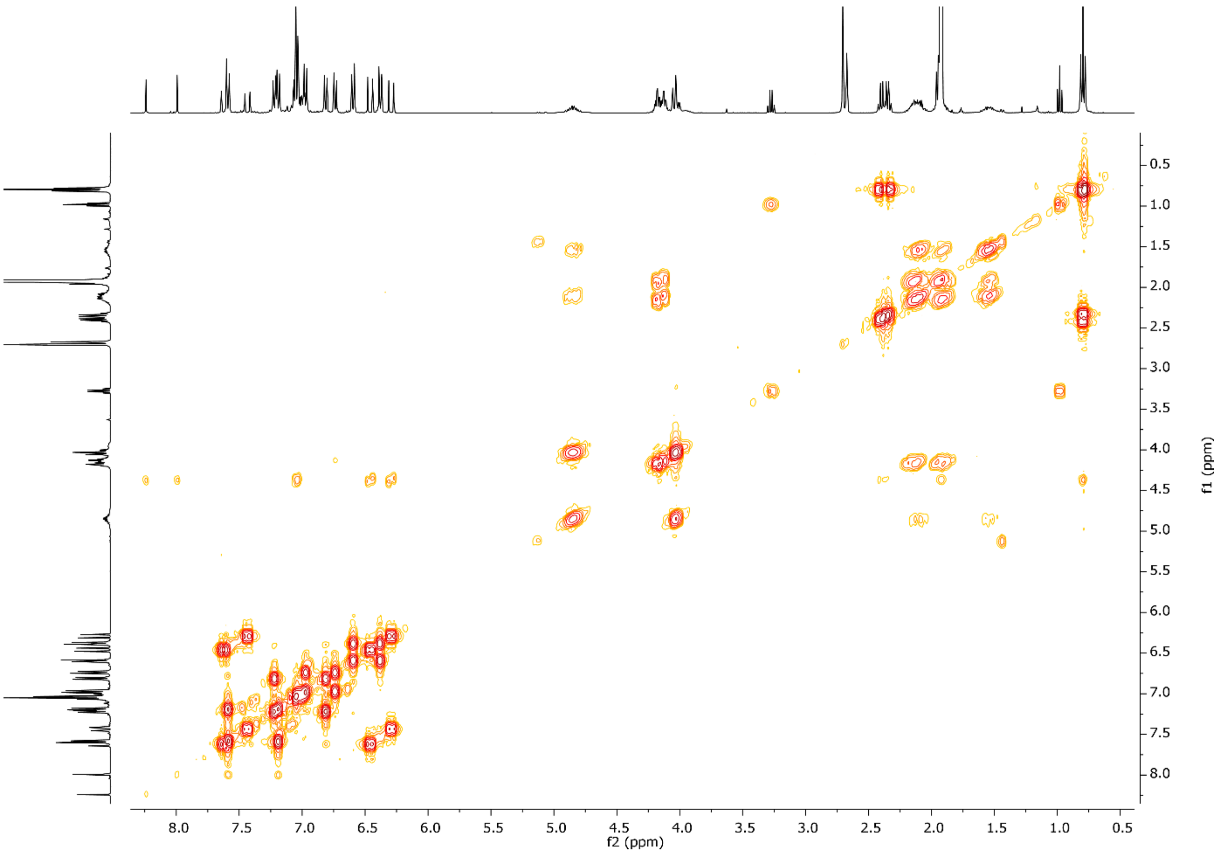

Figure S12. ^{1}H COSY of the **GW7604-Pent-PtCl₃** in acetone-*d₆.*

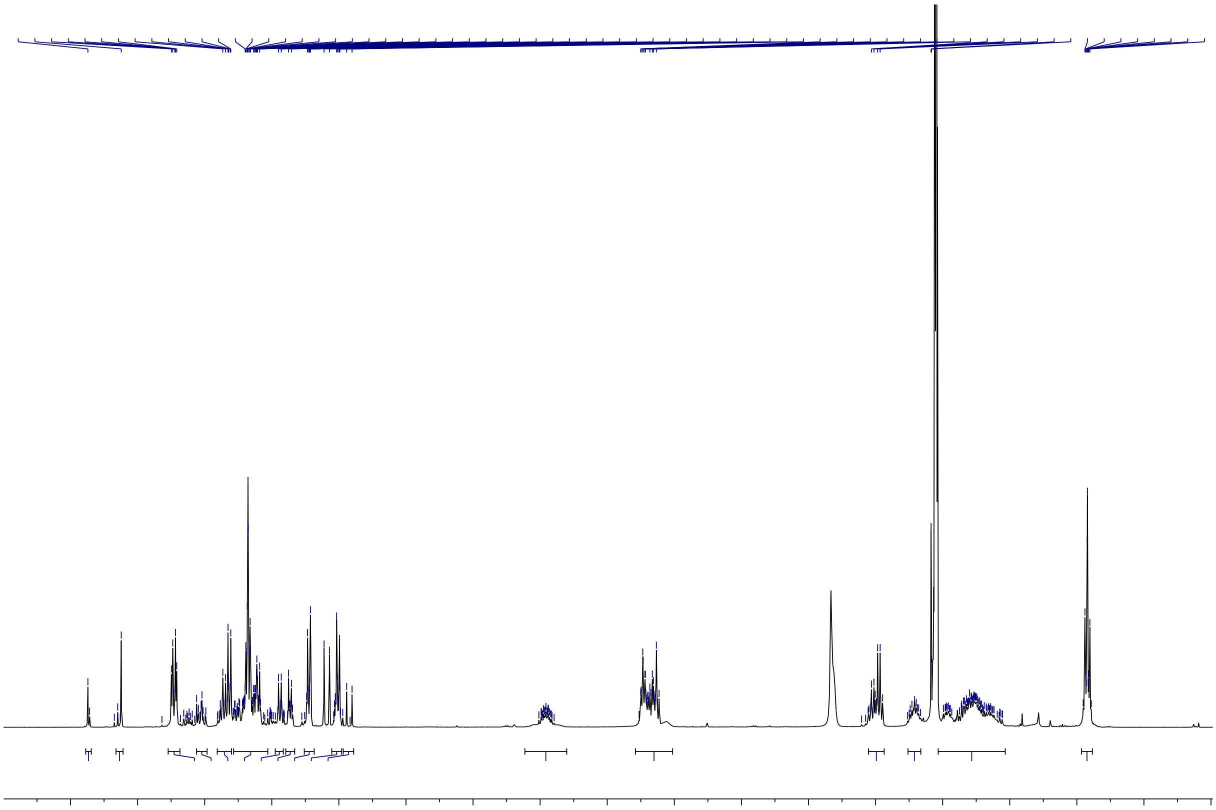

Figure S13. ^{1}H NMR (400 MHz) of **GW7604-Hex-PtCl$_3$** in acetone-d_6.

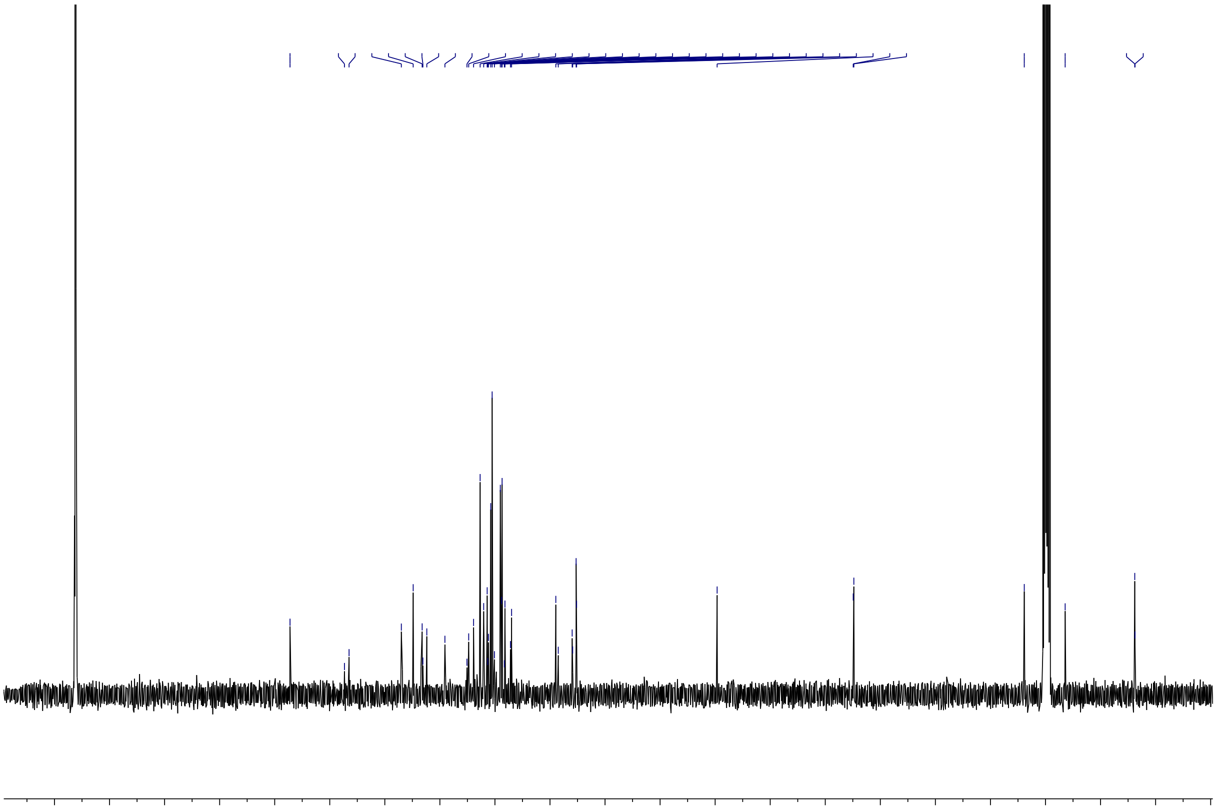

Figure S14. ^{13}C NMR (101 MHz) of **GW7604-Hex-PtCl$_3$** in acetone-d_6.

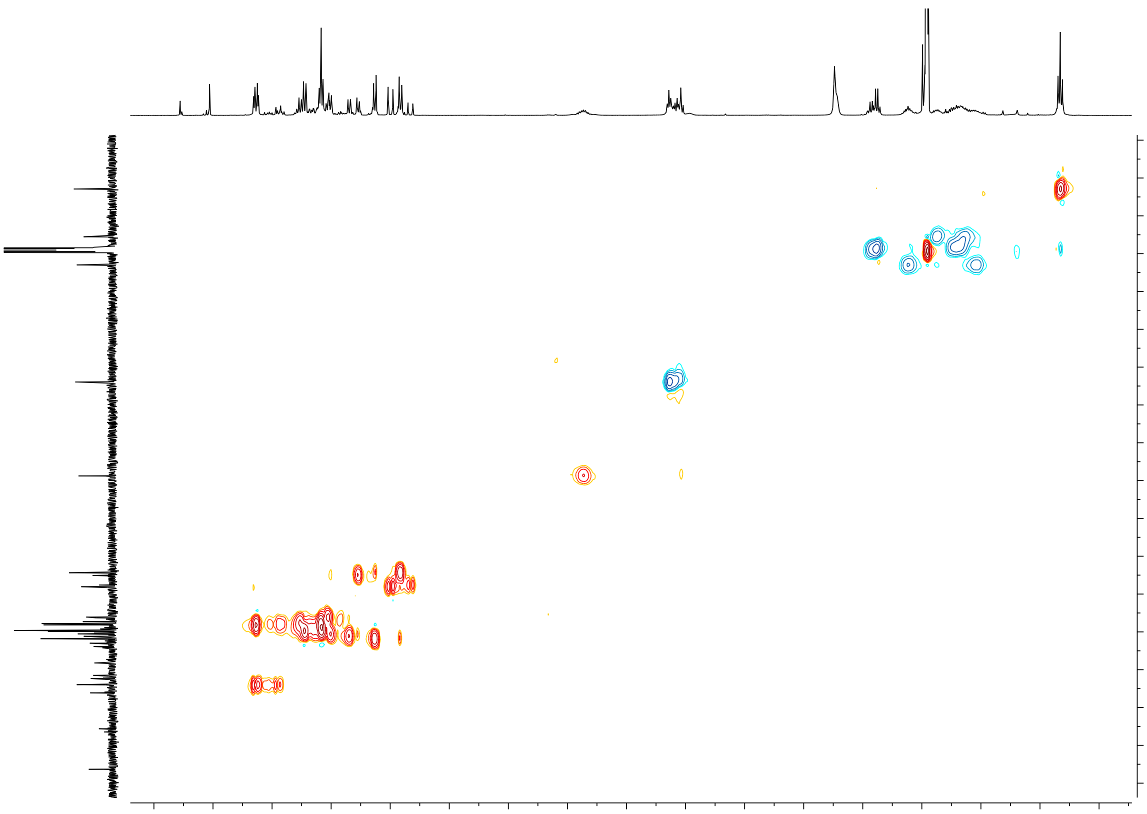

Figure S15. $^1H/^{13}C$ HSQC of **GW7604-Hex-PtCl₃** in acetone-*d*₆.

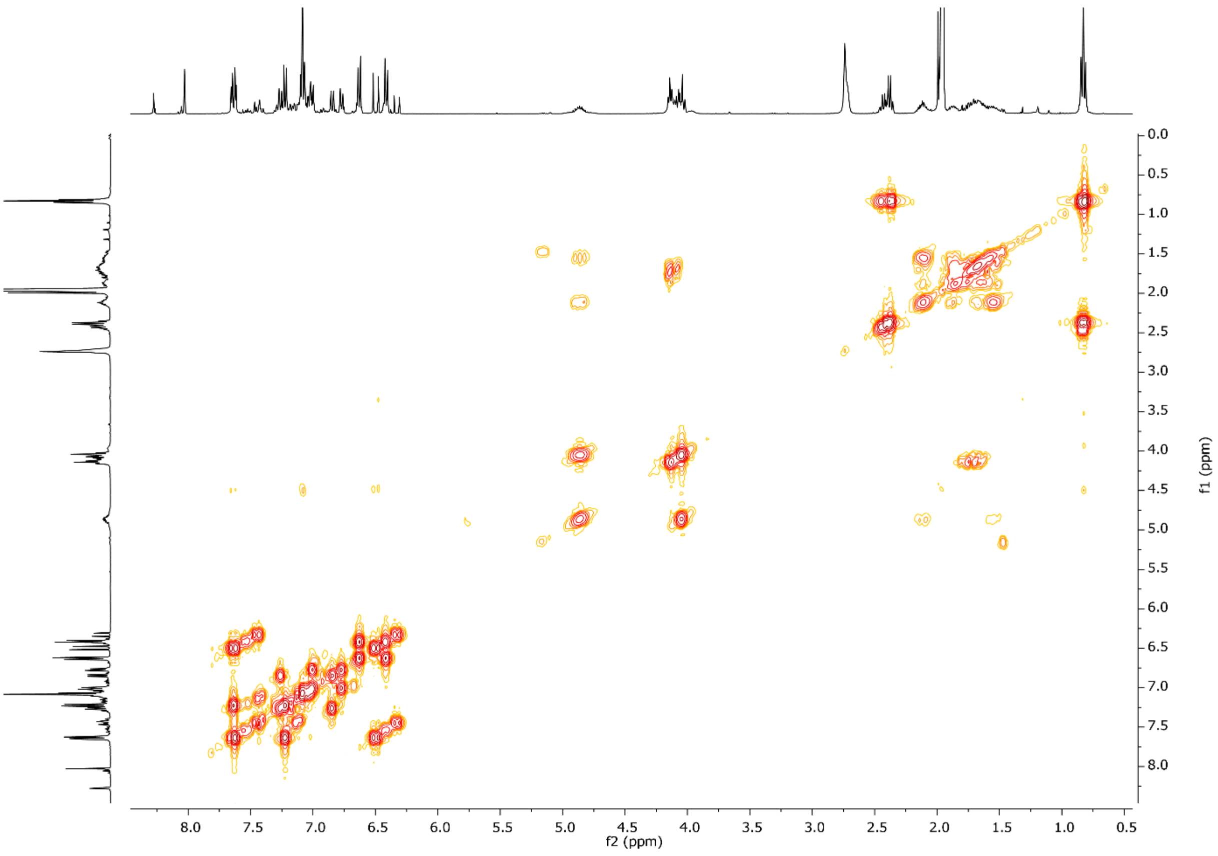

Figure S16. ^1H COSY of **GW7604-Hex-PtCl₃** in acetone-*d*₆.

2. CONFORMATIONAL DESCRIPTION OF GW7604-BUT-$PtCl_3$ ISOMERS

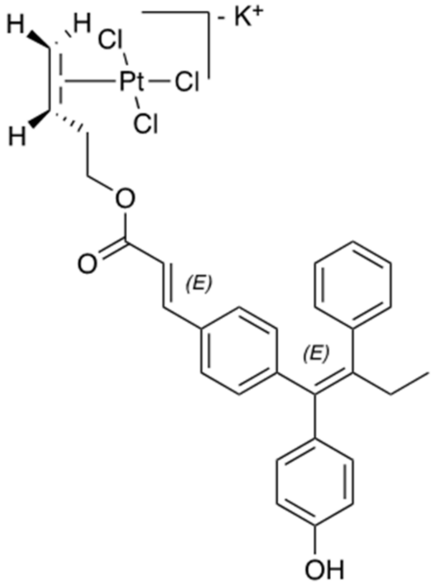

Isomer I (re,E,E)

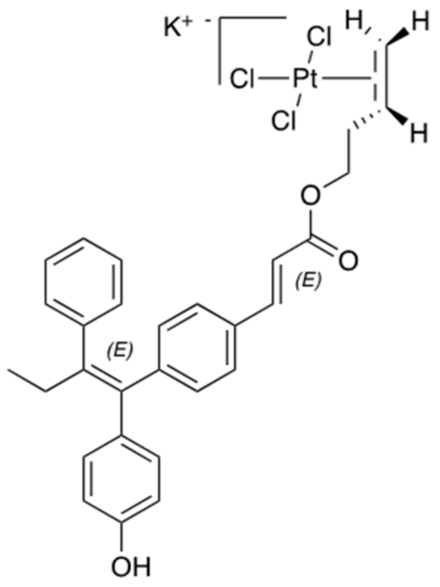

Isomer II (si,E,E)

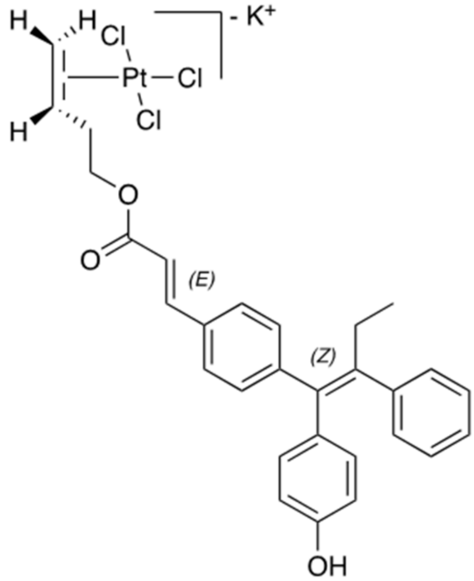

Isomer III (re,E,Z)

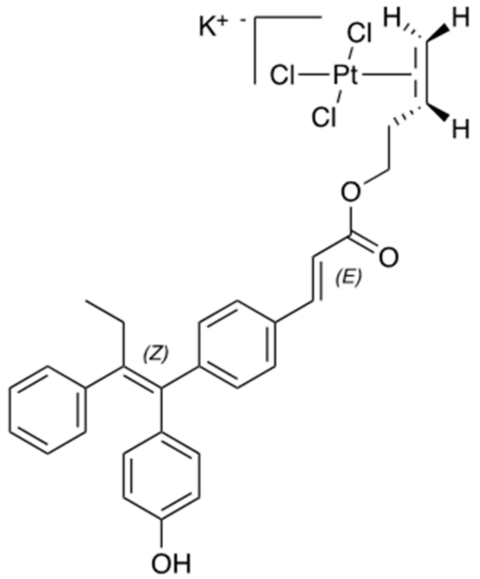

Isomer IV (si,E,Z)

Figure S17. Conformational description of **GW7604-But-$PtCl_3$** isomers I and II as well as isomers III and IV are enantiomers. The enantiomeric pairs I/II and III/IV are diastereomeric to each other. They cause for instance two sets of Signals in the ^1H NMR spectrum (Figure 1).

3. NMR Spectra from Stability Studies in Organic Solvents

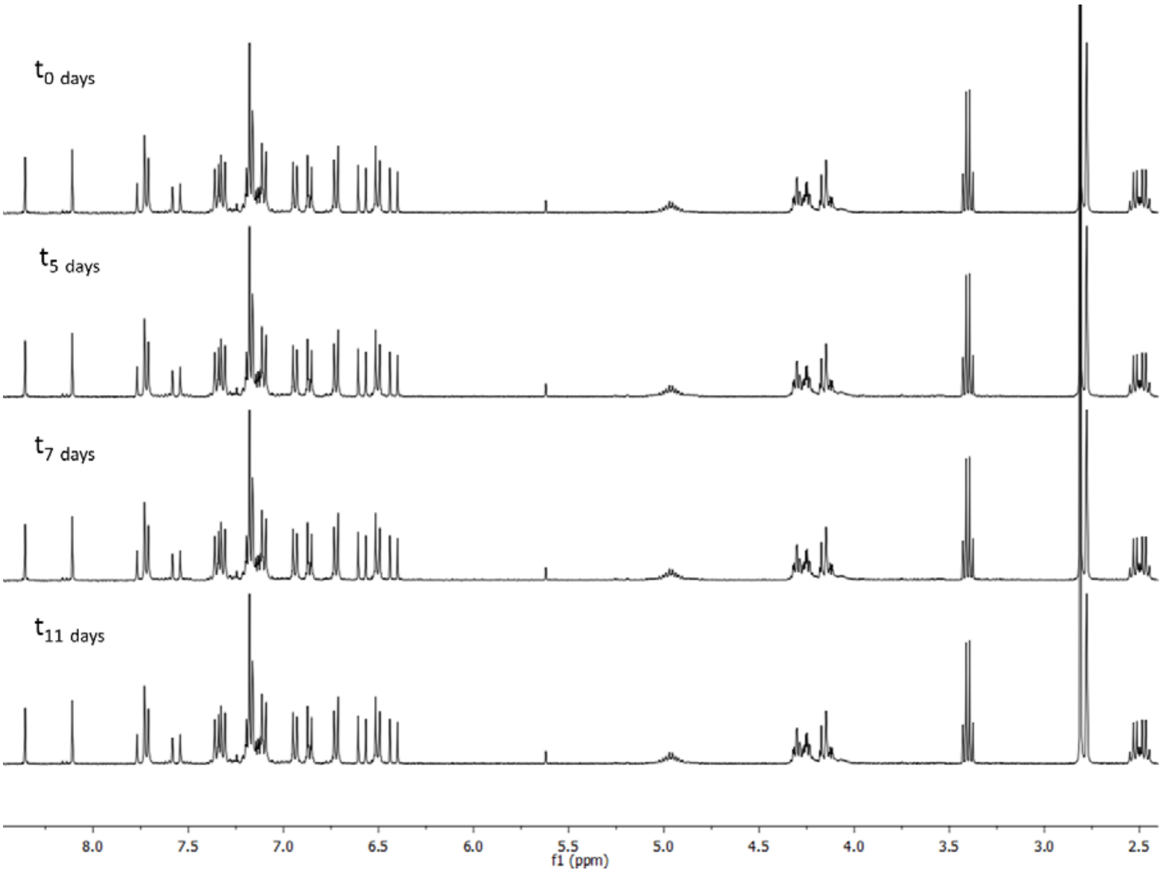

Figure S18. ^{1}H NMR (400 MHz) of **GW7604-Pent-PtCl₃** in acetone-d_6.

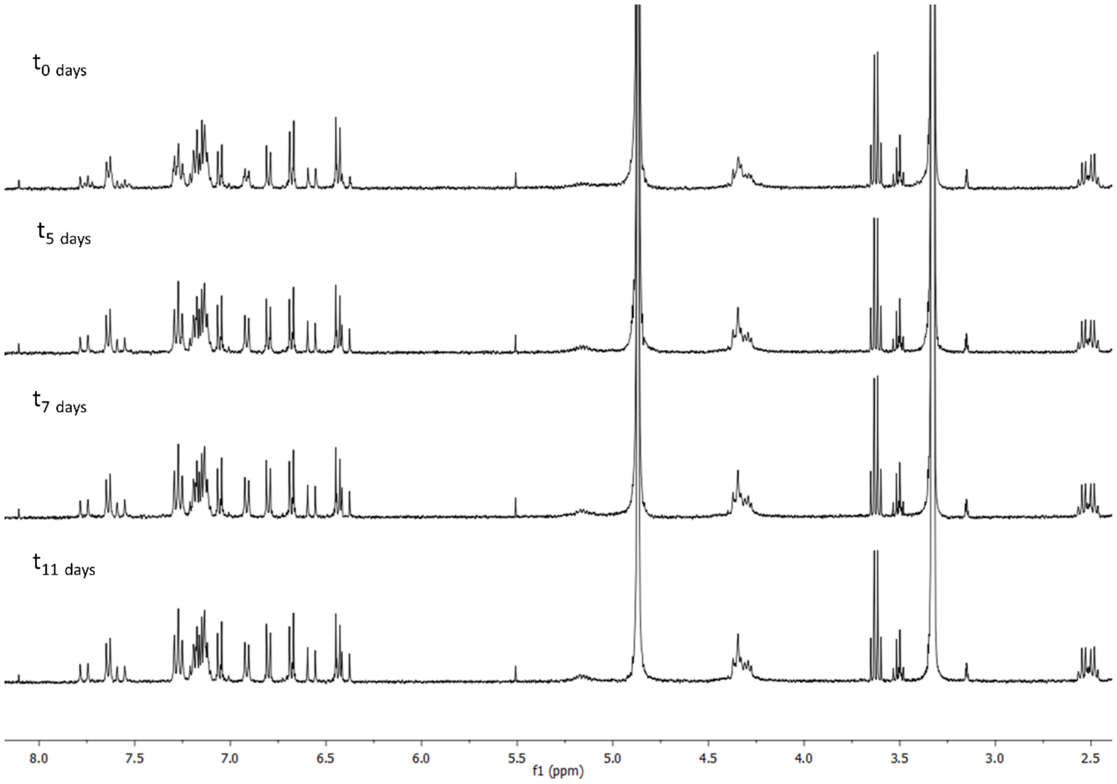

Figure S19. ^{1}H NMR (400 MHz) of **GW7604-Pent-PtCl₃** in methanol-d_4.

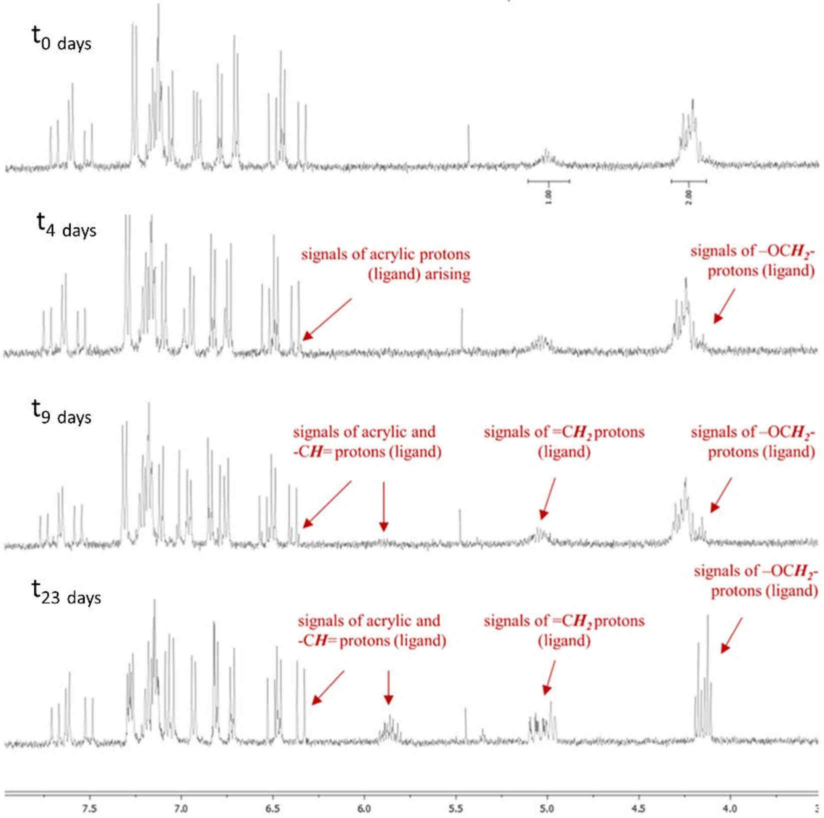

Figure S20. ^{1}H NMR (400 MHz) of **GW7604-Pent-PtCl$_3$** in acetonitrile-*d*$_3$.

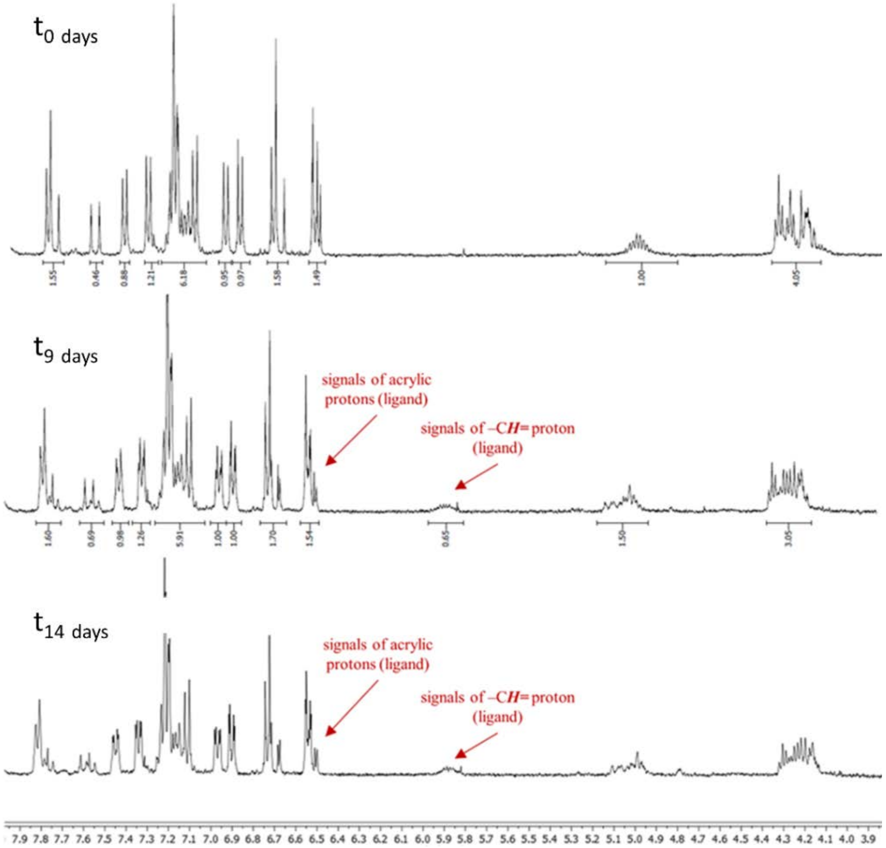

Figure S21. ^{1}H NMR (400 MHz) of **GW7604-Pent-PtCl$_3$** in DMF-*d*$_7$.

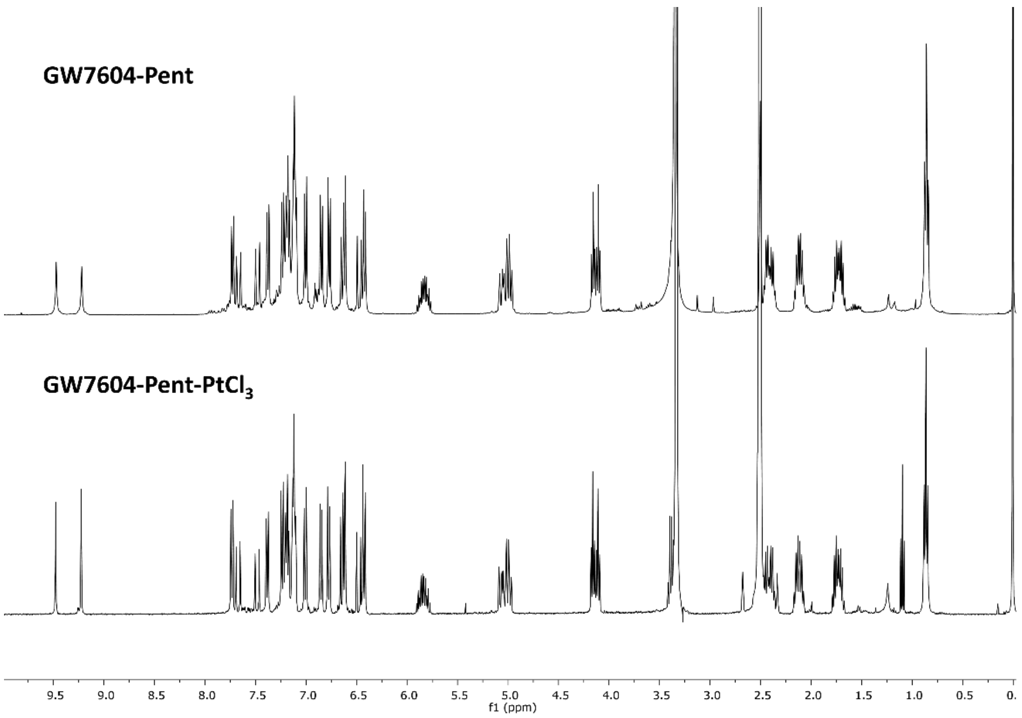

Figure S22. ^{1}H NMR spectra (400 MHz) of **GW7604-Pent** and **GW7604-Pent-PtCl₃** recorded in DMSO-*d₆* after 5 min of incubation at rt. The spectrum of **GW7604-Pent-PtCl₃** contains traces of diethyl ether.

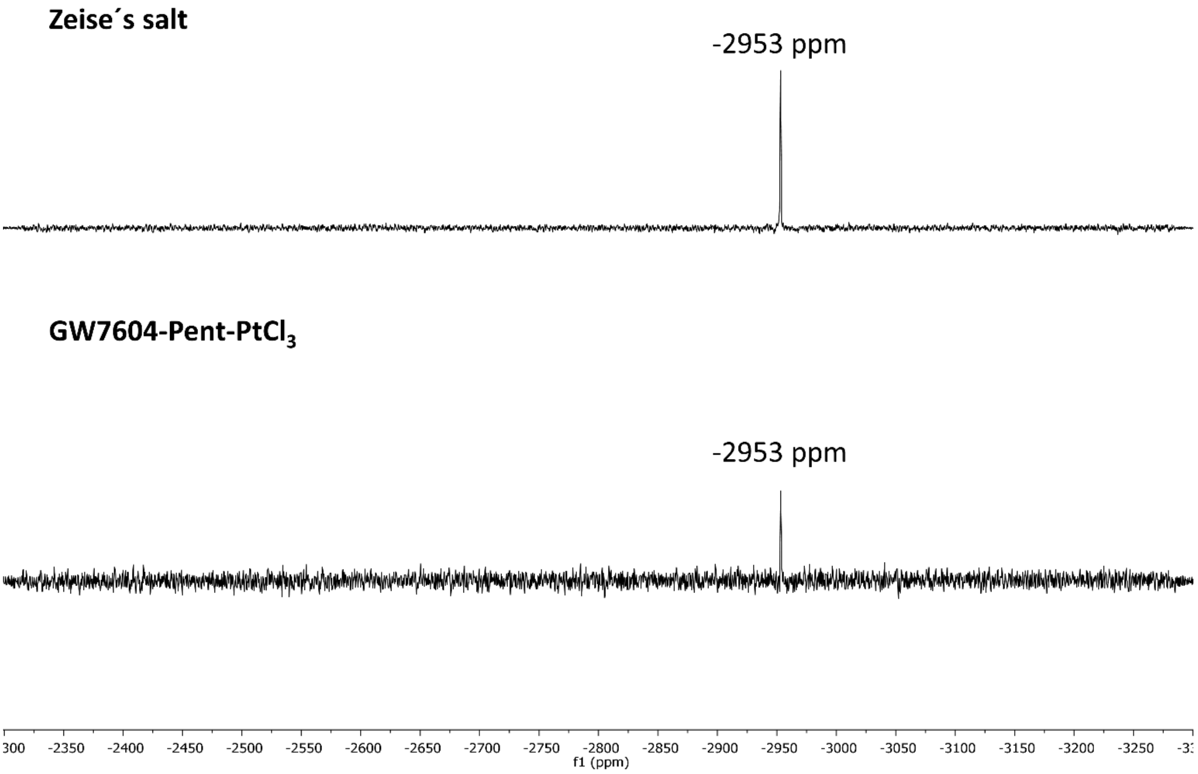

Figure S23. ^{195}Pt NMR spectra (86 MHz) of **Zeise´s salt** and **GW7604-Pent-PtCl₃** recorded in DMSO-*d₆* after 24 h of incubation at rt.

4. HPLC CHROMATOGRAMS OF THE GW7604-ALK-PTCL3 COMPLEXES

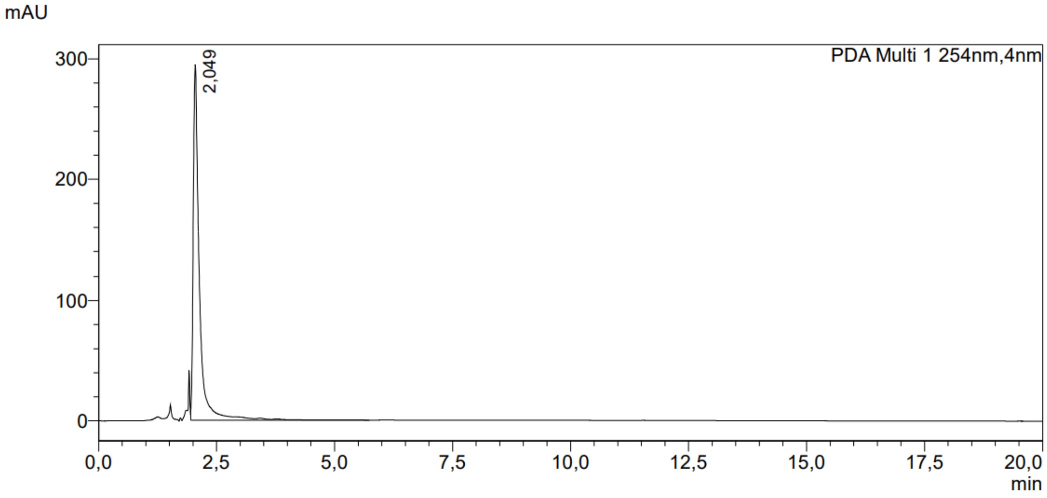

Figure S24. HPLC chromatogram of **GW7604-Prop-PtCl₃**.

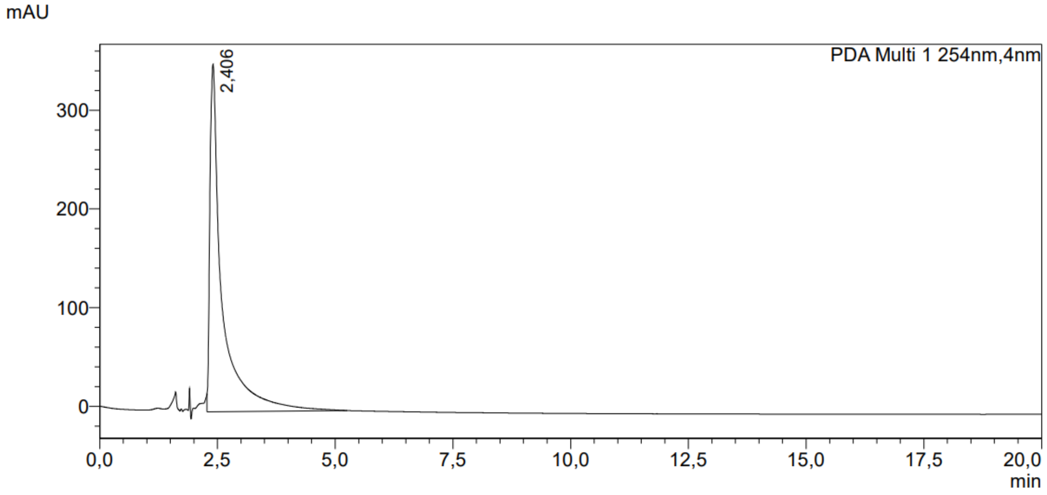

Figure S25. HPLC chromatogram of **GW7604-But-PtCl₃**.

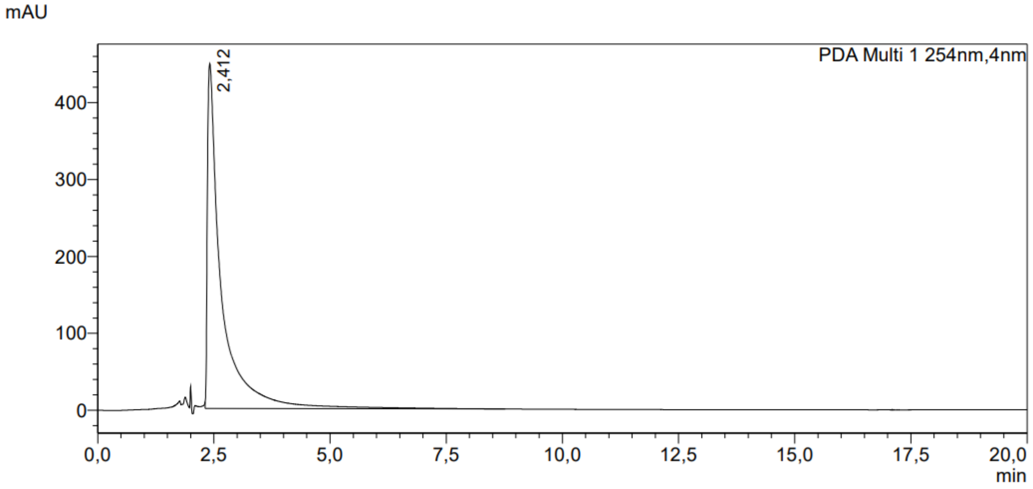

Figure S26. HPLC chromatogram of **GW7604-Pent-PtCl₃**.

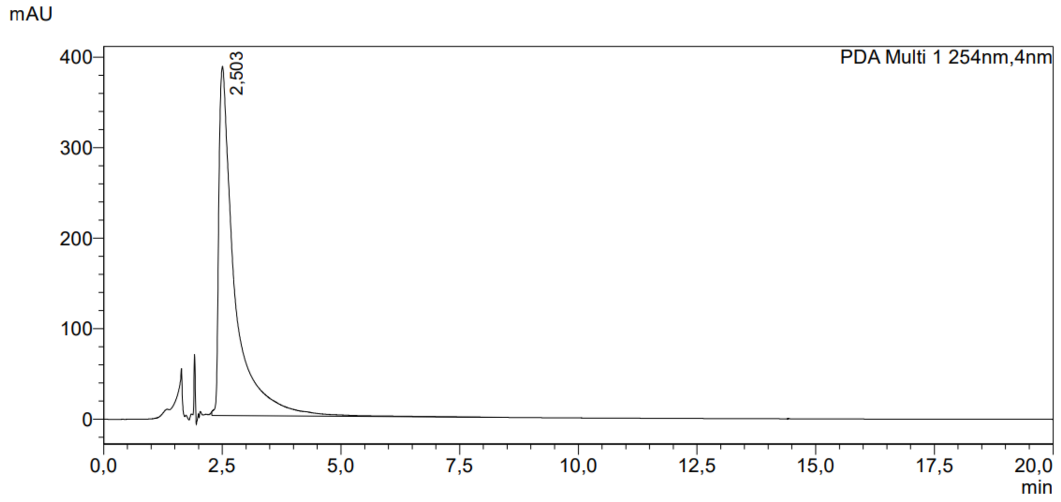

Figure S27. HPLC chromatogram of **GW7604-Hex-PtCl$_3$**.

5. MS spectrum of the methanolic extract from DMEM incubated with GW7604-Pent-PtCl$_3$ and the calculated isotopic distribution pattern for [GW7604-Pent-Pt(Ala)(CH$_3$OH)]$^+$

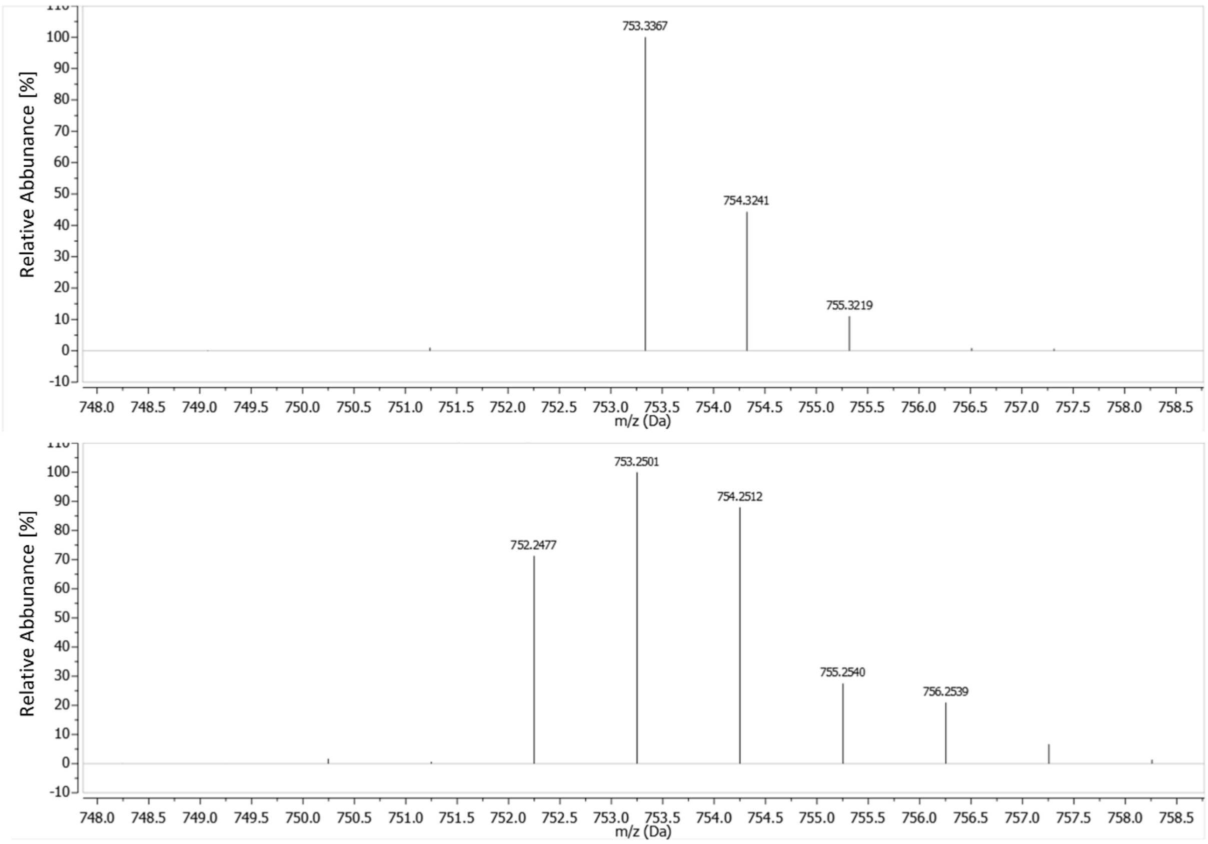

Figure S28. Comparison of the found (upper MS spectrum) with the MS spectrum (lower figure) calculated for the supposed adduct **[GW7604-Pent-Pt(Ala)(CH$_3$OH)]$^+$**. Calculations were carried out with MestreNova v14.

6. Additional ESI-HR-MS Data from Reactivity Studies Towards 5´-Guanosin Monophosphate

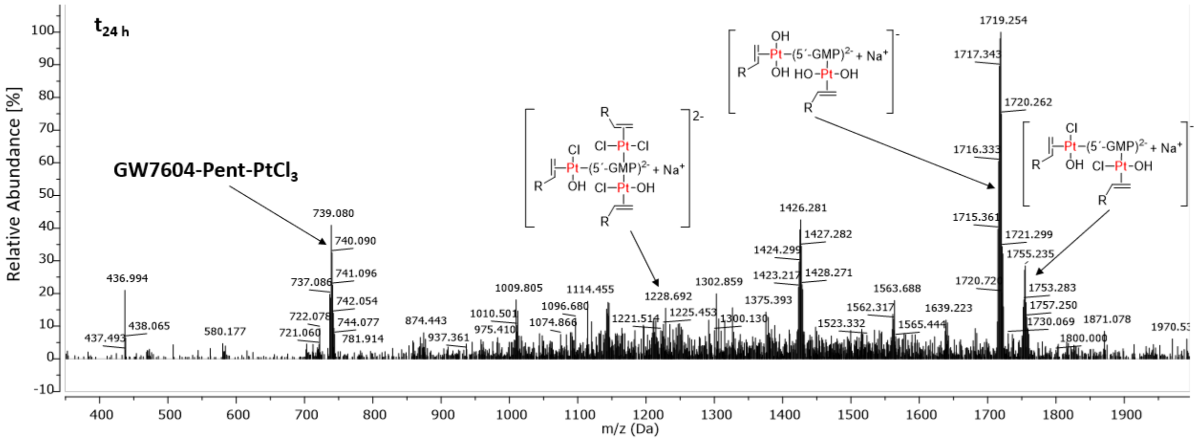

Figure S29. ESI-HR-MS spectrum in negative mode obtained from a mixture of **GW7604-Pent-PtCl₃** in MeOH and 5´-GMP in water (80/20, (*v*/*v*)) after 24 h incubation at rt; R-C=C: **GW7604-Pent**.

7. Additional Biological Data for TSA-201 and MCF-7 Cells

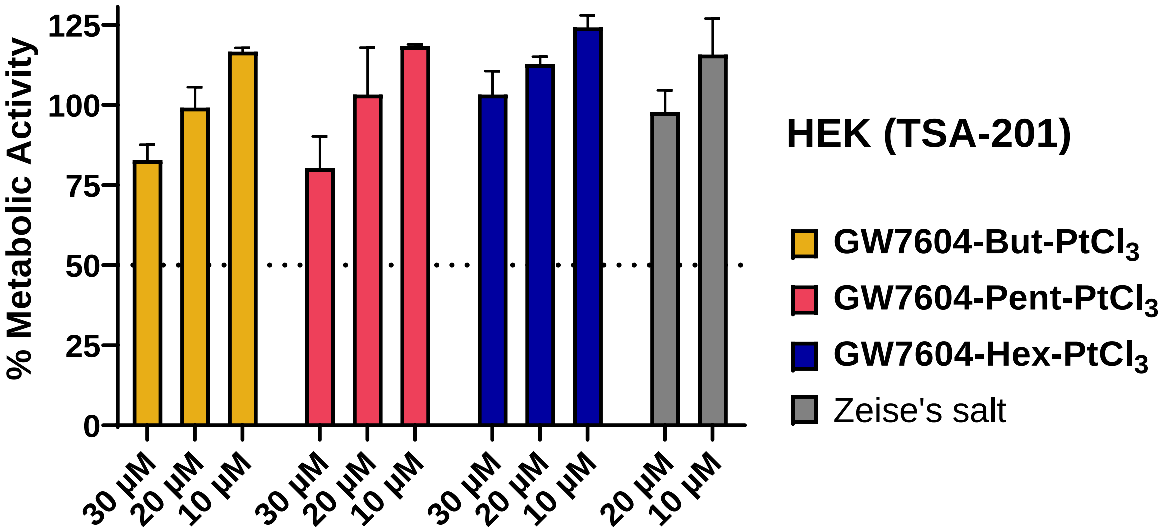

Figure S30. Investigation of metabolic activity in non-cancerous HEK (TSA-201) cells. Cells were incubated for 72 h. The reduction of metabolic activity was measured *via* an MTT assay as the mean of 3 independent experiments ± SD.

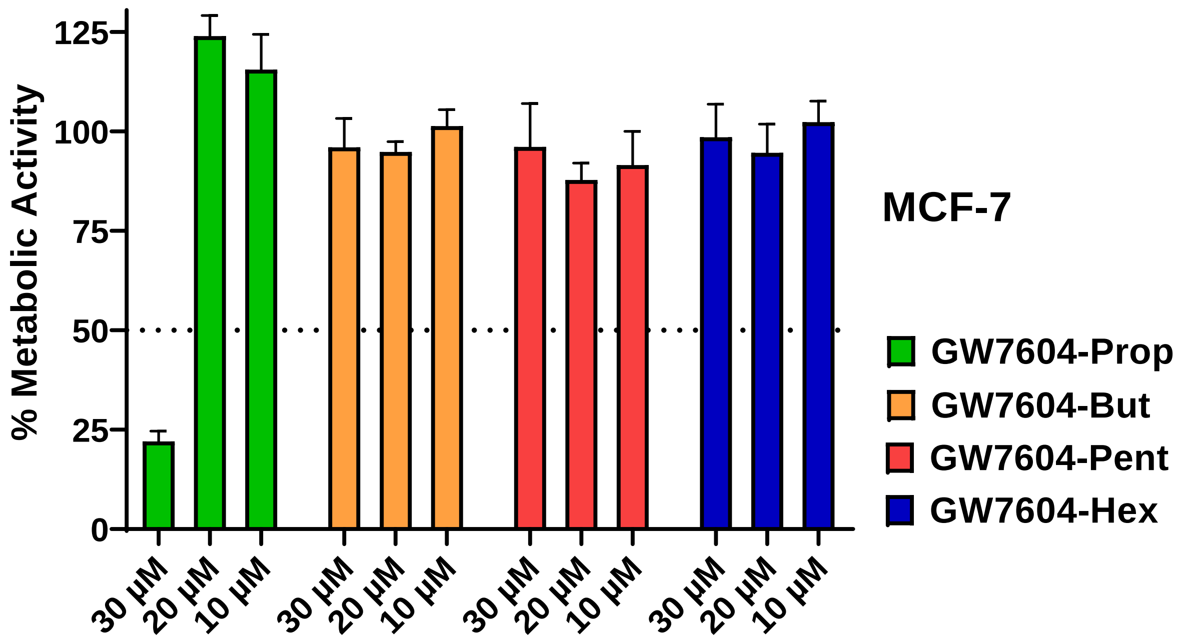

Figure S31. Investigation of metabolic activity in MCF-7 cells. Cells were incubated for 72 h. The reduction of metabolic activity was measured *via* an MTT assay as the mean of 3 independent experiments ± SD.
